# Supplementary material for: Programming ultrasensitive threshold response through chemomechanical instability
Source: Nat Commun. 2021 Aug 30;12:5177. doi: 10.1038/s41467-021-25406-9 (PMC8405678; doi:10.1038/s41467-021-25406-9)
Supplement: Supplementary file 1 — Supplementary information [file 41467_2021_25406_MOESM1_ESM.pdf]

Supplementary Information for

Programming ultrasensitive threshold response through chemo-mechanical instability

Young-Joo Kim, Junho Park, Jae Young Lee and Do-Nyun Kim[*]

**Corresponding E-mail address: dnkim@snu.ac.kr*

Table of Contents

Supplementary Notes 5

Supplementary Note 1. Double-stranded DNA (dsDNA) ring analysis 5

Supplementary Note 2. Finite element analysis of DNA nanostructures 6

Supplementary Note 3. Calculation of mechanical properties of DNA nanostructures...........................7

Supplementary Figures 10

Supplementary Figure 1. An agarose gel electrophoresis image of the purified structures shown in Figure 2b. 10

Supplementary Figure 2. Representative AFM images of the 6HB open ring with respect to EtBr concentrations. 11

Supplementary Figure 3. Representative AFM images of the 6HB open ring with respect to EtBr concentrations. 12

Supplementary Figure 4. Representative AFM images of the 6HB open ring with respect to EtBr concentrations. 13

Supplementary Figure 5. Representative AFM images of the 6HB open ring with respect to EtBr concentrations. 14

Supplementary Figure 6. Detailed experimental data on R_{NC} of the 6HB open rings with respect to EtBr concentrations. 15

Supplementary Figure 7. Representative AFM images of the 6HB closed ring with respect to EtBr concentrations. 16

Supplementary Figure 8. Representative AFM images of the 6HB closed ring with respect to EtBr concentrations. 17

Supplementary Figure 9. Representative AFM images of the 6HB closed ring with respect to EtBr concentrations. 18

Supplementary Figure 10. Representative AFM images of the 6HB closed ring with respect to EtBr concentrations. 19

Supplementary Figure 11. Detailed experimental data on R_{NC} of the 6HB closed rings with respect to EtBr concentrations. 20

Supplementary Figure 12. Representative AFM images of the 10HB open ring with respect to EtBr concentrations. 21

Supplementary Figure 13. Representative AFM images of the 10HB open ring with respect to EtBr concentrations. 22

Supplementary Figure 14. Representative AFM images of the 10HB open ring with respect to EtBr concentrations. 23

Supplementary Figure 15. Detailed experimental data on R_{NC} of the 10HB open rings with respect to EtBr concentrations. 24

Supplementary Figure 16. Representative AFM images of the 10HB closed ring with respect to EtBr concentrations. 25

Supplementary Figure 17. Representative AFM images of the 10HB closed ring with respect to EtBr concentrations. 26

Supplementary Figure 18. Representative AFM images of the 10HB closed ring with respect to EtBr concentrations. 27

Supplementary Figure 19. Detailed experimental data on R_{NC} of the 10HB closed rings with respect to EtBr concentrations. 28

Supplementary Figure 20. Radius of gyration of the structures shown in Figure 2b. 29

Supplementary Figure 21. Reversible reconfiguration of the 6HB closed ring. 30

Supplementary Figure 22. Representative AFM images of the 6HB closed ring with respect to DOX concentrations. 31

Supplementary Figure 23. Representative AFM images of the 6HB closed ring with respect to DOX concentrations. 32

Supplementary Figure 24. Detailed experimental data on R_{NC} of the 6HB closed rings with respect to DOX concentration. 33

Supplementary Figure 25. Representative AFM images of the 6HB closed ring with respect to YOYO-1 concentrations. 34

Supplementary Figure 26. Representative AFM images of the 6HB closed ring with respect to YOYO-1 concentrations. 35

Supplementary Figure 27. Detailed experimental data on R_{NC} of the 6HB closed rings with respect to YOYO-1 concentration. 36

Supplementary Figure 28. Agarose gel electrophoresis results of the purified structures shown in Figure 3b. 37

Supplementary Figure 29. Representative AFM images of the 6HB closed ring with 1-nt gap with respect to EtBr concentrations. 38

Supplementary Figure 30. Representative AFM images of the 6HB closed ring with 1-nt gap with respect to EtBr concentrations. 39

Supplementary Figure 31. Representative AFM images of the 6HB closed ring with 1-nt gap with respect to EtBr concentrations. 40

Supplementary Figure 32. Representative AFM images of the 6HB closed ring with 1-nt gap with respect to EtBr concentrations. 41

Supplementary Figure 33. Detailed experimental data on R_{NC} of the 6HB closed rings with 1-nt gap. . 42

Supplementary Figure 34. Representative AFM images of the 6HB closed ring with 3-nt gap with respect to EtBr concentrations. 43

Supplementary Figure 35. Representative AFM images of the 6HB closed ring with 3-nt gap with respect to EtBr concentrations. 44

Supplementary Figure 36. Representative AFM images of the 6HB closed ring with 3-nt gap with respect to EtBr concentrations. 45

Supplementary Figure 37. Representative AFM images of the 6HB closed rings with 3-nt gap with respect to EtBr concentrations. 46

Supplementary Figure 38. Detailed experimental data on R_{NC} of the 6HB closed rings with 3-nt gap. . 47

Supplementary Figure 39. Representative AFM images of the 6HB open ring with gap with respect to EtBr concentrations. 48

Supplementary Figure 40. Detailed experimental data on R_{NC} of the 6HB open rings with gap. 49

Supplementary Figure 41. Agarose gel electrophoresis results of the structures shown in Figure 4a. ... 50

Supplementary Figure 42. Representative AFM images of the 6HB closed triangle with respect to EtBr concentrations. 51

Supplementary Figure 43. Representative AFM images of the 6HB closed triangle with respect to EtBr concentrations. 52

Supplementary Figure 44. Representative AFM images of the 6HB closed triangle with respect to EtBr concentrations. 53

Supplementary Figure 45. Representative AFM images of the 6HB closed triangle with respect to EtBr concentrations. 54

Supplementary Figure 46. Detailed experimental data on R_{NC} of the 6HB closed triangles. 55

Supplementary Tables: List of staple sequences 56

Supplementary Table 1. DNA sequence for the 6HB closed ring structure. 56

Supplementary Table 2. DNA sequence for the 6HB closed ring structure with 1-nt gap. 61

Supplementary Table 3. DNA sequence for the 6HB closed ring structure with 3-nt gap. 66

Supplementary Table 4. DNA sequence for the 10HB closed ring structure. 71

Supplementary Table 5. DNA sequence for the 6HB closed triangle structure. 75

Supplementary References 80

Supplementary Notes

Supplementary Note 1. Double-stranded DNA (dsDNA) ring analysis

To understand the mechanics of Michell's instability, a finite element analysis was performed for the idealized dsDNA ring which consists of 336 base-pairs (BPs) using SNUPI (Structured NUcleic-acids Programming Interface)[1]. The configuration of each BP was abstracted into a node and triad, and the connection of two successive BPs was substituted by Euler-Bernoulli beam finite element. In the model, the dsDNA was assumed to be intrinsically straight and have the regular geometry of B-form DNA (axial rise of 0.34 nm and helicity of 10.5 BP per turn) and mechanical properties (stretching rigidity (S) of 1100 pN, bending rigidity (B) of 230 pN nm^2, and torsional rigidity (C) of 460 pN nm^2). Here, the coupling coefficients were ignored. The bending moment was applied at both ends of a stress-free straight dsDNA to construct the initial configuration of the dsDNA ring structure. After fixing one end, the torsional displacement (θ) was applied at the other end while its other degree of freedom was also fixed. As the θ is increased, the configuration of the dsDNA ring structure was calculated through nonlinear static analysis with only geometrical nonlinearity until self-contact of the structure occurs. In each incremental step, the stretching (π_S), bending (π_B), and torsional (π_C) strain energies of a finite element were calculated as $\pi_S = S\Delta^2/2L$, $\pi_B = B\varphi^2/2L$, and $\pi_C = C\omega^2/2L$, where L, Δ, φ, and ω indicate the axial length, the length change, the bending angle, and the twist angle, respectively. Its total strain energy was obtained by summing these energies.

Supplementary Note 2. Finite element analysis of DNA nanostructures

We carried out a finite element (FE) simulation for DNA nanostructures to predict their equilibrium shapes using SNUPI (Structured Nucleic acids Programming Interface), which would be provided in our recent work[1]. From caDNAno design files,[2] SNUPI obtains information on connectivity and mechanical perturbation among BPs, and constructs FE model, where a BP was abstracted into a node and two successive BPs were connected by a beam finite element. In the FE model, the DNA duplex was assumed to have the regular B-form DNA geometry (diameter of 2.25 nm, axial rise of 0.32 nm and helicity of 10.5 BP per turn) with S of 1100 pN, B of 230 pN nm^2, C of 460 pN nm^2 and torsional-stretch coupling (g) of -180 pNnm. Note that we did not consider any sequence-dependent properties and assumed a nicked DNA duplex to be the same with a regular DNA duplex in terms of its geometrical and mechanical properties. Crossovers were modeled by a beam finite element that connects two BPs that belong to two adjacent helices. Their mechanical stiffness was defined by multiplying a scale factor (SF) to those of DNA as 1 for S, 0.12 for B, and 0.1 for C, respectively. To simulate the unwinding effect induced by EtBr binding on structural shape, in addition, the equilibrated configuration of the structure was obtained through the nonlinear static analysis with increasing the helicity of dsDNA (BP/turn). In each incremental step, the total strain energy (π) was calculated by summing the strain energy of each finite element as $\pi = \Sigma\pi^{(e)}$ with $\pi = S(\Delta^{(e)})^2/2L^{(e)} + B(\varphi^{(e)})^2/2L^{(e)} + C(\omega^{(e)})^2/2L^{(e)} + g\Delta^{(e)}\omega^{(e)}/2L^{(e)}$.

Supplementary Note 3. Calculation of mechanical properties of DNA nanostructures

Normal mode analysis (NMA) was performed at the straight configuration to compute the lowest 40 normal modes of DNA nanostructures. Given the FE model for a DNA nanostructure under free boundary condition, a generalized eigenvalue problem is considered as follows.

$$\mathrm{Ku} = \lambda \mathrm{Mu} \tag{1}$$

where K is the global stiffness matrix, M is the global mass matrix and λ represents the eigenvalue as the square-root of natural frequency ($\lambda = \omega^2$). Among the eigenvalues obtained, only the two eigenvalues for the first bending modes were selected to calculate bending rigidity (EI).

From the Euler-Lagrange equation for a beam representing effectively DNA nanostructures, we could obtain the following free vibration equation.

$$\mathrm{EI}\frac{d^4 w}{dx^4} + \mu\omega^2 w = 0 \tag{2}$$

where w describes the lateral deflection of the beam, x represents the axial position, and μ is the mass per unit length of the beam. Solving analytically the above equation, we can obtain the natural frequencies of the first bending vibration (ω_B) as

$$\omega_B = \frac{4.733^2}{L^2}\sqrt{\frac{\mathrm{EI}}{\mu}} \tag{3}$$

where L is the length of the beam. Then, EI of the beam becomes

$$\mathrm{EI} = \mu\omega_B^2 \frac{L^4}{4.733^4} = \frac{m}{L}\lambda_B^2 \frac{L^4}{4.733^4} = m\lambda_B^2 \frac{L^3}{4.733^4} \tag{4}$$

where m is the total mass of DNA nanostructures, and λ_B is the eigenvalues for the first bending mode. Since the L_p is defined as

$$L_p = \frac{EI}{k_B T} \tag{5}$$

where k_B is the Boltzmann constant and T is temperature, assumed to be 300 K. Then, L_p of DNA nanostructures becomes

$$L_p = m\lambda_B^2 \frac{L^3}{4.733^4 k_B T} \tag{6}$$

In a three-dimensional structure, there are always two first bending modes. Therefore, we defined the first bending mode with a smaller eigenvalue as a major bending mode ($L_{p,1}$) and the other with a larger eigenvalue as a minor bending mode ($L_{p,2}$). Using both bending modes, also, we defined an effective bending persistence length ($L_{p,e}$), which we compared with the measured one from AFM image analysis.

$$L_{p,e} = \frac{2}{1/L_{p,1} + 1/L_{p,2}} \tag{7}$$

Also, introducing the continuum assumption provides the torsional rigidity of the DNA nanostructure from the eigenvalues by NMA. The governing equation was given as

$$GJ\frac{\partial^2\theta}{\partial x^2} + \rho J_o \omega^2 \theta = 0 \tag{8}$$

where θ denotes the torsional angle as a function of the beam axis, ρ is the volume density as m/(AL) with the cross-section area A. The solution of the governing equation yields the characteristic equation given by

$$GJ\beta^2 = \rho J_o \omega^2 \tag{9}$$

where β is the wavenumber. In free-free end boundary condition, the wavenumber satisfies a relation with the lowest mode number as

$$\sin(\beta L) = 0, \quad \beta L = \pi \tag{10, 11}$$

Accordingly, the torsional persistence length (L_c) was derived as follows.

$$L_c = \frac{GJ}{k_B T} = \frac{m\omega^2 L}{k_B T \pi^2}\left(\frac{J_o}{A}\right) \tag{12}$$

where the ratio of the polar moment of inertia to the area (J_o/A) can be pre-determined by the cross-sectional geometry of the DNA nanostructure.

Supplementary Figures

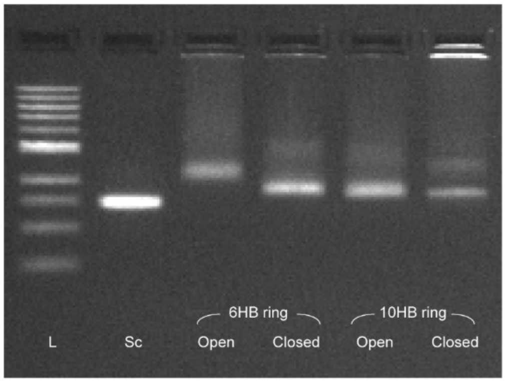

Supplementary Figure 1. An agarose gel electrophoresis image of the purified structures shown in Figure 2b. A clear monomer band was observed in all structures. L: 1kb DNA ladder and Sc: Scaffold strand.

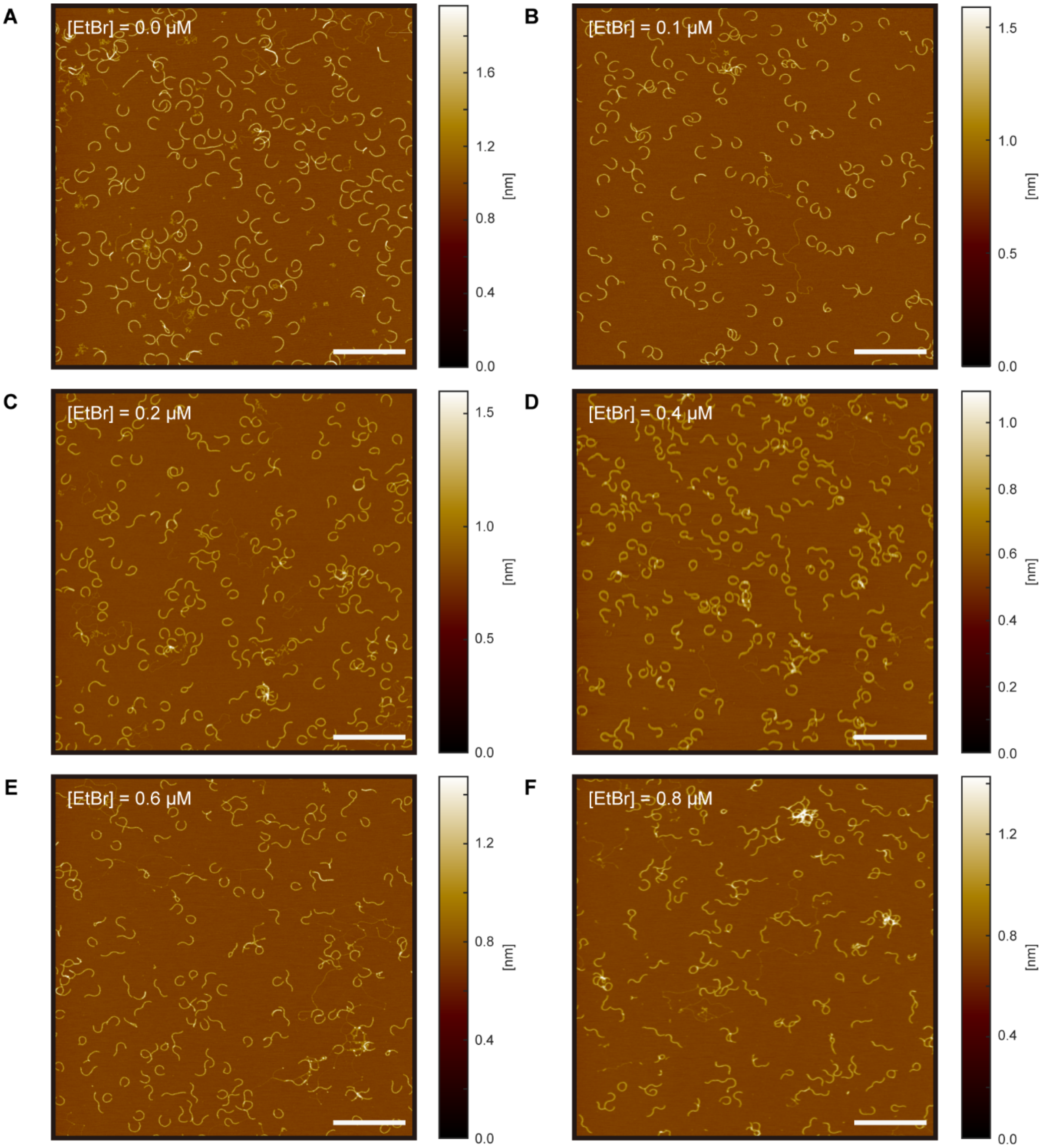

Supplementary Figure 2. Representative AFM images of the 6HB open ring with respect to EtBr concentrations. AFM images for samples shown in Fig. 2b. Scale bars: 1 μm.

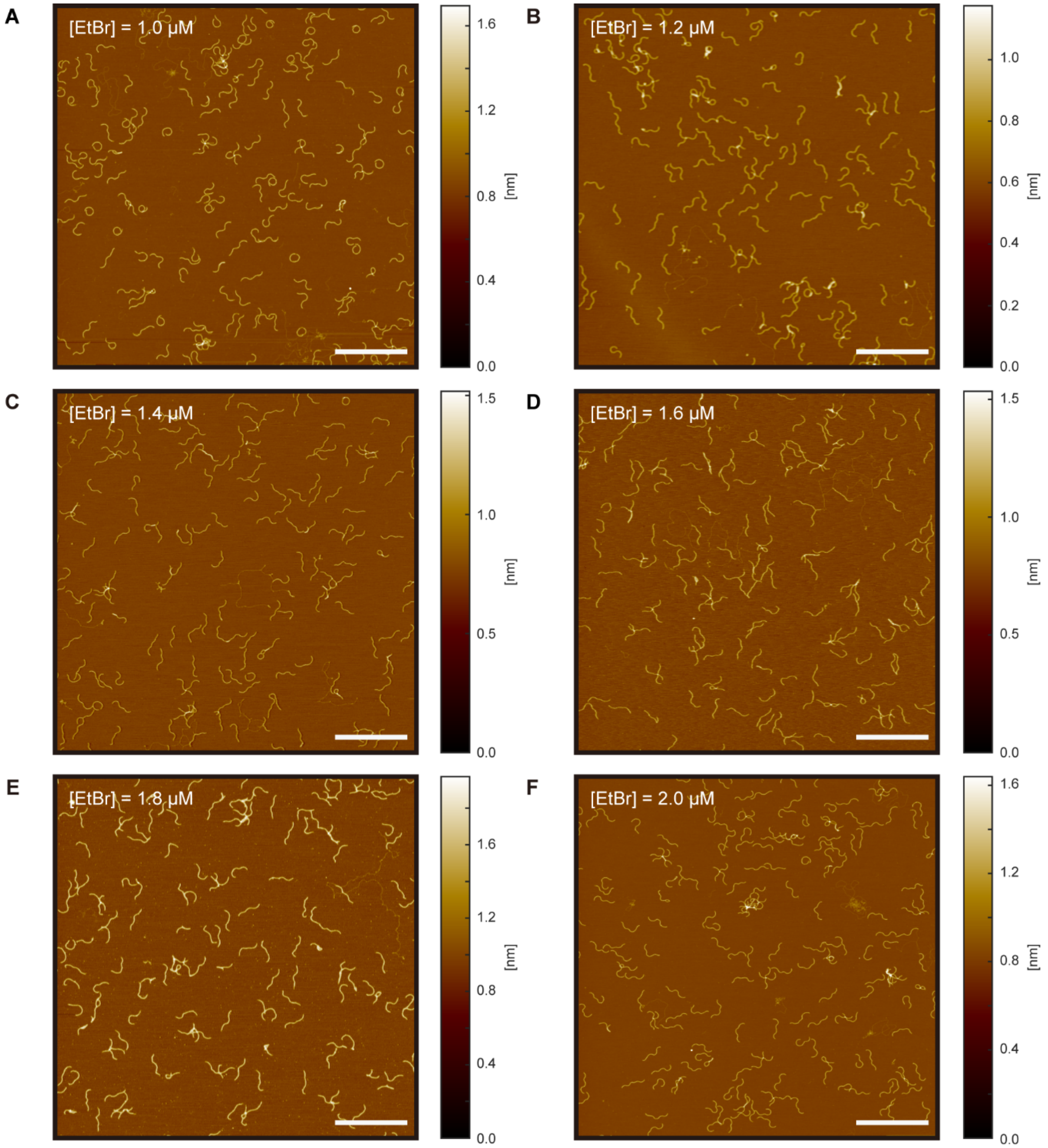

Supplementary Figure 3. Representative AFM images of the 6HB open ring with respect to EtBr concentrations. AFM images for samples shown in Fig. 2b. Scale bars: 1 μm.

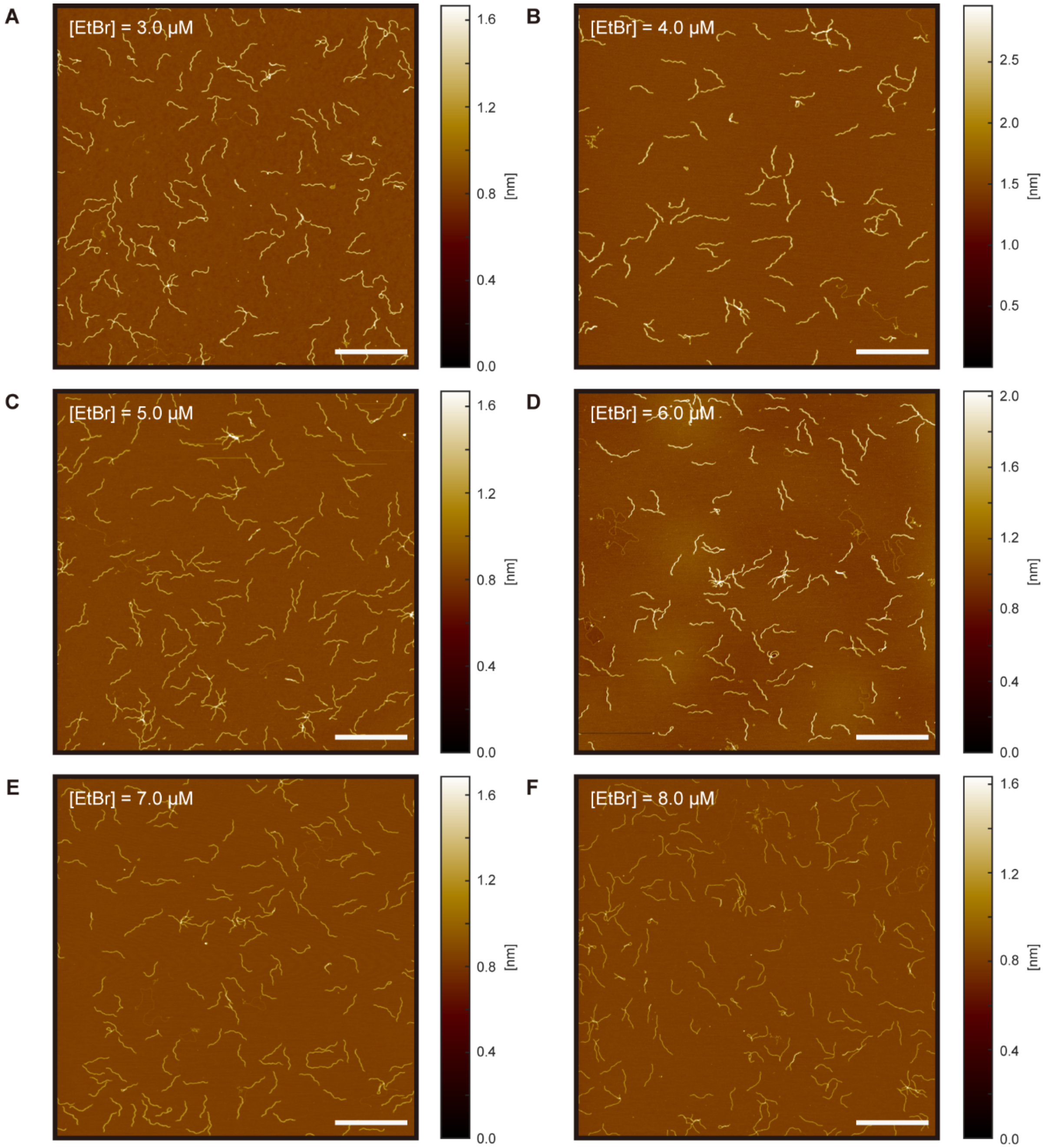

Supplementary Figure 4. Representative AFM images of the 6HB open ring with respect to EtBr concentrations. AFM images for samples shown in Fig. 2b. Scale bars: 1 μm.

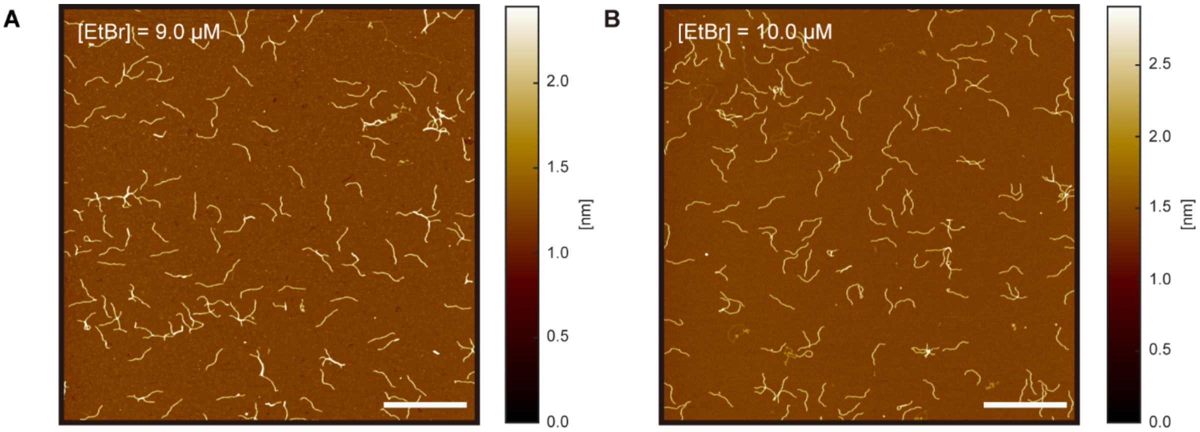

Supplementary Figure 5. Representative AFM images of the 6HB open ring with respect to EtBr concentrations. AFM images for samples shown in Fig. 2b. Scale bars: 1 μm.

Concentration of EtBr [μM]	0.0	0.1	0.2	0.4	0.6
Number of samples	488	585	667	640	509
Average	0.0983	0.2569	0.3808	0.5014	0.5189
Standard deviation	0.0135	0.0180	0.0187	0.0198	0.0224

Concentration of EtBr [μM]	0.8	1.0	1.2	1.4	1.6
Number of samples	452	457	555	517	473
Average	0.7700	0.8361	0.9441	0.9672	0.9831
Standard deviation	0.0197	0.0172	0.0097	0.0078	0.0060

Concentration of EtBr [μM]	1.8	2.0	3.0	4.0	5.0
Number of samples	559	484	497	466	509
Average	0.9857	1.0000	1.0000	0.9978	1.0000
Standard deviation	0.0050	0.0000	0.0000	0.0022	0.0000

Concentration of EtBr [μM]	6.0	7.0	8.0	9.0	10.0
Number of samples	474	443	425	438	466
Average	1.0000	1.0000	1.0000	1.0000	1.0000
Standard deviation	0.0000	0.0000	0.0000	0.0000	0.0000

Supplementary Figure 6. Detailed experimental data on R_{NC} of the 6HB open rings with respect to EtBr concentrations. Standard deviation of the R_{NC} was calculated by a bootstrap method with a subset of the given number of samples randomly chosen with replacement and 10,000 repeats of the process.

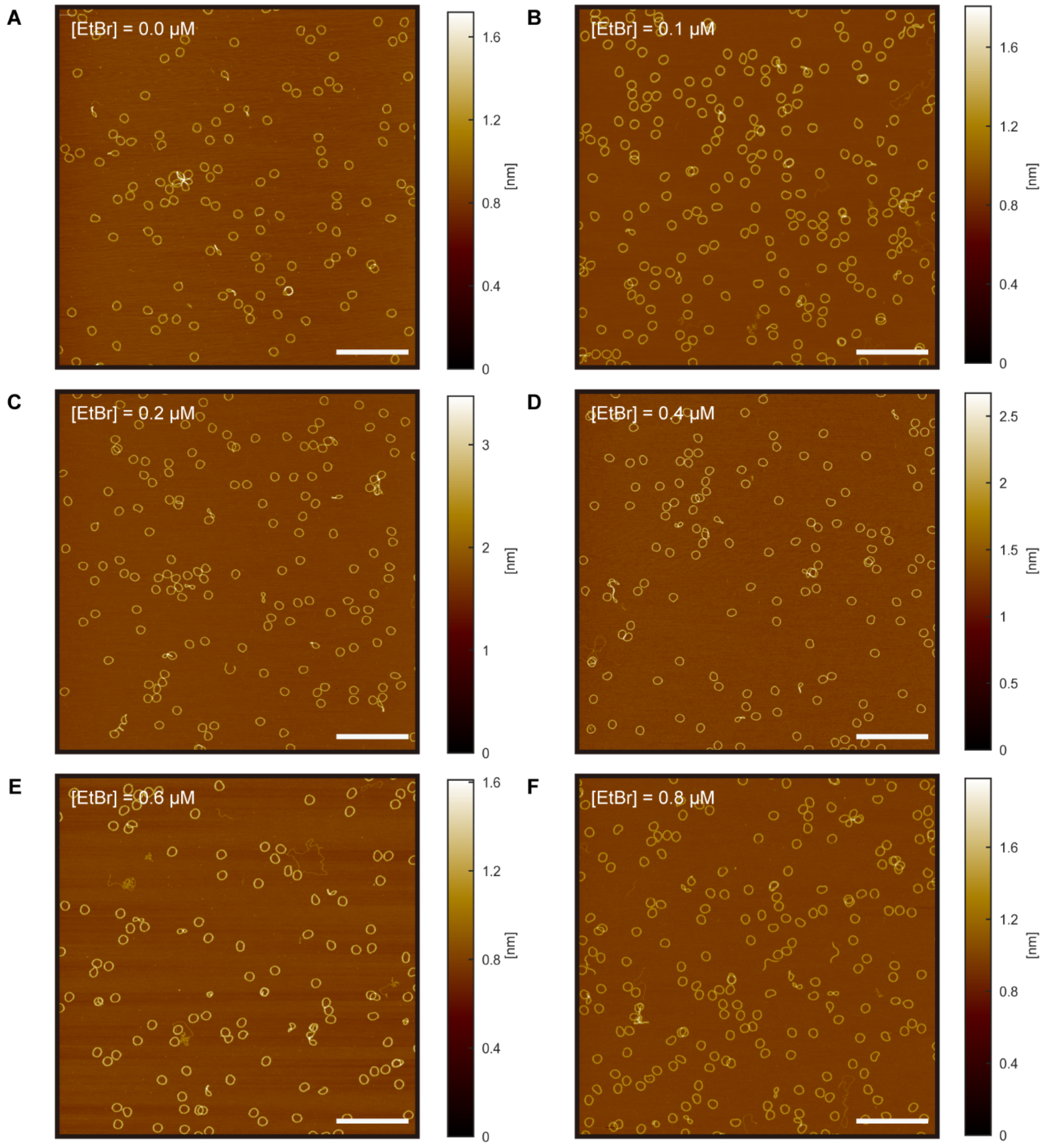

Supplementary Figure 7. Representative AFM images of the 6HB closed ring with respect to EtBr concentrations. AFM images for samples shown in Fig. 2b. Scale bars: 1 μm.

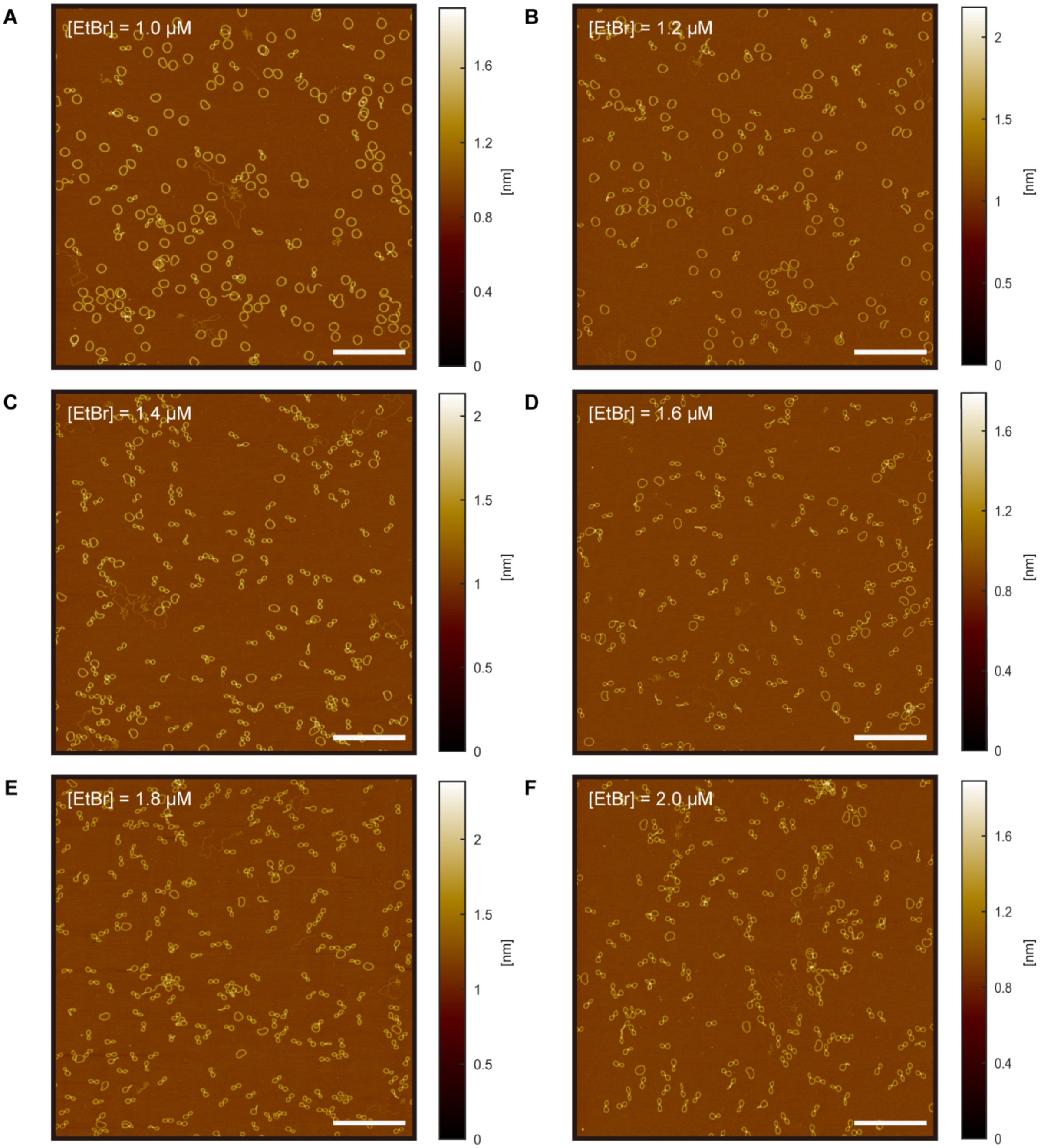

Supplementary Figure 8. Representative AFM images of the 6HB closed ring with respect to EtBr concentrations. AFM images for samples shown in Fig. 2b. Scale bars: 1 μm.

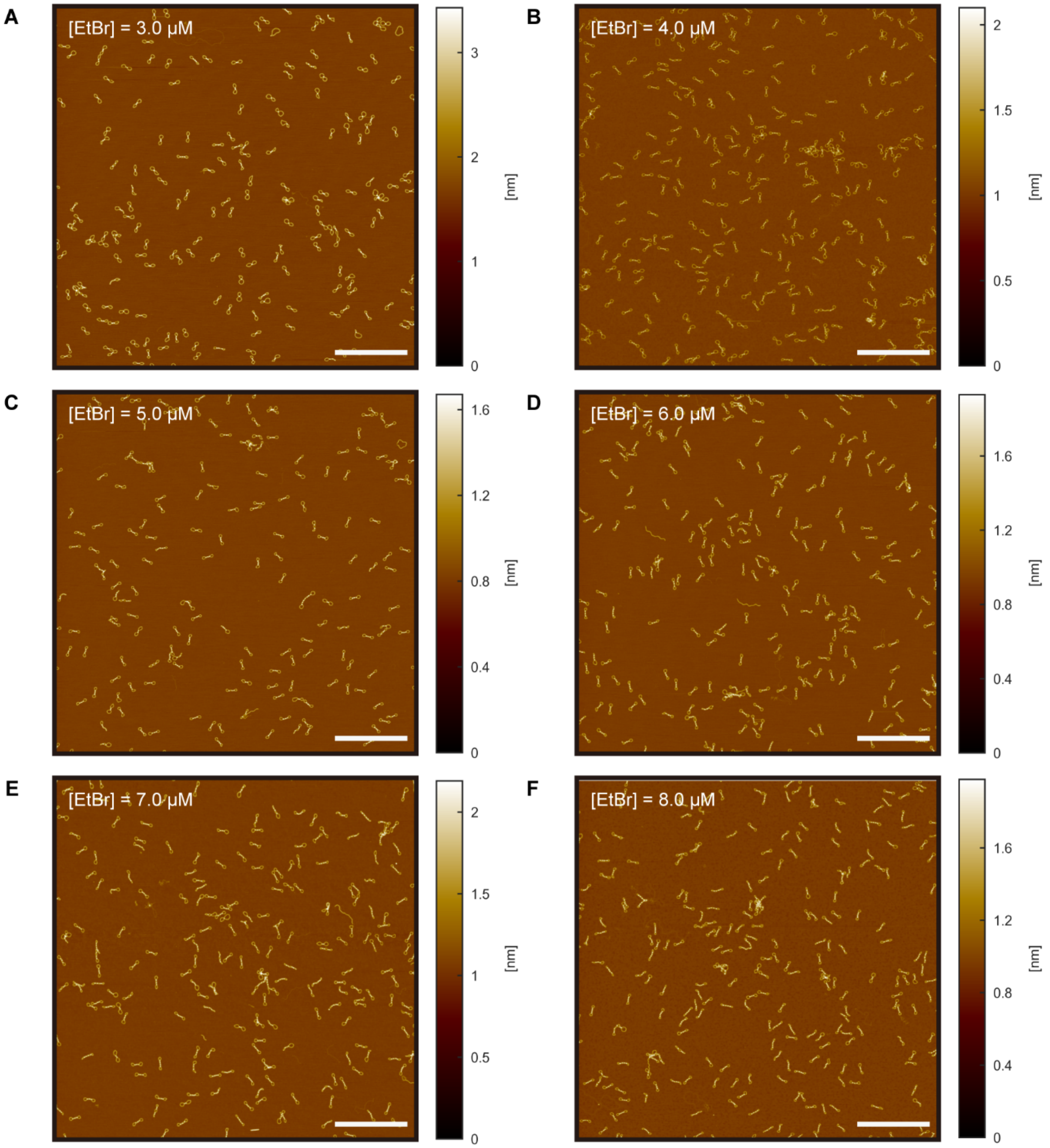

Supplementary Figure 9. Representative AFM images of the 6HB closed ring with respect to EtBr concentrations. AFM images for samples shown in Fig. 2b. Scale bars: 1 μm.

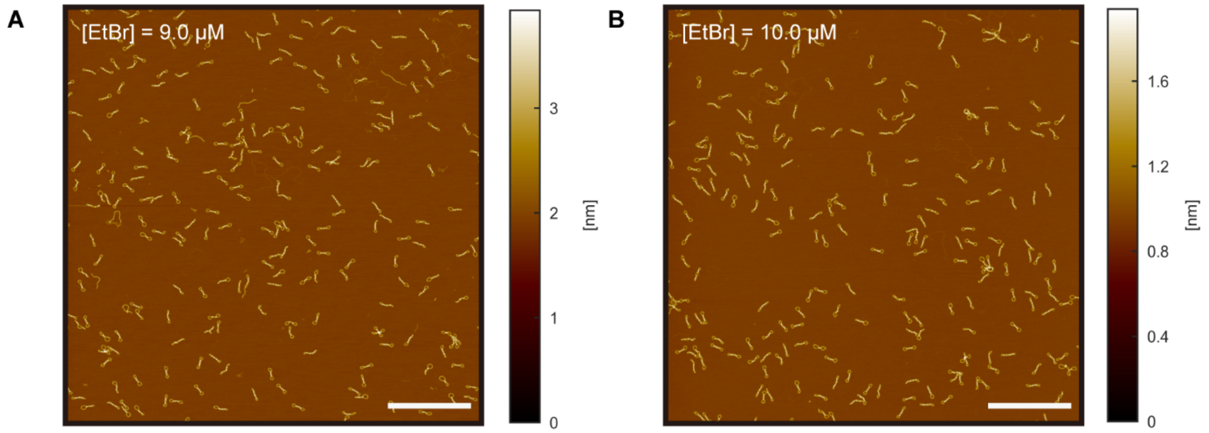

Supplementary Figure 10. Representative AFM images of the 6HB closed ring with respect to EtBr concentrations. AFM images for samples shown in Fig. 2b. Scale bars: 1 μm.

Concentration of EtBr [μM]	0.0	0.1	0.2	0.4	0.6
Number of samples	1116	649	1072	943	531
Average	0.0144	0.0031	0.0155	0.0053	0.0169
Standard deviation	0.0036	0.0022	0.0039	0.0024	0.0056

Concentration of EtBr [μM]	0.8	1.0	1.2	1.4	1.6
Number of samples	673	840	891	871	670
Average	0.0342	0.5831	0.6867	0.8901	0.8939
Standard deviation	0.0070	0.0171	0.0153	0.0104	0.0119

Concentration of EtBr [μM]	1.8	2.0	3.0	4.0	5.0
Number of samples	939	829	792	740	779
Average	0.9637	0.9626	0.9975	0.9973	1.0000
Standard deviation	0.0061	0.0066	0.0018	0.0019	0.0000

Concentration of EtBr [μM]	6.0	7.0	8.0	9.0	10.0
Number of samples	985	742	844	612	840
Average	0.9990	1.0000	0.9988	0.9951	0.9988
Standard deviation	0.0010	0.0000	0.0012	0.0028	0.0012

Supplementary Figure 11. Detailed experimental data on R_{NC} of the 6HB closed rings with respect to EtBr concentrations. Standard deviation of the R_{NC} was calculated by a bootstrap method with a subset of the given number of samples randomly chosen with replacement and 10,000 repeats of the process.

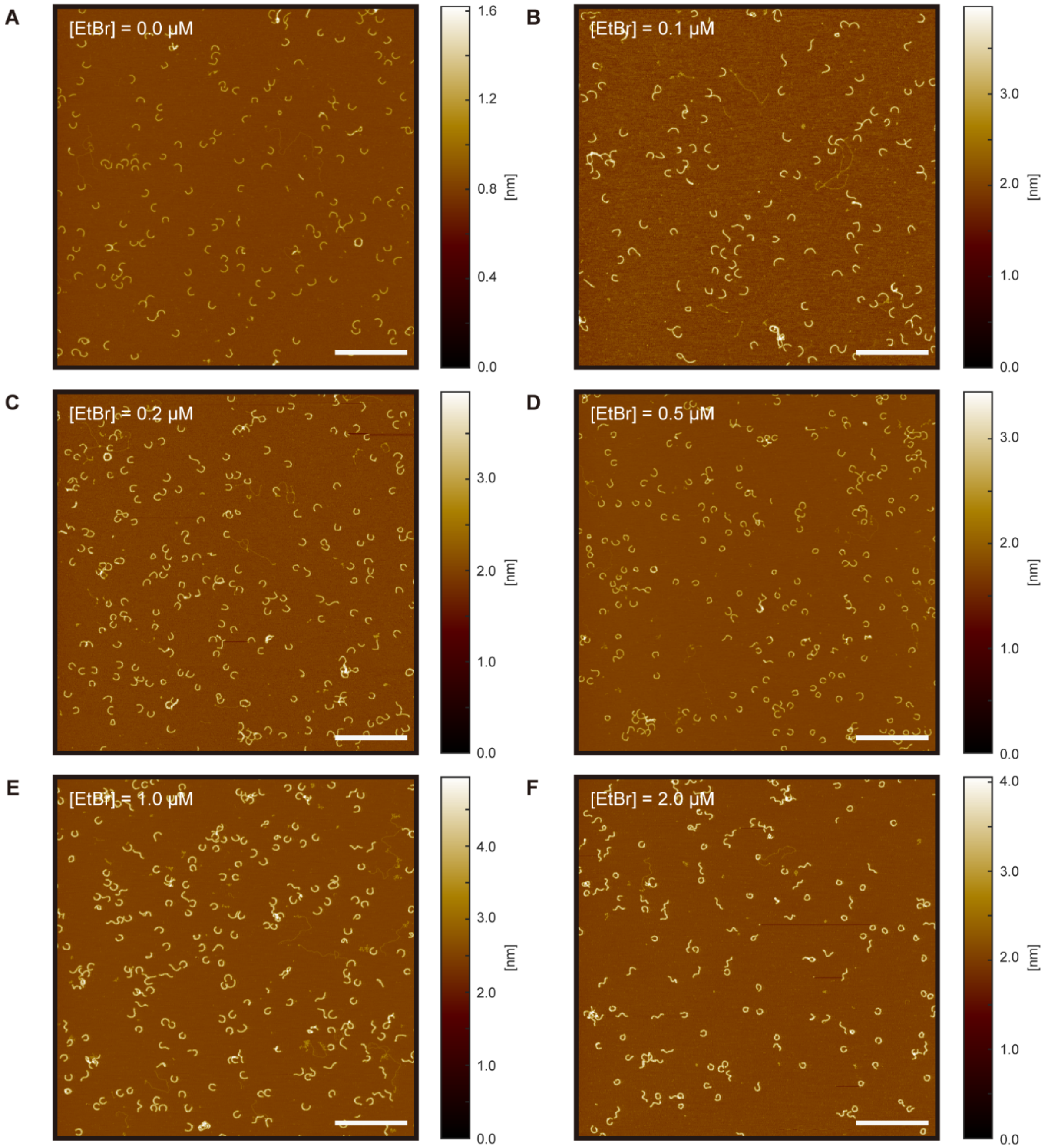

Supplementary Figure 12. Representative AFM images of the 10HB open ring with respect to EtBr concentrations. AFM images for samples shown in Fig. 2b. Scale bars: 1 μm.

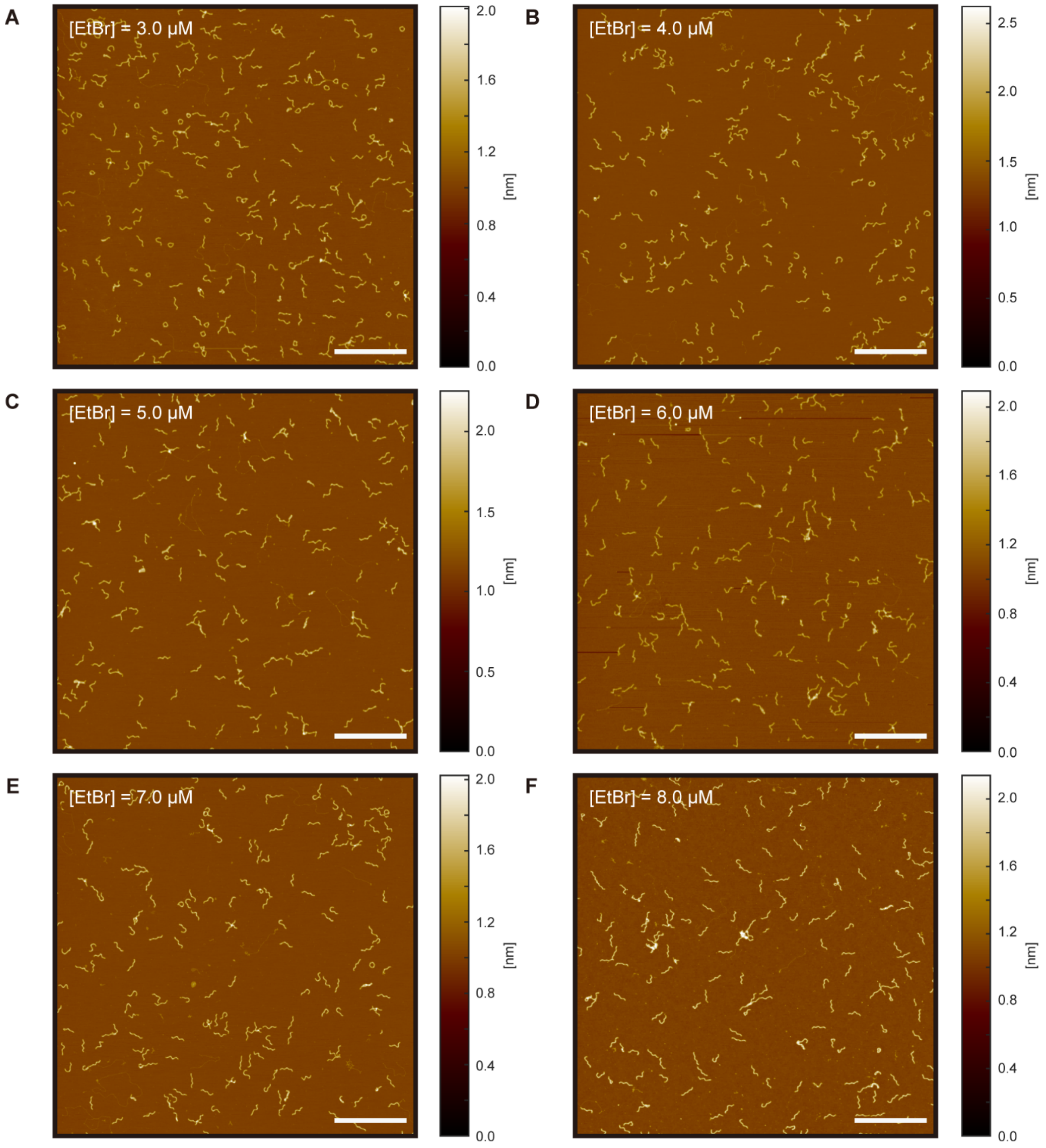

Supplementary Figure 13. Representative AFM images of the 10HB open ring with respect to EtBr concentrations. AFM images for samples shown in Fig. 2b. Scale bars: 1 μm.

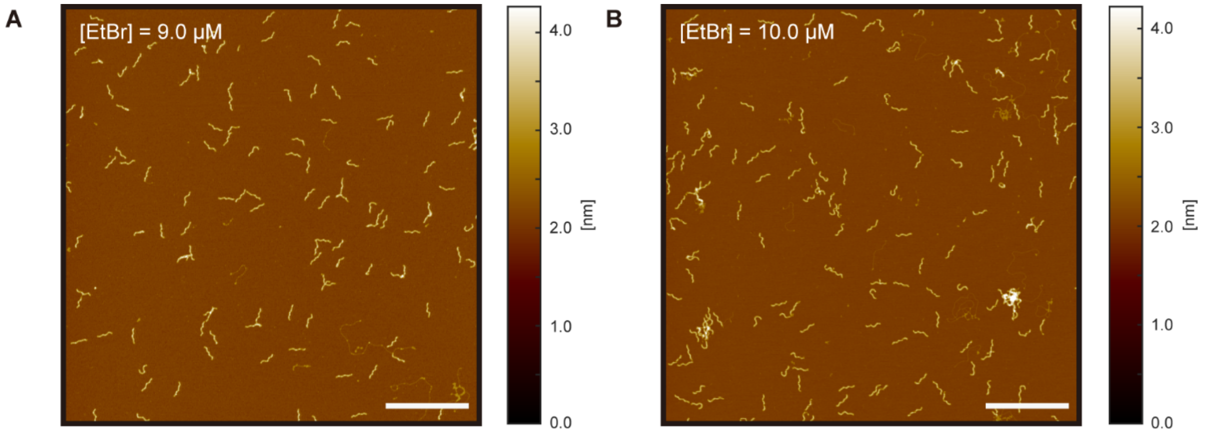

Supplementary Figure 14. Representative AFM images of the 10HB open ring with respect to EtBr concentrations. AFM images for samples shown in Fig. 2b. Scale bars: 1 μm.

Concentration of EtBr [μM]	0.0	0.1	0.2	0.5	1.0
Number of samples	691	586	798	597	726
Average	0.0101	0.090	0.0613	0.1359	0.2425
Standard deviation	0.0038	0.0080	0.0084	0.0141	0.0159

Concentration of EtBr [μM]	2.0	3.0	4.0	5.0	6.0
Number of samples	726	784	606	519	578
Average	0.5231	0.8123	0.9092	0.9633	0.9776
Standard deviation	0.0187	0.0138	0.0117	0.0082	0.0061

Concentration of EtBr [μM]	7.0	8.0	9.0	10.0
Number of samples	657	459	456	435
Average	0.9819	1.0000	0.9978	1.0000
Standard deviation	0.0052	0.0000	0.0022	0.0000

Supplementary Figure 15. Detailed experimental data on R_{NC} of the 10HB open rings with respect to EtBr concentrations. Standard deviation of the R_{NC} was calculated by a bootstrap method with a subset of the given number of samples randomly chosen with replacement and 10,000 repeats of the process.

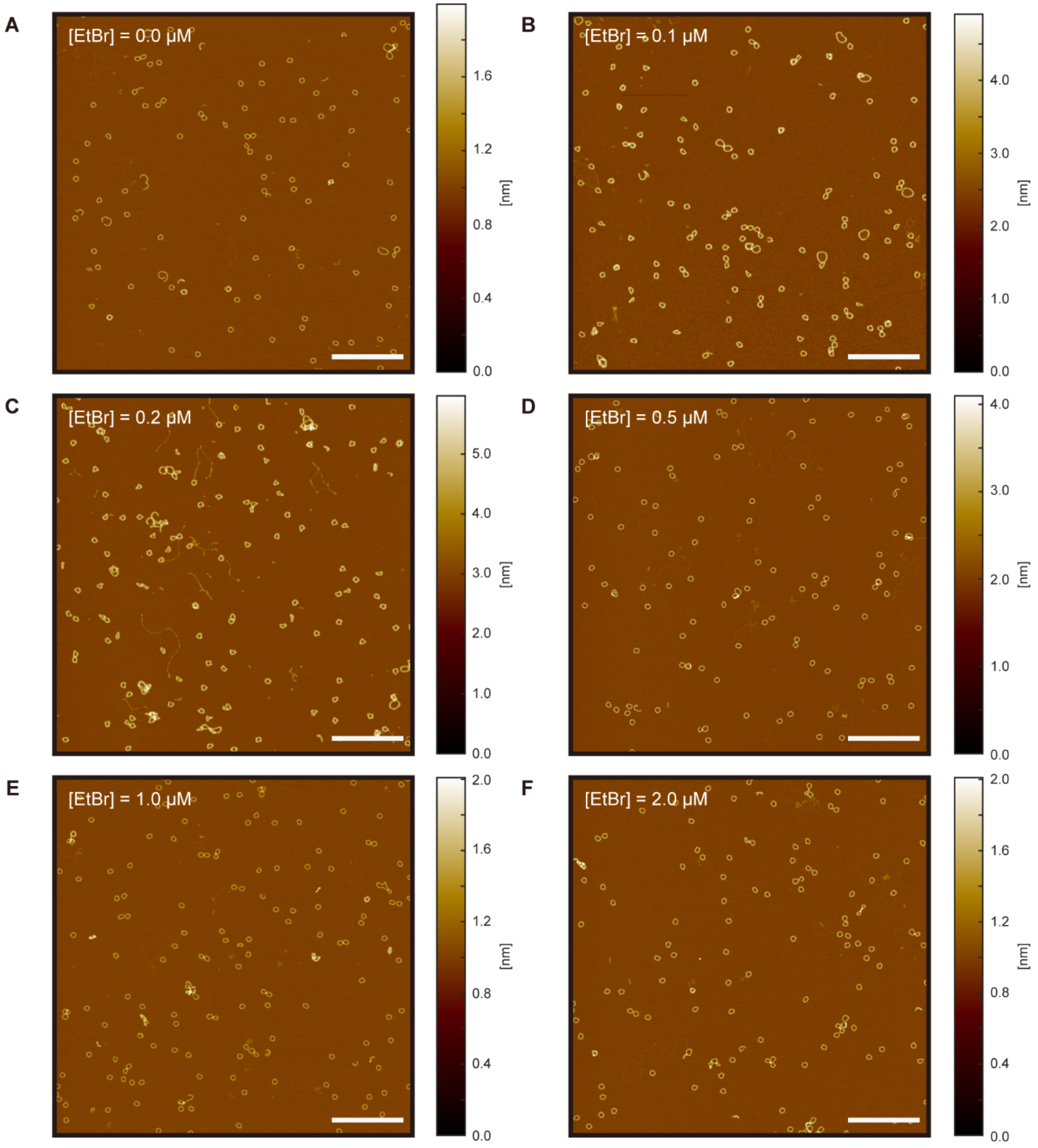

Supplementary Figure 16. Representative AFM images of the 10HB closed ring with respect to EtBr concentrations. AFM images for samples shown in Fig. 2b. Scale bars: 1 μm.

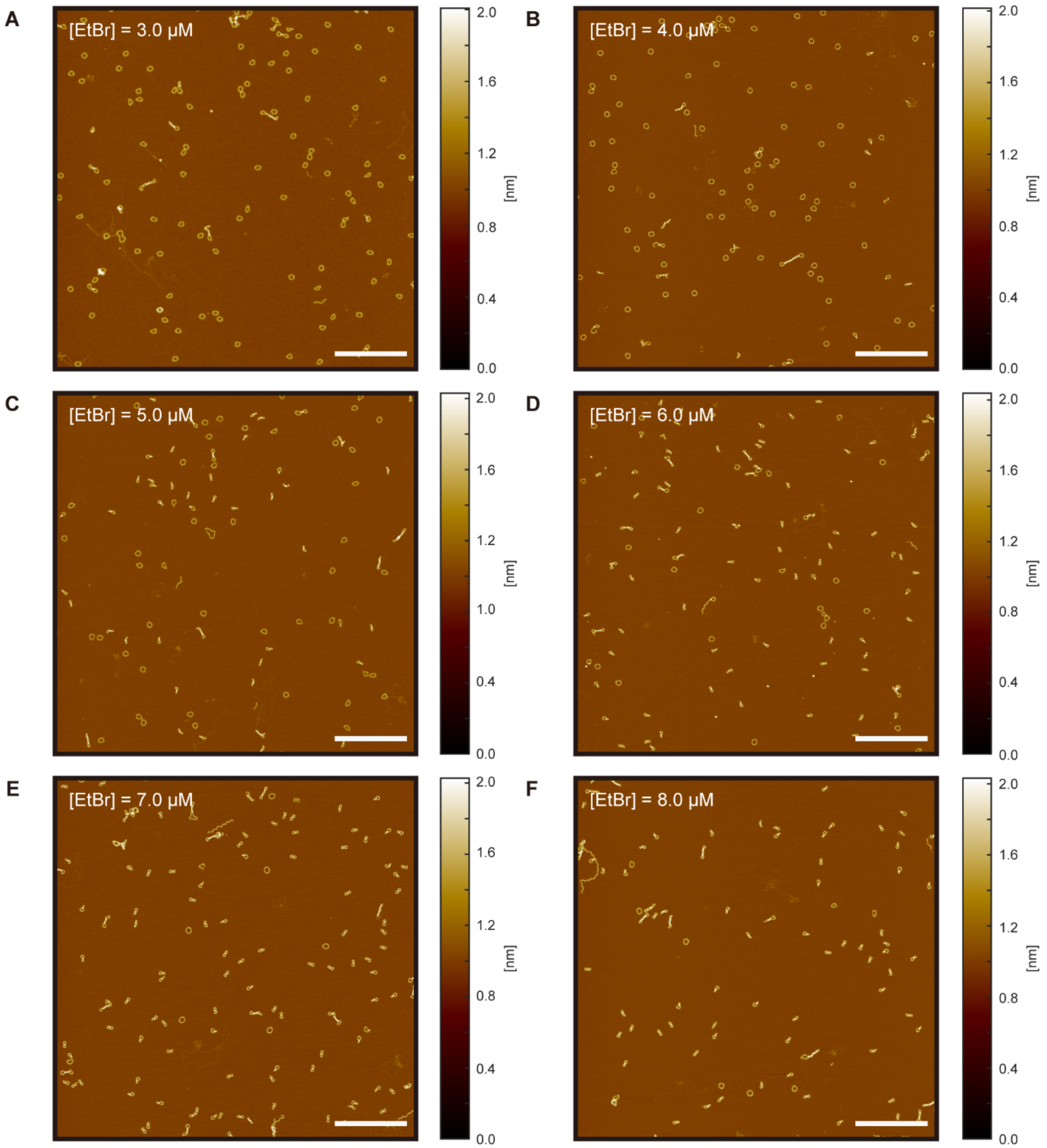

Supplementary Figure 17. Representative AFM images of the 10HB closed ring with respect to EtBr concentrations. AFM images for samples shown in Fig. 2b. Scale bars: 1 μm.

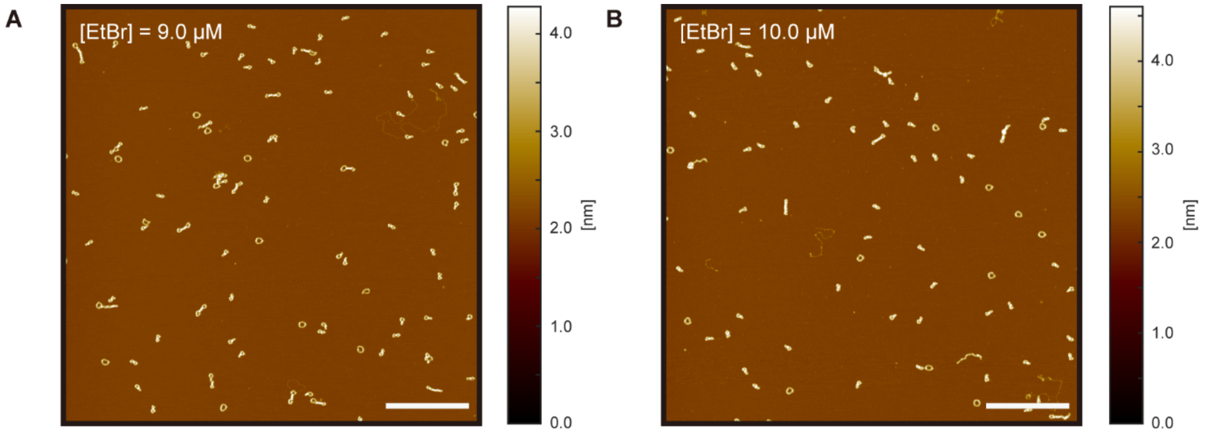

Supplementary Figure 18. Representative AFM images of the 10HB closed ring with respect to EtBr concentrations. AFM images for samples shown in Fig. 2b. Scale bars: 1 μm.

Concentration of EtBr [μM]	0.0	0.1	0.2	0.5	1.0
Number of samples	563	499	537	577	552
Average	0.0001	0.0001	0.0019	0.0001	0.0001
Standard deviation	0.0000	0.0000	0.0019	0.0000	0.0000

Concentration of EtBr [μM]	2.0	3.0	4.0	5.0	6.0
Number of samples	552	445	523	560	495
Average	0.0001	0.0145	0.1357	0.4286	0.7617
Standard deviation	0.0000	0.0064	0.0150	0.0210	0.0193

Concentration of EtBr [μM]	7.0	8.0	9.0	10.0
Number of samples	531	433	459	393
Average	0.8986	0.8775	0.8369	0.8678
Standard deviation	0.0132	0.0157	0.0173	0.0170

Supplementary Figure 19. Detailed experimental data on R_{NC} of the 10HB closed rings with respect to EtBr concentrations. Standard deviation of the R_{NC} was calculated by a bootstrap method with a subset of the given number of samples randomly chosen with replacement and 10,000 repeats of the process.

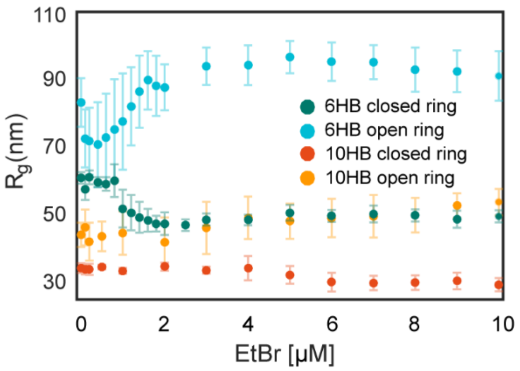

Supplementary Figure 20. Radius of gyration of the structures shown in Figure 2b. Radius of gyration (R_g) values, experimentally measured from all monomers in AFM images. Circles and error bars represent the mean of and the standard deviation of R_g, respectively.

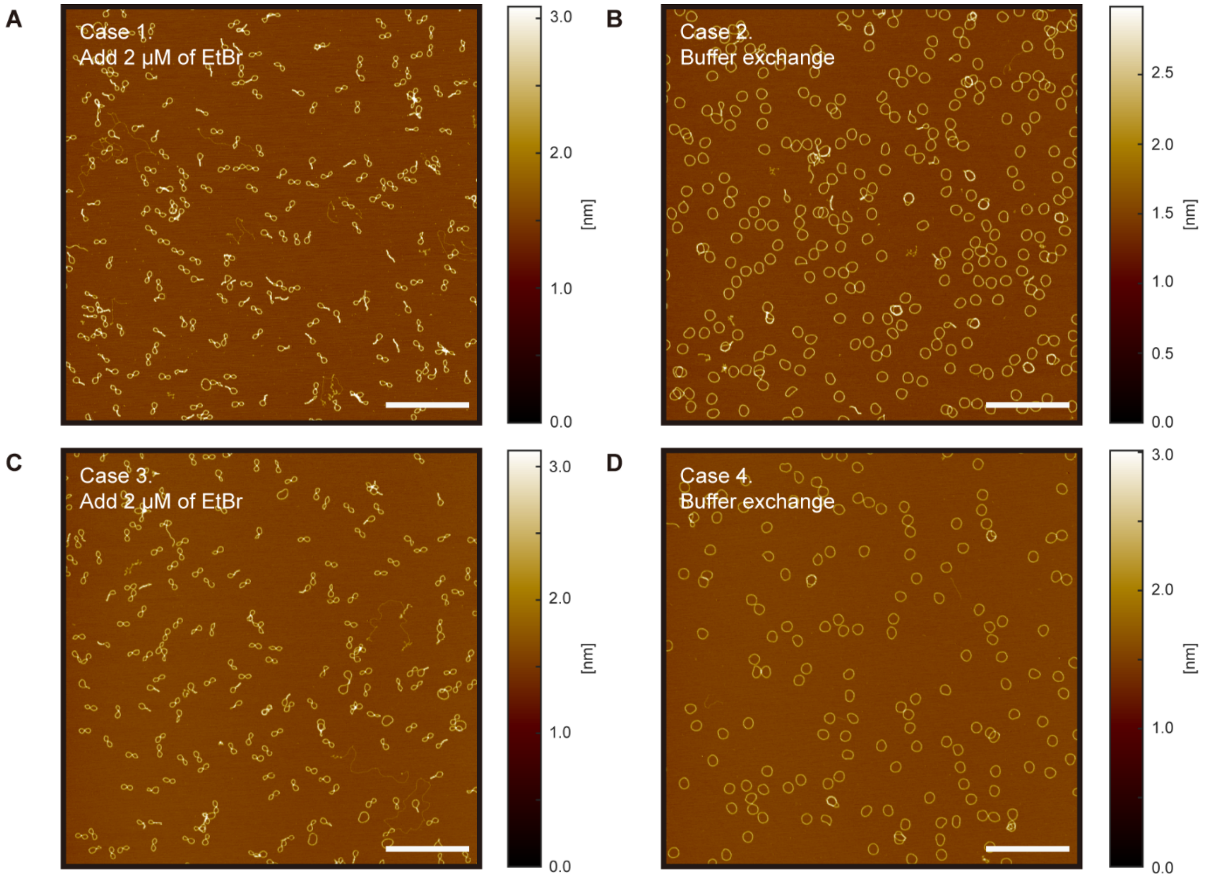

Supplementary Figure 21. Reversible reconfiguration of the 6HB closed ring. AFM images for samples shown in Fig. 2f. Scale bars: 1 μm.

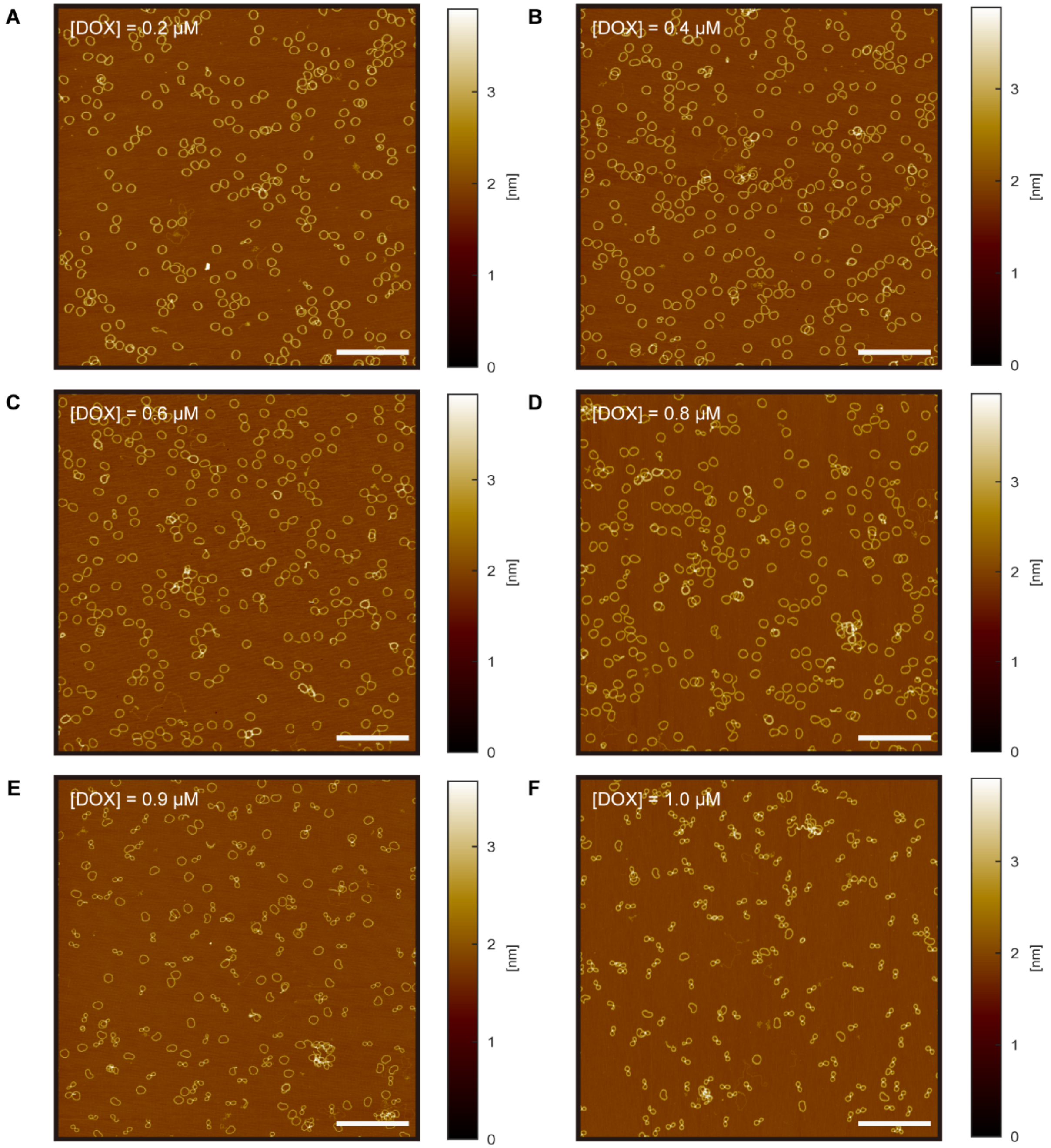

Supplementary Figure 22. Representative AFM images of the 6HB closed ring with respect to DOX concentrations. AFM images for samples shown in Fig. 2g. Scale bars: 1 μm.

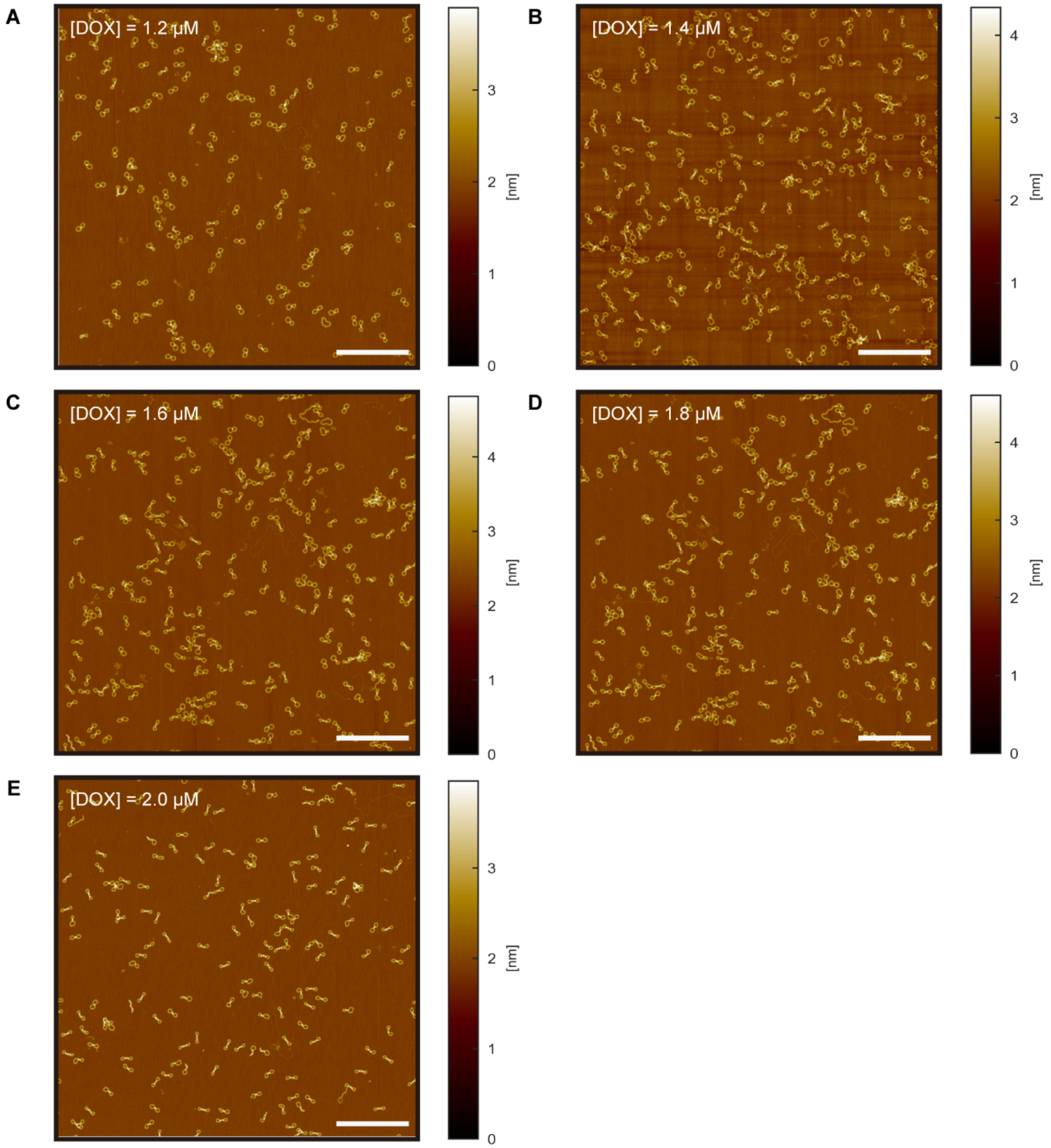

Supplementary Figure 23. Representative AFM images of the 6HB closed ring with respect to DOX concentrations. AFM images for samples shown in Fig. 2g. Scale bars: 1 μm.

Concentration of DOX [μM]	0.20	0.40	0.60	0.80	0.90
Number of samples	526	733	877	792	365
Average	0.0322	0.0191	0.0400	0.3131	0.4980
Standard deviation	0.0078	0.0051	0.0096	0.0166	0.0262

Concentration of DOX [μM]	1.00	1.20	1.40	1.60	1.80
Number of samples	373	694	610	524	484
Average	0.8285	0.9855	0.9934	0.9962	0.9980
Standard deviation	0.0195	0.0045	0.0032	0.0027	0.0020

Concentration of DOX [μM]	2.00
Number of samples	602
Average	1.0000
Standard deviation	0.0000

Supplementary Figure 24. Detailed experimental data on R_{NC} of the 6HB closed rings with respect to DOX concentration. Standard deviation of the R_{NC} was calculated by a bootstrap method with a subset of the given number of samples randomly chosen with replacement and 10,000 repeats of the process.

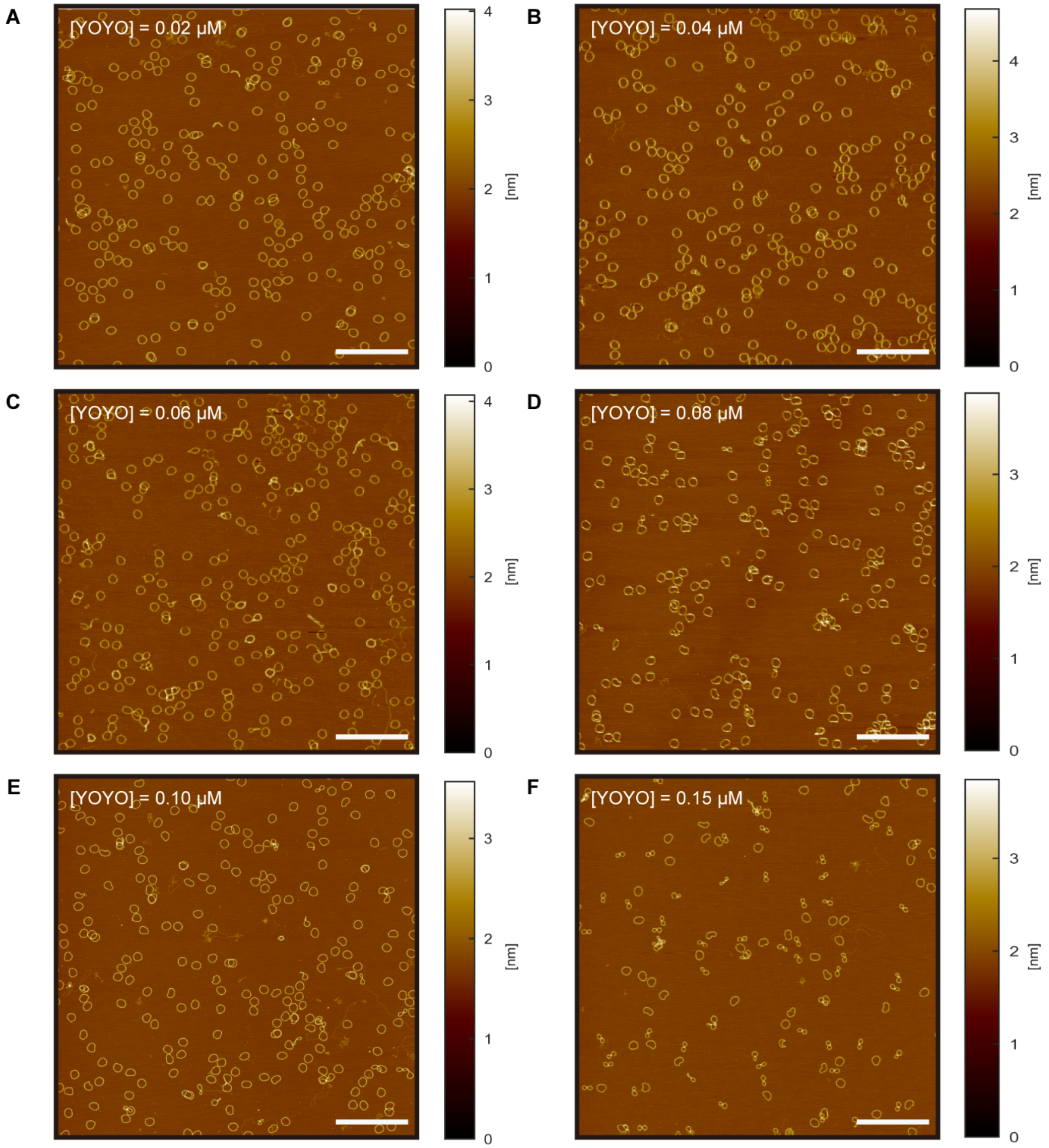

Supplementary Figure 25. Representative AFM images of the 6HB closed ring with respect to YOYO-1 concentrations. AFM images for samples shown in Fig. 2g. Scale bars: 1 μm.

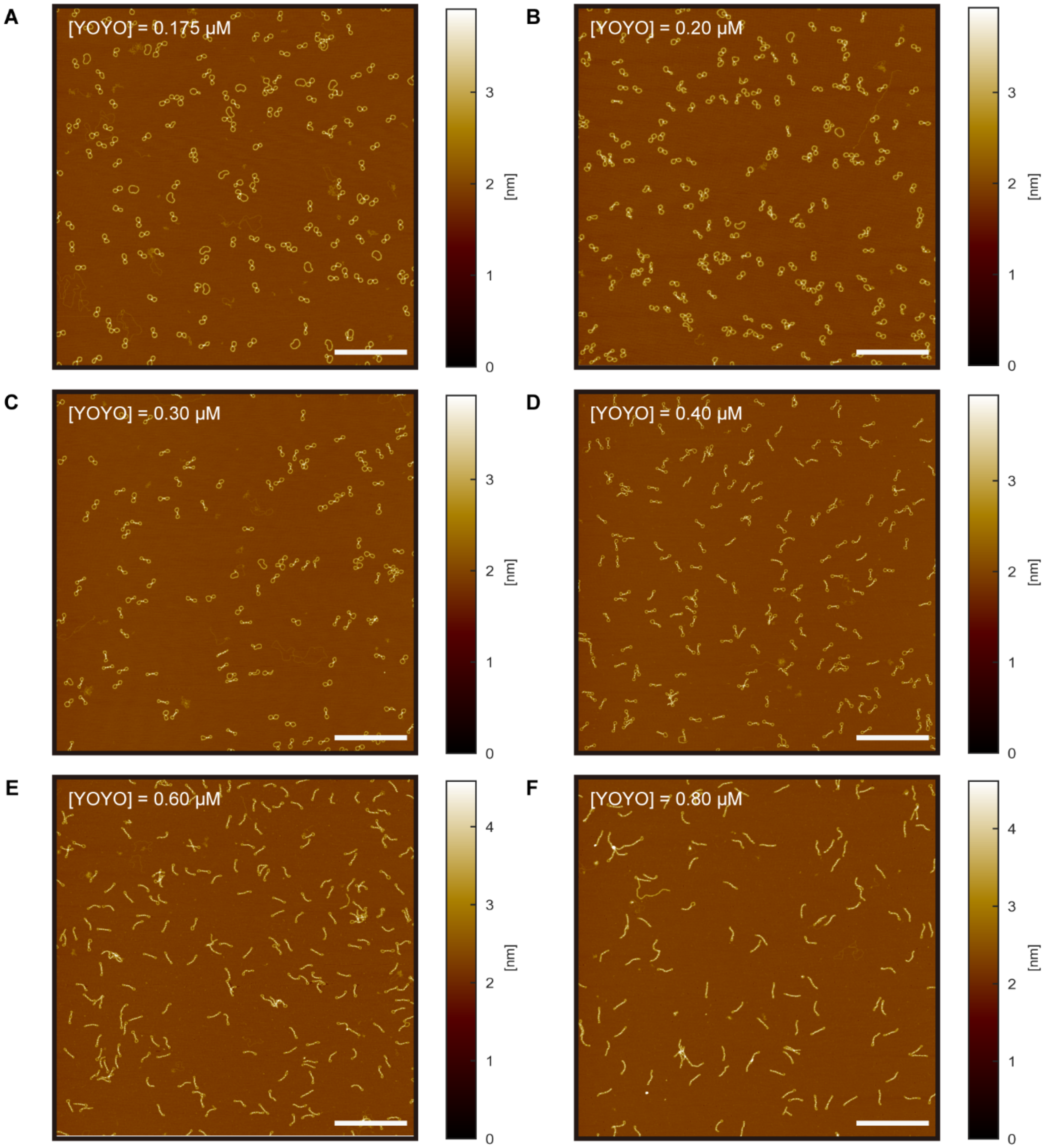

Supplementary Figure 26. Representative AFM images of the 6HB closed ring with respect to YOYO-1 concentrations. AFM images for samples shown in Fig. 2g. Scale bars: 1 μm.

Concentration of YOYO-1 [μM]	0.02	0.04	0.06	0.08	0.10
Number of samples	646	411	536	472	632
Average	0.0139	0.0146	0.0010	0.0277	0.0492
Standard deviation	0.0046	0.0058	0.0000	0.0075	0.0086

Concentration of YOYO-1 [μM]	0.15	0.175	0.20	0.25	0.30
Number of samples	834	510	328	589	416
Average	0.5001	0.9471	0.9817	1.0000	0.9903
Standard deviation	0.0174	0.0098	0.0073	0.0000	0.0048

Concentration of YOYO-1 [μM]	0.40	0.60
Number of samples	492	484
Average	0.9980	1.0000
Standard deviation	0.0020	0.0000

Supplementary Figure 27. Detailed experimental data on R_{NC} of the 6HB closed rings with respect to YOYO-1 concentration. Standard deviation of the R_{NC} was calculated by a bootstrap method with a subset of the given number of samples randomly chosen with replacement and 10,000 repeats of the process.

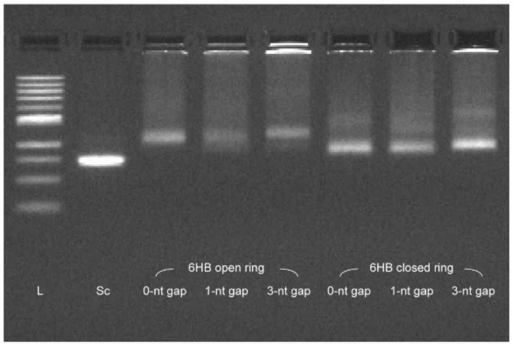

Supplementary Figure 28. Agarose gel electrophoresis results of the purified structures shown in Figure 3b. A clear monomer band was observed in all structures. L: 1kb DNA ladder and Sc: Scaffold strand.

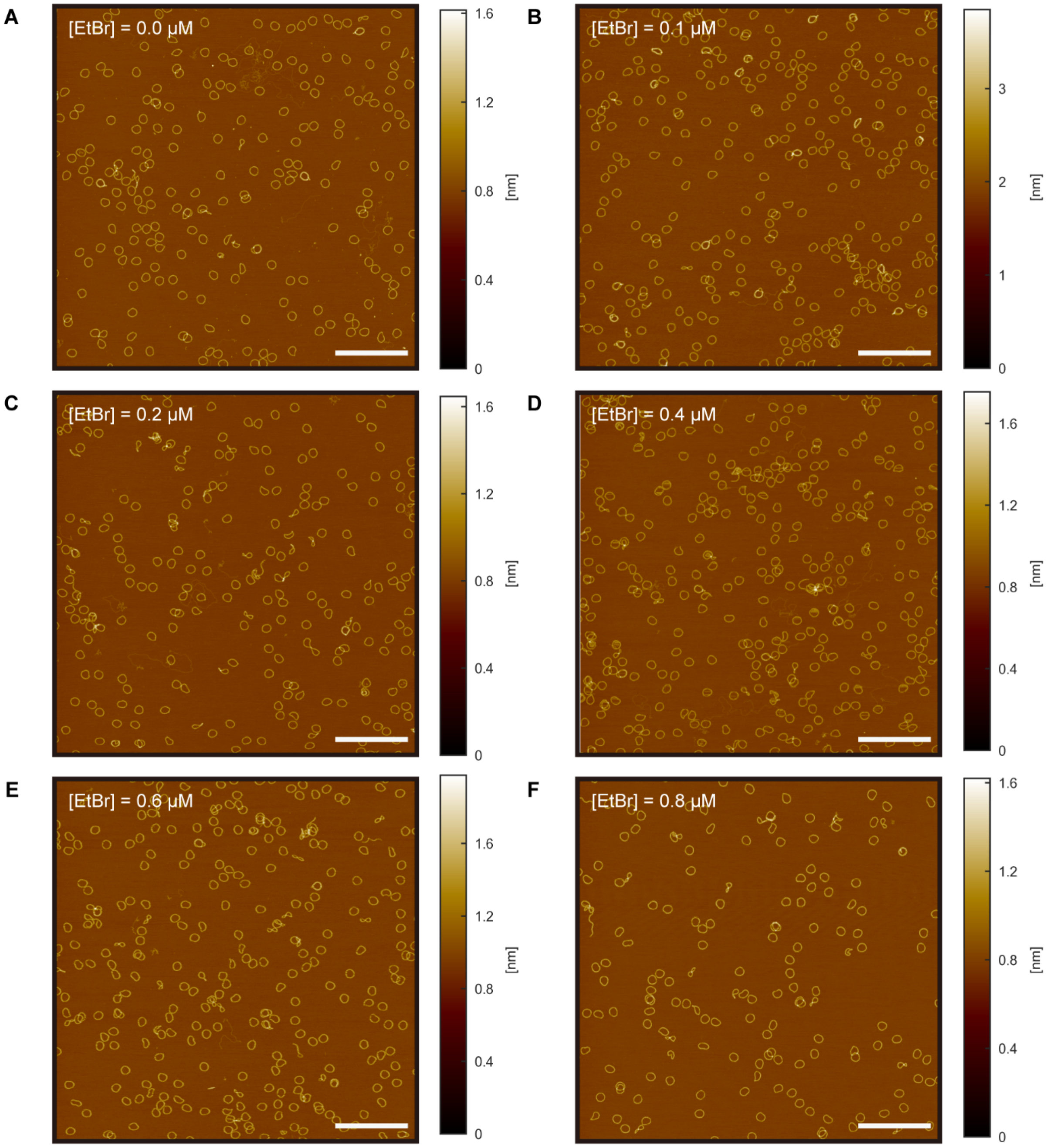

Supplementary Figure 29. Representative AFM images of the 6HB closed ring with 1-nt gap with respect to EtBr concentrations. AFM images for samples shown in Fig. 3b. Scale bars: 1 μm.

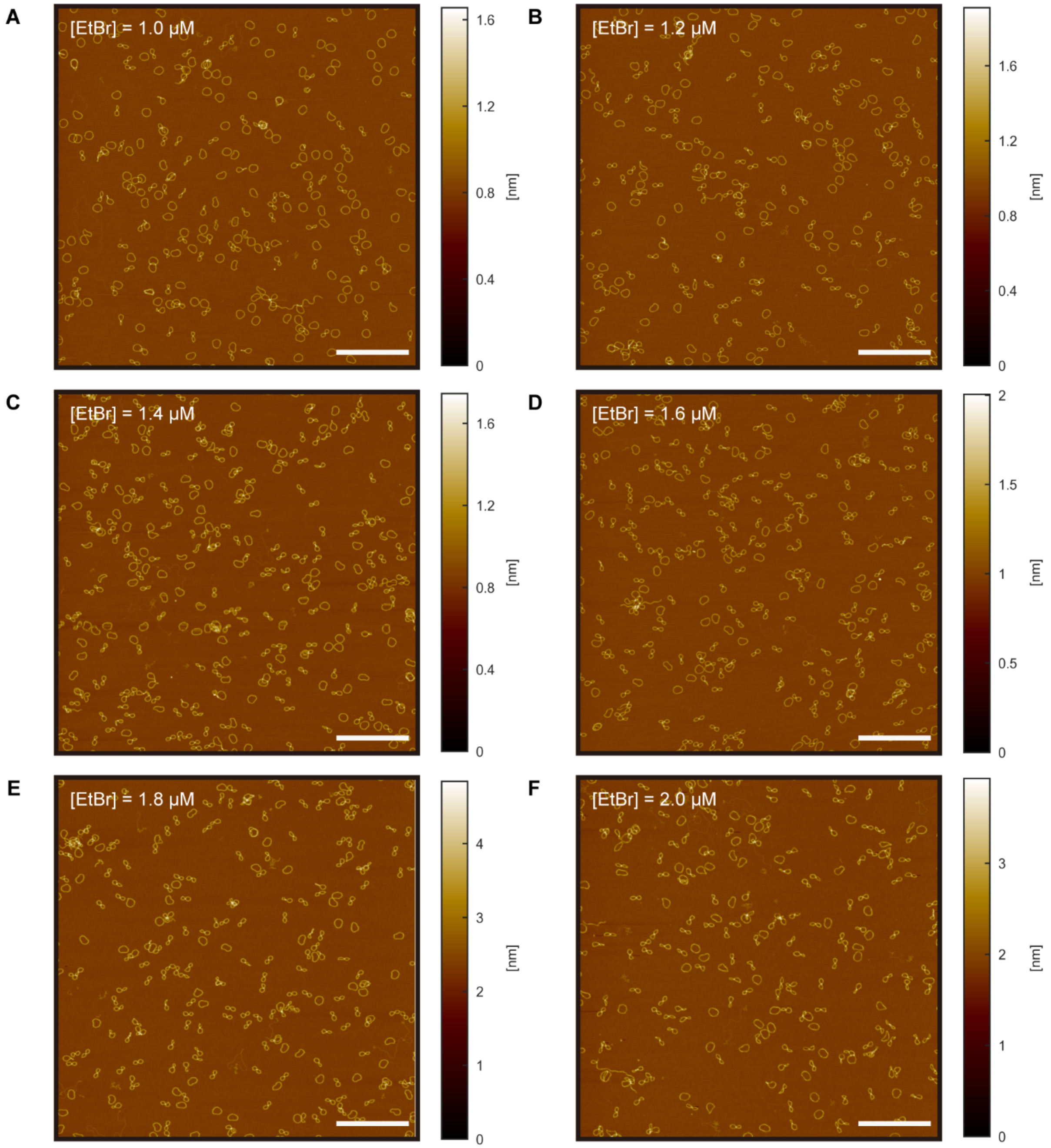

Supplementary Figure 30. Representative AFM images of the 6HB closed ring with 1-nt gap with respect to EtBr concentrations. AFM images for samples shown in Fig. 3b. Scale bars: 1 μm.

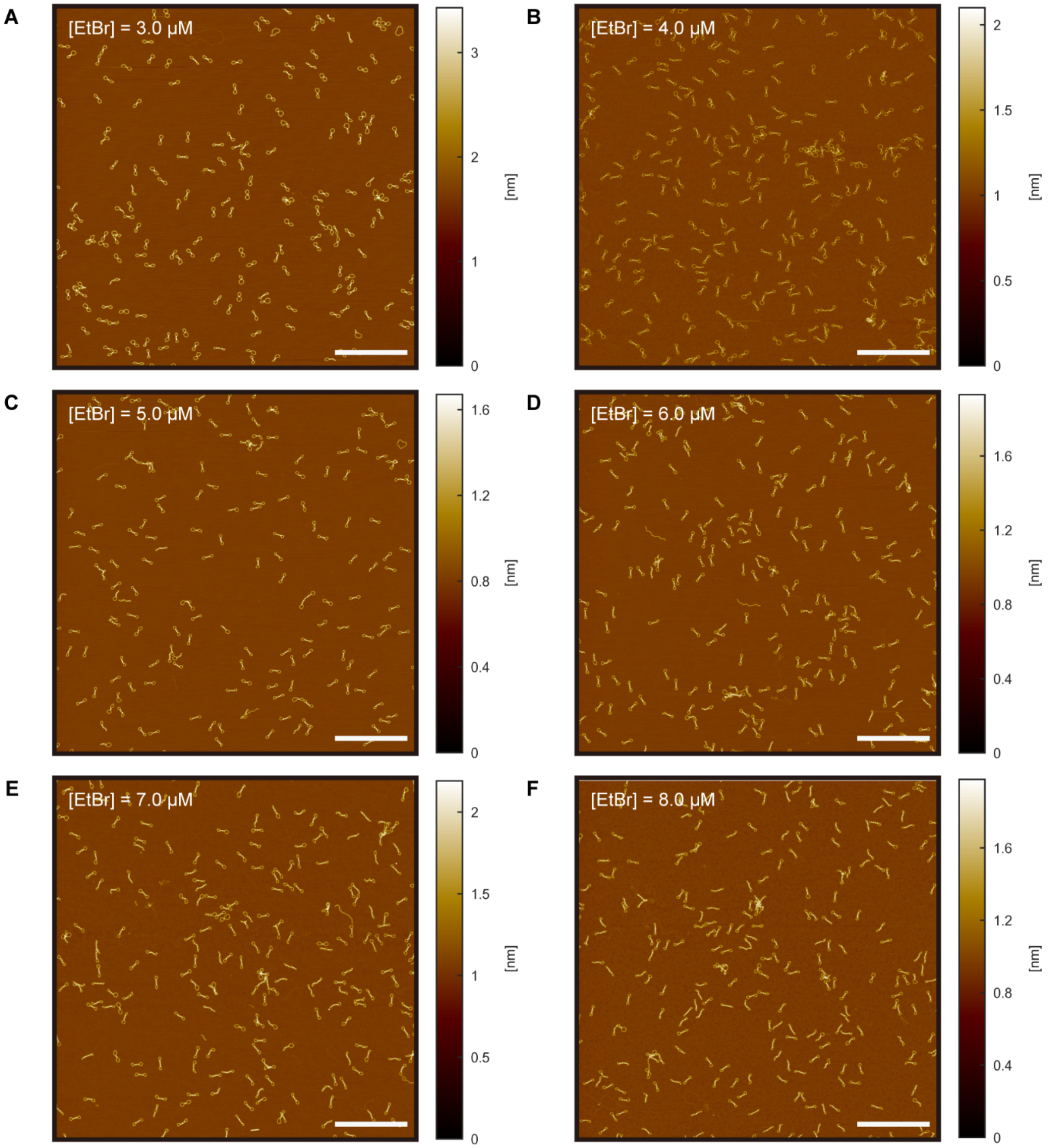

Supplementary Figure 31. Representative AFM images of the 6HB closed ring with 1-nt gap with respect to EtBr concentrations. AFM images for samples shown in Fig. 3b. Scale bars: 1 μm.

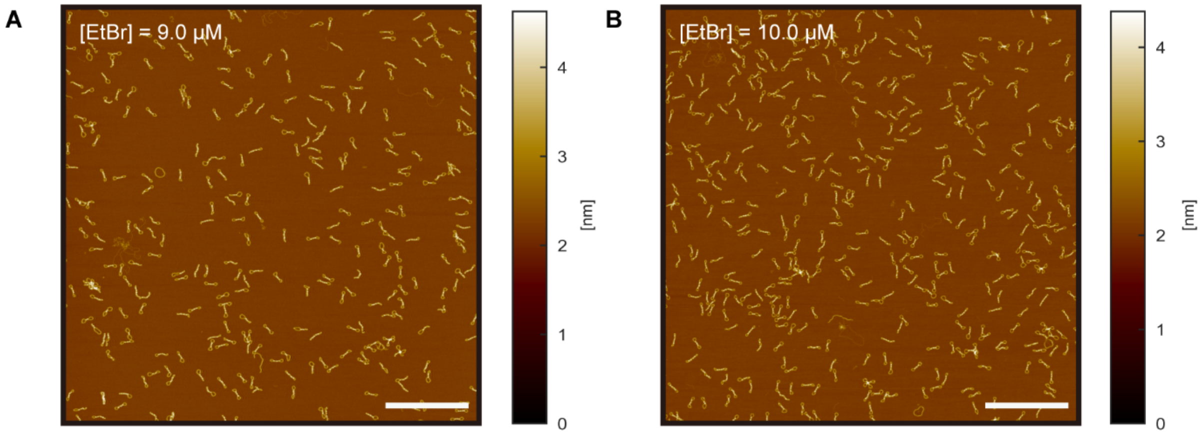

Supplementary Figure 32. Representative AFM images of the 6HB closed ring with 1-nt gap with respect to EtBr concentrations. AFM images for samples shown in Fig. 3b. Scale bars: 1 μm.

Concentration of EtBr [μM]	0.0	0.1	0.2	0.4	0.6
Number of samples	757	561	596	1127	750
Average	0.0066	0.0159	0.0252	0.0160	0.0293
Standard deviation	0.0029	0.0053	0.0064	0.0037	0.0061

Concentration of EtBr [μM]	0.8	1.0	1.2	1.4	1.6
Number of samples	681	770	713	1023	935
Average	0.0395	0.2339	0.4980	0.6237	0.7165
Standard deviation	0.0079	0.0151	0.01188	0.0152	0.0148

Concentration of EtBr [μM]	1.8	2.0	3.0	4.0	5.0
Number of samples	1153	755	789	985	657
Average	0.8013	0.8529	0.9429	0.9898	0.9934
Standard deviation	0.0118	0.0130	0.0083	0.0038	0.0020

Concentration of EtBr [μM]	6.0	7.0	8.0	9.0	10.0
Number of samples	705	1084	927	484	994
Average	0.9982	0.9870	1.0000	0.9979	0.9990
Standard deviation	0.0010	0.0035	0.0000	0.0021	0.0010

Supplementary Figure 33. Detailed experimental data on R_{NC} of the 6HB closed rings with 1-nt gap. Standard deviation of the R_{NC} was calculated by a bootstrap method with a subset of the given number of samples randomly chosen with replacement and 10,000 repeats of the process.

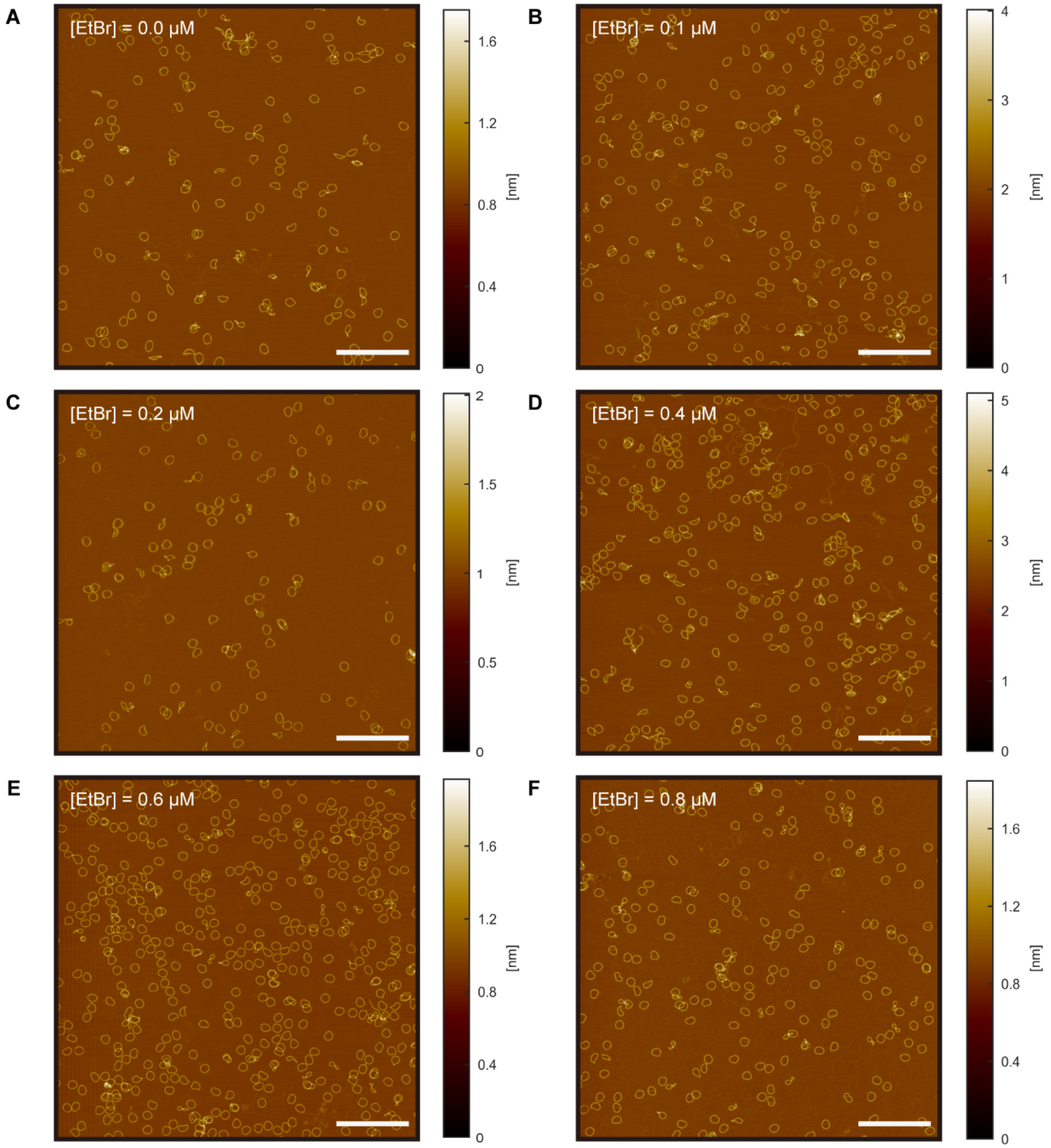

Supplementary Figure 34. Representative AFM images of the 6HB closed ring with 3-nt gap with respect to EtBr concentrations. AFM images for samples shown in Fig. 3b. Scale bars: 1 μm.

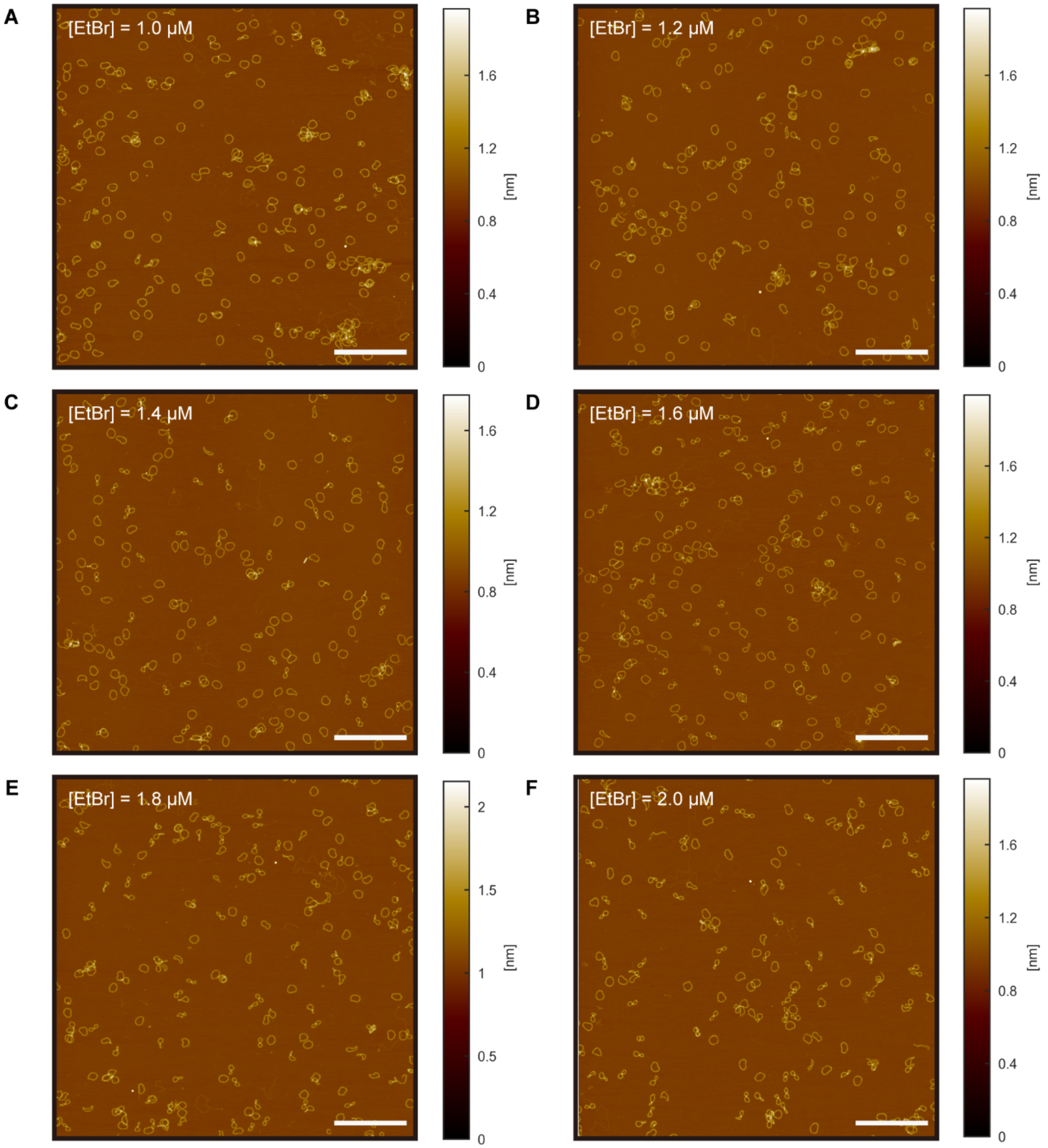

Supplementary Figure 35. Representative AFM images of the 6HB closed ring with 3-nt gap with respect to EtBr concentrations. AFM images for samples shown in Fig. 3b. Scale bars: 1 μm.

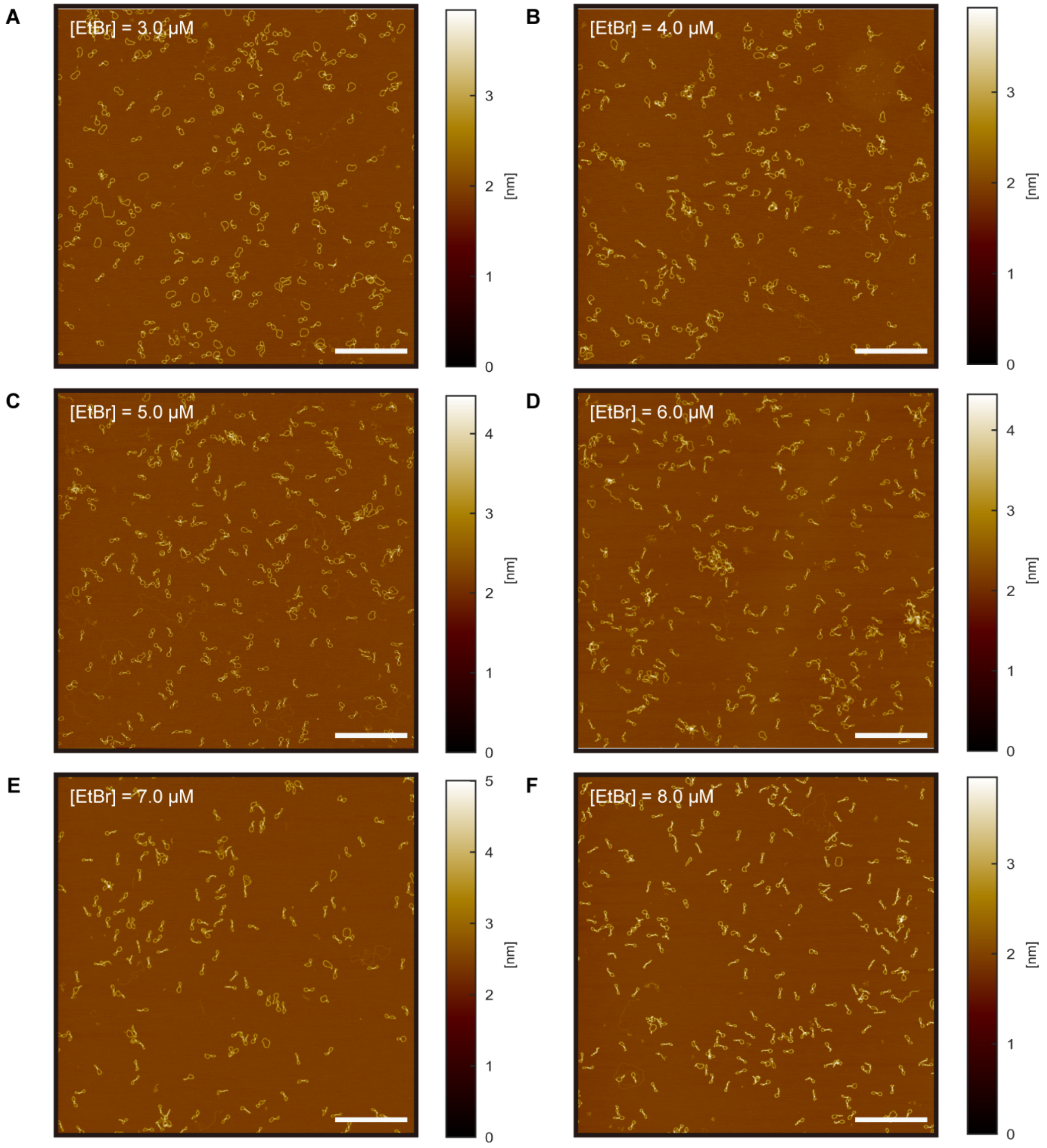

Supplementary Figure 36. Representative AFM images of the 6HB closed ring with 3-nt gap with respect to EtBr concentrations. AFM images for samples shown in Fig. 3b. Scale bars: 1 μm.

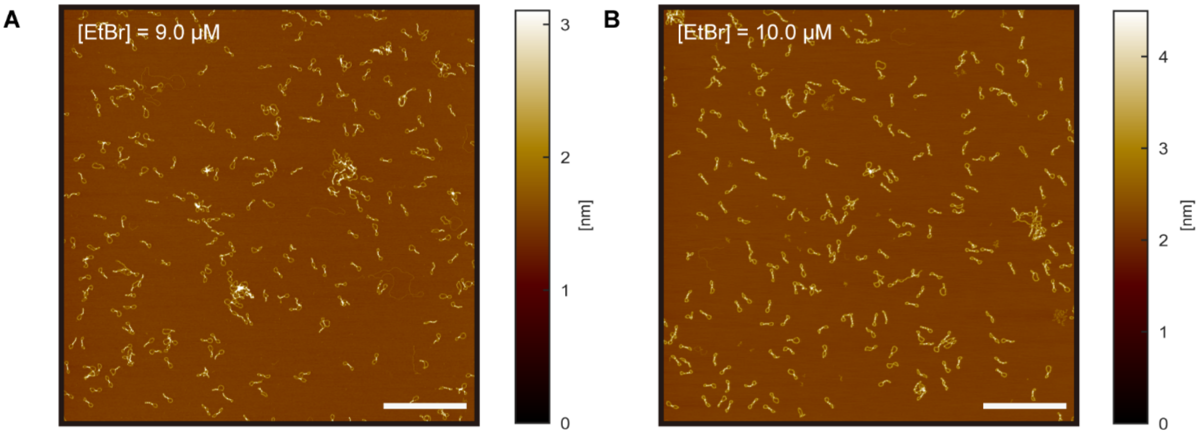

Supplementary Figure 37. Representative AFM images of the 6HB closed rings with 3-nt gap with respect to EtBr concentrations. AFM images for samples shown in Fig. 3b. Scale bars: 1 μm.

Concentration of EtBr [μM]	0.0	0.1	0.2	0.4	0.6
Number of samples	356	548	323	1080	1045
Average	0.0337	0.0161	0.0311	0.0250	0.0325
Standard deviation	0.0096	0.0052	0.0095	0.0048	0.0055

Concentration of EtBr [μM]	0.8	1.0	1.2	1.4	1.6
Number of samples	490	988	563	499	907
Average	0.0471	0.0709	0.1137	0.2464	0.3461
Standard deviation	0.0096	0.0081	0.0133	0.0194	0.0159

Concentration of EtBr [μM]	1.8	2.0	3.0	4.0	5.0
Number of samples	504	576	607	539	674
Average	0.4783	0.5384	0.8255	0.9536	0.9659
Standard deviation	0.0221	0.0205	0.0154	0.0090	0.0070

Concentration of EtBr [μM]	6.0	7.0	8.0	9.0	10.0
Number of samples	649	580	580	323	498
Average	0.9831	0.9724	0.9914	1.0000	0.9940
Standard deviation	0.0050	0.0067	0.0038	0.0000	0.0035

Supplementary Figure 38. Detailed experimental data on R_{NC} of the 6HB closed rings with 3-nt gap. Standard deviation of the R_{NC} was calculated by a bootstrap method with a subset of the given number of samples randomly chosen with replacement and 10,000 repeats of the process.

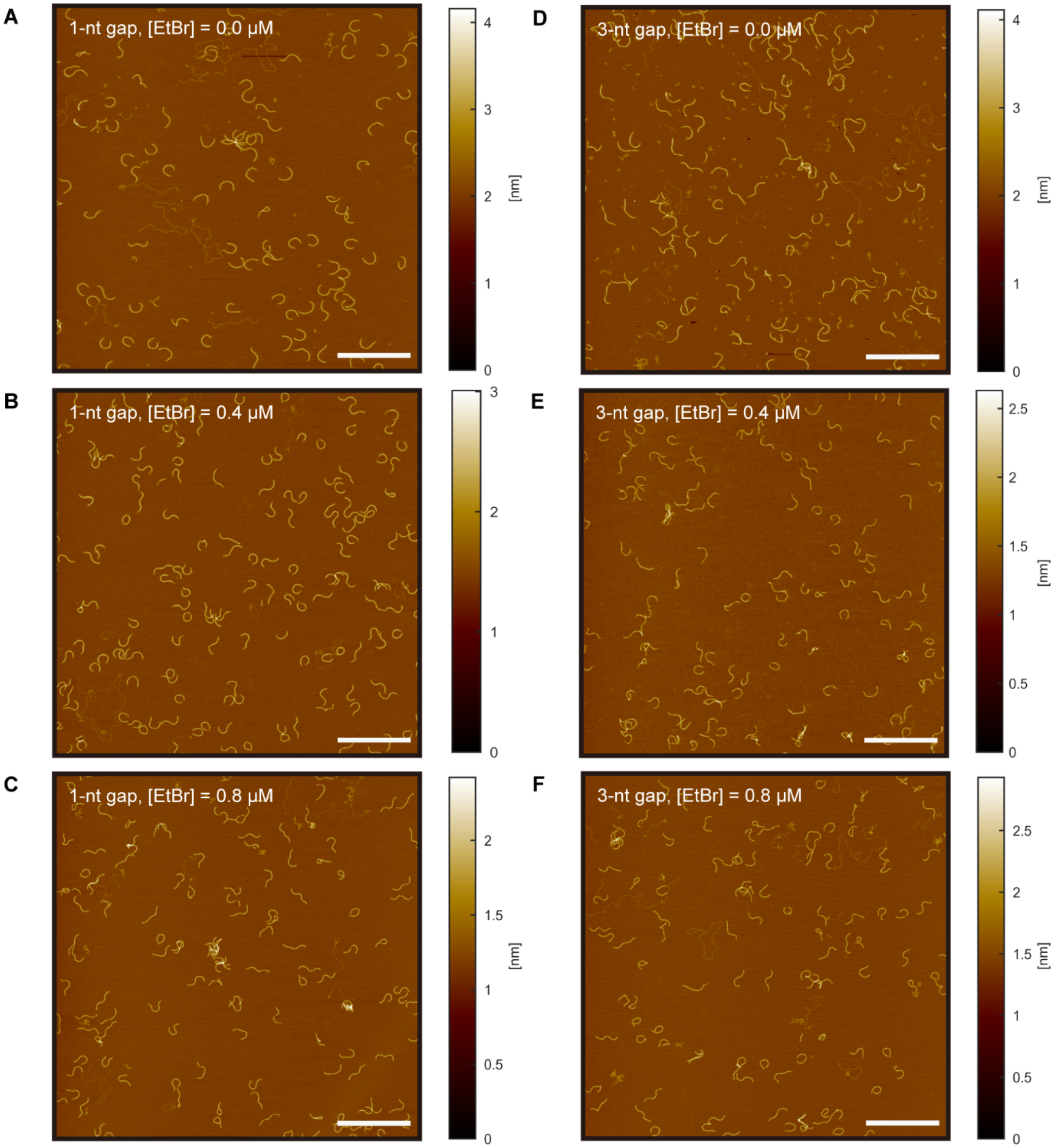

Supplementary Figure 39. Representative AFM images of the 6HB open ring with gap with respect to EtBr concentrations. AFM images for samples shown in Fig. 3d. Scale bars: 1 μm.

A

Concentration of EtBr [μM]	0.0	0.4	0.8
Number of samples	384	358	354
Average	0.2920	0.6701	0.8580
Standard deviation	0.0229	0.0244	0.0220

B

Concentration of EtBr [μM]	0.0	0.4	0.8
Number of samples	423	371	465
Average	0.5458	0.6984	0.7955
Standard deviation	0.0242	0.0237	0.0190

Supplementary Figure 40. Detailed experimental data on R_{NC} of the 6HB open rings with gap. (A) For 1-nt gap and (B) 3-nt gap. Standard deviation of the RNC was calculated by a bootstrap method with a subset of the given number of samples randomly chosen with replacement and 10,000 repeats of the process.

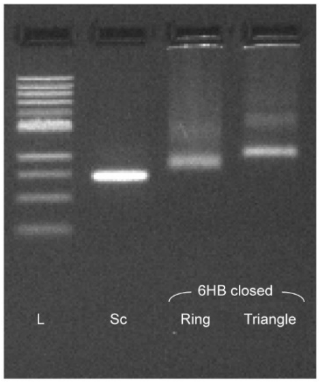

Supplementary Figure 41. Agarose gel electrophoresis results of the structures shown in Figure 4a. A clear monomer band was observed in all structures. L: 1kb DNA ladder and Sc: Scaffold strand.

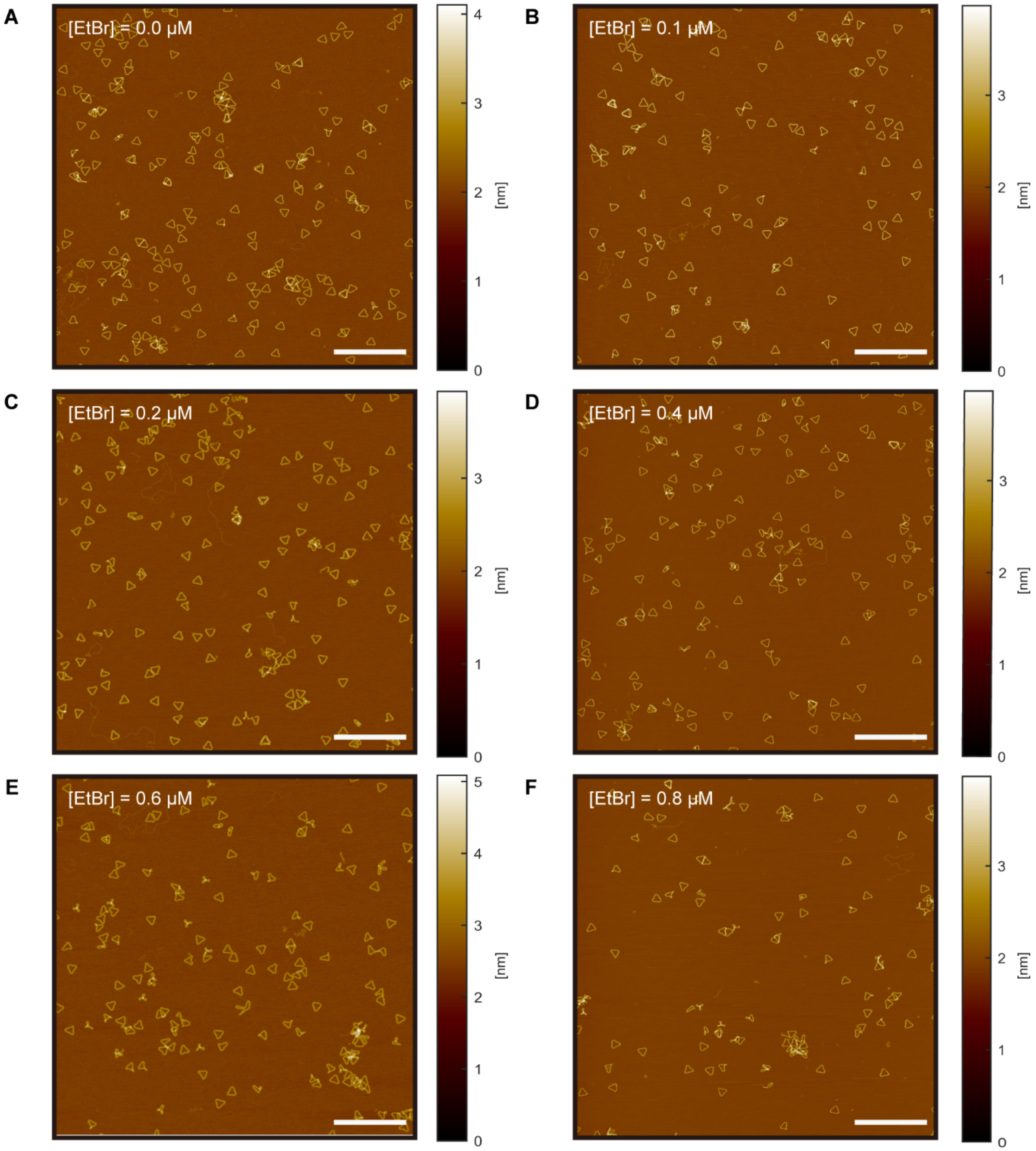

Supplementary Figure 42. Representative AFM images of the 6HB closed triangle with respect to EtBr concentrations. AFM images for samples shown in Fig. 4a. Scale bars: 1 μm.

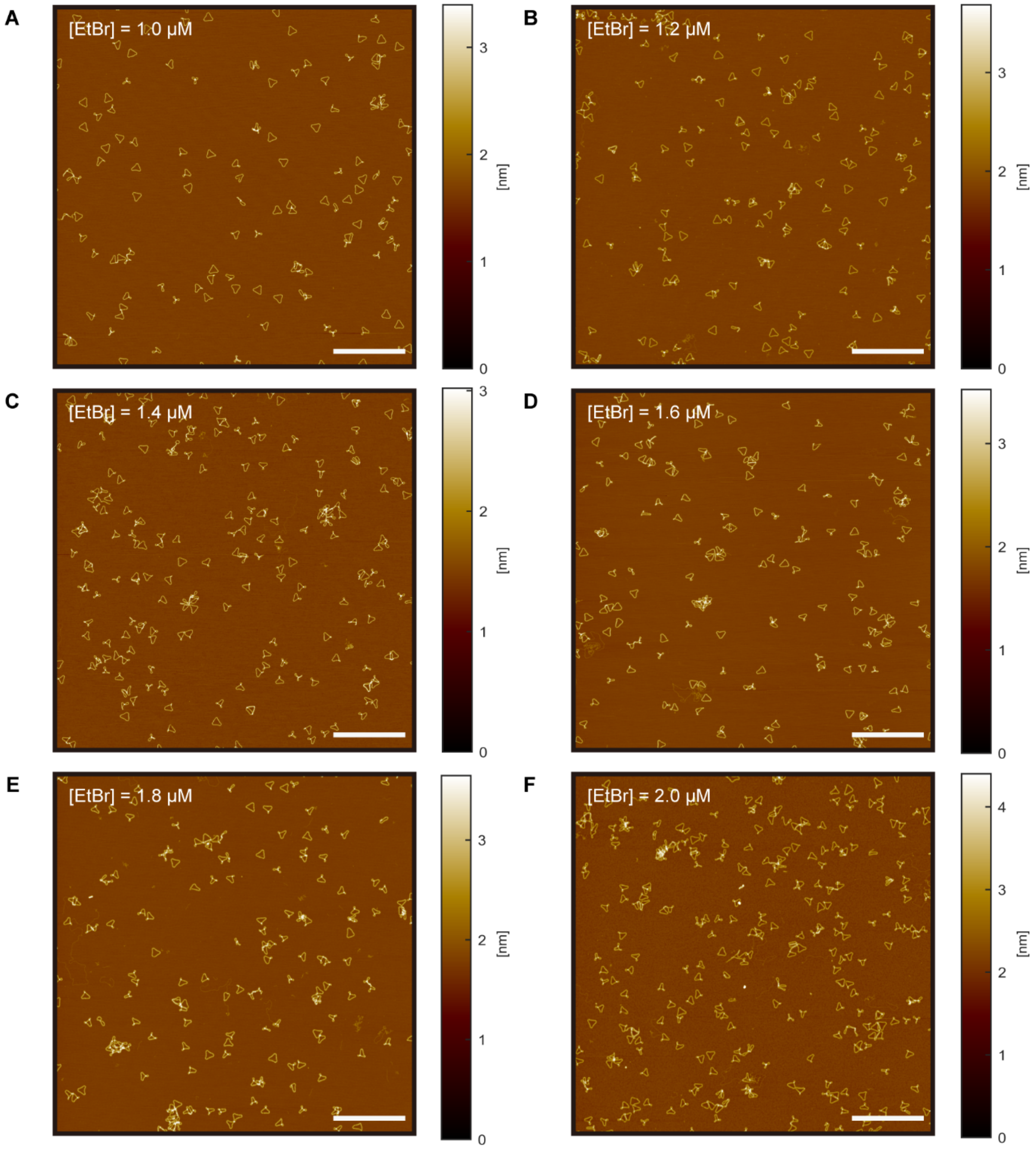

Supplementary Figure 43. Representative AFM images of the 6HB closed triangle with respect to EtBr concentrations. AFM images for samples shown in Fig. 4a. Scale bars: 1 μm.

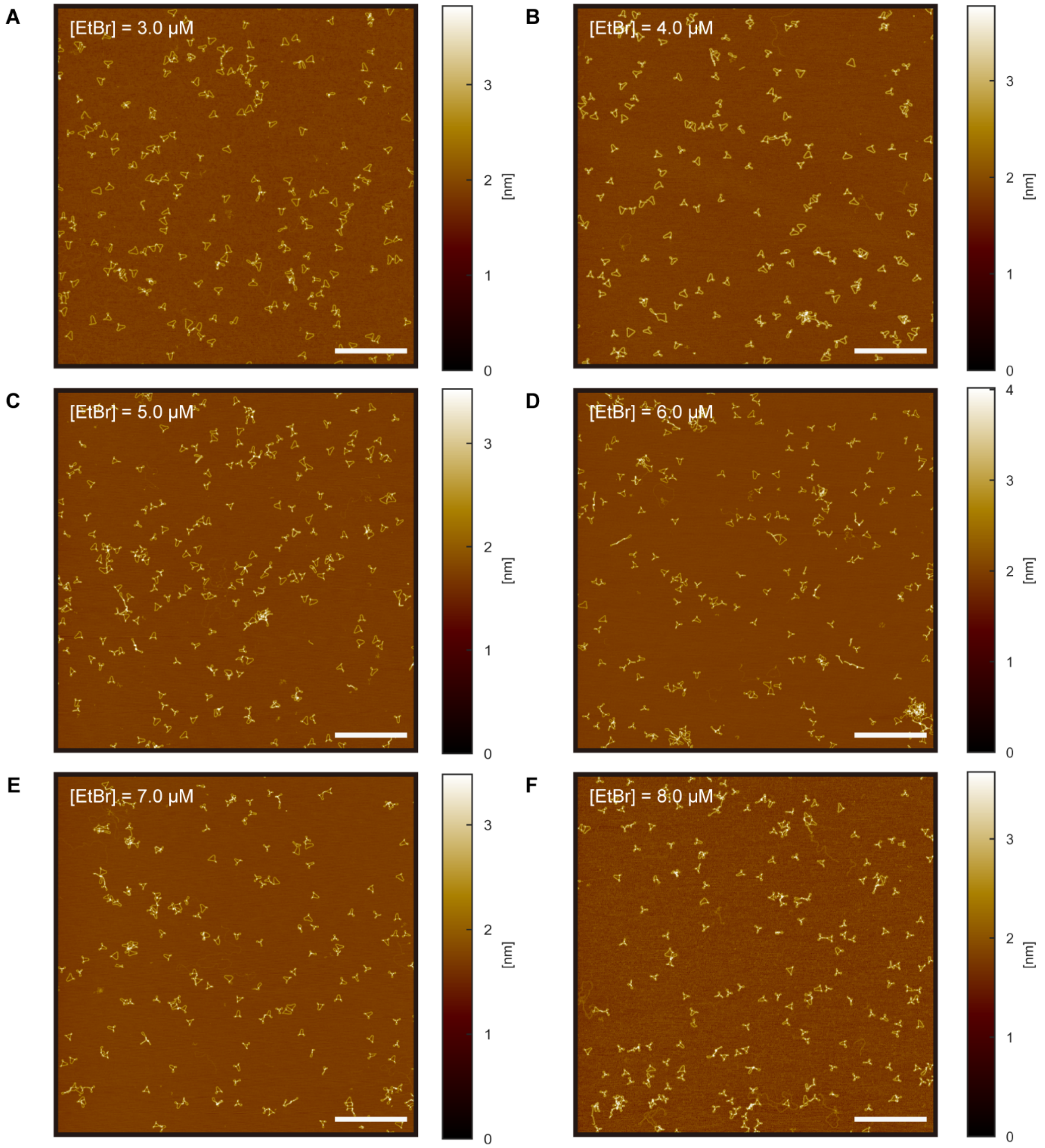

Supplementary Figure 44. Representative AFM images of the 6HB closed triangle with respect to EtBr concentrations. AFM images for samples shown in Fig. 4a. Scale bars: 1 μm.

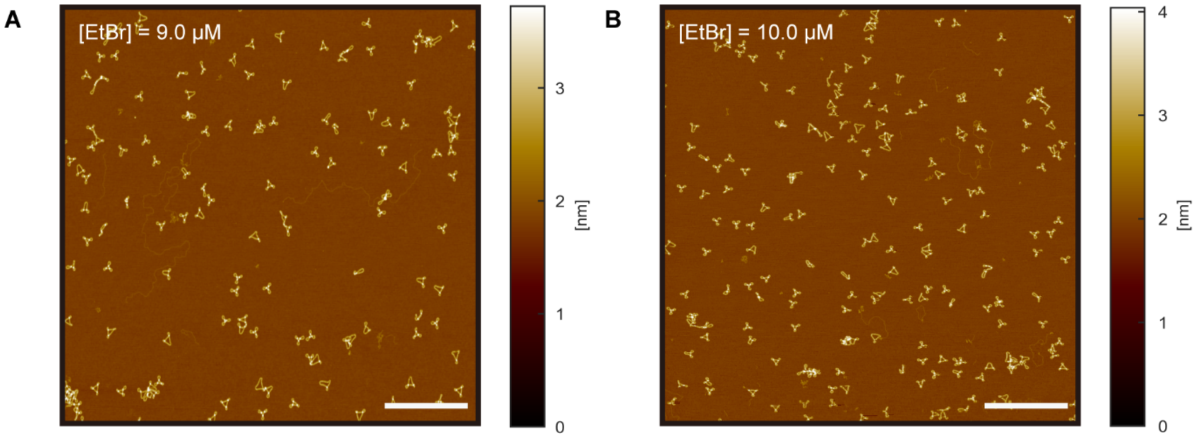

Supplementary Figure 45. Representative AFM images of the 6HB closed triangle with respect to EtBr concentrations. AFM images for samples shown in Fig. 4a. Scale bars: 1 μm.

Concentration of EtBr [μM]	0.0	0.1	0.2	0.4	0.6
Number of samples	458	582	462	541	410
Average	0.0001	0.0500	0.1100	0.1800	0.2949
Standard deviation	0.0158	0.0147	0.0178	0.0175	0.0225

Concentration of EtBr [μM]	0.8	1.0	1.2	1.4	1.6
Number of samples	375	451	369	581	594
Average	0.3356	0.4408	0.5938	0.7537	0.7643
Standard deviation	0.0242	0.0233	0.0254	0.0179	0.0173

Concentration of EtBr [μM]	1.8	2.0	3.0	4.0	5.0
Number of samples	500	478	628	453	530
Average	0.8579	0.8307	0.9539	0.9690	0.9987
Standard deviation	0.0158	0.0173	0.0085	0.0081	0.0046

Concentration of EtBr [μM]	6.0	7.0	8.0	9.0	10.0
Number of samples	425	520	570	438	560
Average	0.9882	0.9981	0.9948	0.9954	0.9982
Standard deviation	0.0052	0.0019	0.0030	0.0030	0.0018

Supplementary Figure 46. Detailed experimental data on R_{NC} of the 6HB closed triangles. Standard deviation of the R_{NC} was calculated by a bootstrap method with a subset of the given number of samples randomly chosen with replacement and 10,000 repeats of the process.

Supplementary Tables: List of staple sequences

Supplementary Table 1. DNA sequence for the 6HB closed ring structure.

6HB closed ring (no gap)		
**Name**	**Sequence (5'→3')**	**Color**
**6HB_connector_001**	ACTCACATTAATAGCTATCATTTG	
**6HB_connector_002**	AATAACCTGTTTTGCGTTGCGCTC	
**6HB_connector_003**	CCTGTTGACCATTAGATACATTTC	
**6HB_connector_004**	CGAACGAGTAGATTTAGTTCGTGC	
**6HB_connector_005**	CGGTTTGCGTTTCATTCCATATAAC	
**6HB_connector_006**	GTACGGTGTCTGGAAGTATTGGGCG	
**6HB_LE_001**	AAGCCTGCCAGTGATCTATCAGGGCCAACGCGCGGGGTTTTT	
**6HB_LE_002**	ACTGCCCGCTTTGCTTTCCTCGTTAGAACGAGCACGTATAACGTCCAGT	
**6HB_LE_003**	CGGGAAACAGCTGCATTAATGAATCGGCGATGGCC	
**6HB_LE_004**	CTTTTCAGGGTGCCTAATGAGTGAGCTACCAGGGTGGAGAGG	
**6HB_RE_001**	GGGCGCGAACTAAAAGTTGATTCAACGA	
**6HB_RE_002**	GCAAAAACAACATTATTACAGTAAAACGAAGGTGG	
**6HB_RE_003**	AACAGTTCAGAACCAATTCTG	
**6HB_RE_004**	GAATGACTGTTTTAAATATGCAGCTGAAAACTAACGGTGGTC	
**6HB_SC_001**	TGCCAAGTAGATAAAAATATCTTTAGGATTAATGCAATATTT	
**6HB_SC_002**	AATTAACCGTTGTAGCCTGCAGGTCGACTCTAGAGTCACGCA	
**6HB_SC_003**	TTGAATGGCAAAGGAAGGGAAGAAAGCGCTGTCCAGATCCCC	
**6HB_SC_004**	AGCCGGCTTATAAAGTGGTTCCGAAATCGGAATAGATTAGAG	
**6HB_SC_005**	GCGAGTATTAGTCTGCACTAACAACTCAAAATCCCGAACGTG	
**6HB_SC_006**	CCGTCAACTTGCATGCAATACTTCTTTGCACAGACGCGAACT	
**6HB_SC_007**	ATTCAGGAGCGGAAAATACCAAGTTACATACCTGAAGGTTGG	
**6HB_SC_008**	AAATGCTACTGTTGGGAAGGGAACAAAGGCAGAGGCGGCAAA	
**6HB_SC_009**	GTTATATAACTAACGCTCAATCGTCTGAAAATACCTGCGGGC	
**6HB_SC_010**	TGAGAGCCAGCAAATTATTCATTTCAATAAATCGCAAACCAC	
**6HB_SC_011**	TGAAAAATTATCATTTTGCGGCGATCGGTACATTTTGTATGT	
**6HB_SC_012**	CAGAAGGCTGCGCAGATGCAAATCCAATCCGGCTTGCAAAAG	
**6HB_SC_013**	GGTCACGACGAGCGATTTTGCACCCAGCTGTAGAAAATATCC	
**6HB_SC_014**	CCGGAATCATAATTAGATGGGCGCATCGTAACCGTATAAACA	
**6HB_SC_015**	CATCCTAATAATCGTCGCTATTAATTAAAATAAGAGCATCTG	
**6HB_SC_016**	GAATAACAGAAATATATCAAAATTATTTGCCTGAATCTTACC	
**6HB_SC_017**	CTGTATTACGAGCATACAATTTTATCACGTAAAACCTTGCTT	
**6HB_SC_018**	AACGCTATTGGTGTACTAGAAAAAGCCTGAAAAATACCAATC	
**6HB_SC_019**	GCTCATTGGAAGCGAACTGGCATGATTATAGAAAAACCAATG	
**6HB_SC_020**	ACCGTAACAATAGGAACGCCAGAGAGAATTATTACGCAGCAA	
**6HB_SC_021**	AAACCATCGATACAGTAATAAGAGAATAAGAGGCATAATTCG	

**6HB_SC_022**	ACCGCGCCCAATAGTATGTTAGCAAACGAGACTCCTAACATA	
**6HB_SC_023**	GCAAATCTAGCAGCCTTTACATCAAAAATTTTCGAGCGCAGC	
**6HB_SC_024**	AAAACAGTTTTAACTCAGTAGCGACAGAAAACGTCTACATAC	
**6HB_SC_025**	ACGGTAACCAGTACGCAAGCCCAATAGGCTATTTCCGTATAA	
**6HB_SC_026**	CCGCCTCCCTCAGAACTAGCATGTCAATCATATGTACCGGAA	
**6HB_SC_027**	ACAGTTAATGAGGGAGGGAAGGTAAATAAGCCACCACCCCGG	
**6HB_SC_028**	GGGCGACGCAATAGAATAAGAGCAAGAAACGTAACACTGAGT	
**6HB_SC_029**	CGATTGCCCCCTGCAACCCATGTACCAATGAAATAATTCAAC	
**6HB_SC_030**	TTCGTCATCGTAAAGCCGCCACCCTCAGCAGTGCCGGAACCT	
**6HB_SC_031**	GGCCGGACGGAGTGTTTTGCGGGATCGTAGGGTAGCATAAGG	
**6HB_SC_032**	TTTGAAAAAATCACCATCAATAACAACTAGCAGCGAAATAGC	
**6HB_SC_033**	GAACCGAACTGACCTCATTAAAGCCAGATCACAAATCAACCG	
**6HB_SC_034**	GTTGATATAAGTAGACAGCATCGGAACGCACCCTCTTCAACA	
**6HB_SC_035**	CCGGAATATGGGATTTTGCTAATGATATCAAATAAATCCAAC	
**6HB_SC_036**	GTTTCAGGACAGTCGAGGACAGATGAACGGTCAATCAACGGC	
**6HB_SC_037**	CGGTTGTCCAACAGGCGTTTTAATTCGAGAGAGGCAGACGAC	
**6HB_SC_038**	CGAGAAACACCAGAACATTATGACCCTGTAATACTGCCCTGA	
**6HB_SC_039**	GATAAAAACTCATCTTTGACCCCCAGCGAAGGCTTTTTGCGG	
**6HB_SC_040**	AAAGAATTTCTTAATTATCAGCTTGCTTTCACCAGACCGGAA	
**6HB_SC_041**	AACACCAAAATAGCGCTTCAAAGCGAGAGGTGAATACACTAA	
**6HB_SC_042**	GCAAACTACCAAAAACGAGTAGTAAATTGTTTACCTTTTGCA	
**6HB_B1_001**	GAGCCGGTGATTGCCGTCAAAGGGCGATGA	
**6HB_B1_002**	TCACAATGCCCTGATTAAAGAACGTGGTTT	
**6HB_B1_003**	CTGTGTGAGCGGTCTTTGGAACAAGAGTAA	
**6HB_B1_004**	AATCATGCCCAGCAAGGGTTGAGTGTTTAA	
**6HB_B1_005**	GGGTACCTTTGATGTCAAAAGAATAGCAGC	
**6HB_B1_006**	AATCAAGACTCCAACCTTCACCGCCTGTCCA	
**6HB_B1_007**	GGTGCCGTCCACTAGAGAGTTGCAGCAAAAT	
**6HB_B1_008**	GGAACCCGTTCCAGCACGCTGGTTTGCGTCA	
**6HB_B1_009**	GATTTAGCCGAGATGGCGAAAATCCTGGAGC	
**6HB_B1_010**	CACTACGAAAACCGGACGGGCAACAGCAAGC	
**6HB_B1_011**	ATAGGGAGCTAAACAGGAGCGCGTATCACCCA	
**6HB_B1_012**	CACTAAAGGGATTTTAGAGCGCTTAGGGTCGA	
**6HB_B1_013**	TGTGGTACGCCAGAATCCGCGCGTACTAAATC	
**6HB_B1_014**	TAGTGTTTTTATAATCAGTGGCAAGAGCCCCC	
**6HB_B1_015**	TCGACCGAGTAAAAGAGTAAAGGAGCGGGGAA	
**6HB_B1_016**	ACCACTATGGTTGCTTTGATCAGAGCAAGTGTA	
**6HB_B1_017**	TTTGATGCGCCGCTACAGGGGCCGATAACATAC	
**6HB_B1_018**	AGCAACCACCACACCCGCCCAGGAACTATCCGC	
**6HB_B1_019**	AGGGTGTAGCGGTCACGCTTGAGAAGCTGTTTC	
**6HB_B1_020**	TTGACGGGCGCTAGGGCGCTGAGGCCAATTCGT	

**6HB_B2_001**	GATAGCCTTATCTATACATTTGAGGATAAAA	
**6HB_B2_002**	ACGTTGTTTAGAAGGAATTGAGGAAGGCTA	
**6HB_B2_003**	GCCAGGGAAACAATGGCAAATCAACAGTAC	
**6HB_B2_004**	AAGGCGATTAAATCCAATCAATATCTGAAG	
**6HB_B2_005**	GGCGAAATTATTAAAACCTCAAATATCGGT	
**6HB_B2_006**	CTCTTCGAGTAACATCTAAAGCATCACCAA	
**6HB_B2_007**	TTAAAAATTGAAAGTATTAGACTTTACTTTT	
**6HB_B2_008**	CCAGCAGGTCAGTTTCGACAACTCGTATTAA	
**6HB_B2_009**	GTGAGGCAAACCCTCTTTGCCCGAACGGGGG	
**6HB_B2_010**	CCGCCTGCTTGCTGTTTTAAAAGTTTGCTAT	
**6HB_B2_011**	CGAATAACATCACTTGCCCTGAAAGATCGCCA	
**6HB_B2_012**	CCCAAGAACTCAAACTATACAGAGACGAACCA	
**6HB_B2_013**	GTTGCTGGTAATATCCAGAATAAAAAACAGAG	
**6HB_B2_014**	GATTTACCGCCAGCCATTATTCACCATTAACA	
**6HB_B2_015**	TACGAAAAACGCTCATGGAATGGATGCCACGC	
**6HB_B2_016**	AAACCGTAAGAATACGTGGATTAGTACGGCCAG	
**6HB_B2_017**	CGAATAGAACCCTTCTGACTGAGTAGAGTCACG	
**6HB_B2_018**	ATAAGGGACATTCTGGCCACGGCCTTGGGTAAC	
**6HB_B2_019**	CAGTAGTCACACGACCAGTAACAATAGTGCTGC	
**6HB_B2_020**	CAGTTATTTACATTGGCAGGCAACAGGCCAGCT	
**6HB_B3_001**	AAGATGATGCTTTGTTATCATCATATTAAGC	
**6HB_B3_002**	CCAGGCACCTGATTGGATTCGCCTGATTGA	
**6HB_B3_003**	CACCGCTAATTCATACATCGGGAGAAAAAA	
**6HB_B3_004**	GCACTCCATTGTTTATACAGTAACAGTTTT	
**6HB_B3_005**	AGTATCGGAATAATAGGTTTAACGTCATAA	
**6HB_B3_006**	CCAGTTTCCTACCAAAGAAATTGCGTACAA	
**6HB_B3_007**	AGAAAACCAATAACATCAGATGATGGCTCTG	
**6HB_B3_008**	TTAACAAACCTTTTCAATATAATCCTGAGCC	
**6HB_B3_009**	CCTTTTTGATGAATGGATTATACTTCTGCCT	
**6HB_B3_010**	CATAAATGATTTTCGGAAGGGTTAGAAGAGG	
**6HB_B3_011**	GCCCAAAGAACGCGAGAATGAGAGAAACATCA	
**6HB_B3_012**	GTGTCAAATATATTTTAGGAATTTAATTACAT	
**6HB_B3_013**	AGCCATCTTCTGACCTAAGACGCTGTGAATTA	
**6HB_B3_014**	CAGGGTTTGAAATACCGAACATAGCAACAGTA	
**6HB_B3_015**	GGAATAAATAAGGCGTTATTTTCCCTGTGAGT	
**6HB_B3_016**	AACACTACCTTTTTAACCTCGCAAGAATTCGCC	
**6HB_B3_017**	ATTATCAAAATCATAGGTCAACTTTTCCGGAAA	
**6HB_B3_018**	CATTAGAAGAGTCAATAGTTTAATTTTTTCCGG	
**6HB_B3_019**	TGGAGATAGCTTAGATTAAATTTAATGAAGATC	
**6HB_B3_020**	TATATTAGAATCCTTGAAACCGTGTGCGACGAC	
**6HB_B4_001**	AATAATCAGTTGCTTCTTTCCAGAGCCACCG	

**6HB_B4_002**	CGGATTGTAATTTGTTAAATCAAGATTGGC	
**6HB_B4_003**	ATTCTCCAAACAGCTTGCGGGAGGTTTCAA	
**6HB_B4_004**	GAGCGAGCCAATCCGTTTTAGCGAACCGTA	
**6HB_B4_005**	GCTTTCATTTTTTGGTATTCTAAGAACAGC	
**6HB_B4_006**	CGTCTGGATGAAAAAGATATAGAAGGCTCG	
**6HB_B4_007**	ATCATTCTGAAGCCCCAGTTACAAAATGTGG	
**6HB_B4_008**	AAACCAATCCCGACCATATTATTTATCTAAC	
**6HB_B4_009**	GAGAACAGCGAGGCAAATAAGAAACGATCAA	
**6HB_B4_010**	ATTTTCATTATCCGTTTAACGTCAAAACCTT	
**6HB_B4_011**	TAAATCATATGCGTTATAACAATAGTTTCCTT	
**6HB_B4_012**	GAATTACCAGTATAAAGCTGCAGAAGGGTATT	
**6HB_B4_013**	AACCAACAGTAGGGCTTAAATAAACACTCATC	
**6HB_B4_014**	CATATCGCCATATTTAACGTAATTCCGTTTTT	
**6HB_B4_015**	CCTACATGTAATTTAGGCTAAAGTAAATCATT	
**6HB_B4_016**	TGTCATAAGTCCTGAACAAGTTTAGTTGGGATA	
**6HB_B4_017**	GAACCGCGCCTGTTTATCACAAATTCCAAACGG	
**6HB_B4_018**	CCGCAACATGTTCAGCTAACAACGCTCCGTCGG	
**6HB_B4_019**	AAGCTGTCCAGACGACGACATTGAGATAAATGT	
**6HB_B4_020**	TAGGCCGACAAAAGGTAAAAACGCCAGTAGCCA	
**6HB_B5_001**	CATTAAAAATTAACAATAACGGAATACTGG	
**6HB_B5_002**	TTAATATCAAAGTCGGAAACCGAGGAACAA	
**6HB_B5_003**	CAAATATGCGCTAAGCCGAACAAAGTTTTA	
**6HB_B5_004**	AAAACAGACCCACATTAAGAAAAGTAAAAA	
**6HB_B5_005**	TTGATAAGCCCAATCTATCTTACCGAACGC	
**6HB_B5_006**	ATAAAGGCCAAAAGCATTAGACGGGAGTTTT	
**6HB_B5_007**	AGAAACGACGCAATTGAACACCCTGAATTTG	
**6HB_B5_008**	AATAAGTACCAGAAAGAGGGTAATTGATTAA	
**6HB_B5_009**	TCAATAGGCAGATATATCAGAGAGATAGAAG	
**6HB_B5_010**	TTACCAGGCCCTTTAGAATTGAGTTAATCAG	
**6HB_B5_011**	TGTTTGCCTTTAGCGTCACCATTACATATAAA	
**6HB_B5_012**	TTAGCGCGTTTTCATCGGGCCAGCAACCACGG	
**6HB_B5_013**	ATTGGTCATAGCCCCCTTCTTGAGCGTCACAA	
**6HB_B5_014**	ATTTTTGCCATCTTTTCAGGTGAATATATGGT	
**6HB_B5_015**	AAAAATCACCGGAACCAGTTGACGGGACAAAA	
**6HB_B5_016**	CAACCATTAGCAAGGCCGGATCAAGTTAAATCA	
**6HB_B5_017**	AGACAAATCACCAGTAGCAGACTGTAAAATTCG	
**6HB_B5_018**	TTTTCATTTGGGAATTAGACATTTTCGTAAACG	
**6HB_B5_019**	ATTCTATCACCGTCACCGAATTAGCGGTATAAG	
**6HB_B5_020**	CAAAAAATTATTCATTAAATAATCAAAGCCCCA	
**6HB_B6_001**	AGCAAACTGTAGCACACCCTCATTTTCTGA	
**6HB_B6_002**	GGTCATTCCTCATAACCGCCACCCTCAGGC	

**6HB_B6_003**	AGAGATCTCTAAAGTCAGAACCGCCACGGA	
**6HB_B6_004**	CGGAGAGCCAGACGGAGGTTTAGTACCTGC	
**6HB_B6_005**	TTCTAGCTTTCTGTAGGTGTATCACCGGTG	
**6HB_B6_006**	ATTATTCAGGGATAAAACTACAACGCCAAGA	
**6HB_B6_007**	ATTAAGAGAGCCACTTCCACAGACAGCGCCT	
**6HB_B6_008**	AAGAGAACCTCAGAGTTAGCGTAACGATACA	
**6HB_B6_009**	GGGGTTTGCCACCCTTTTGTCGTCTTTGGTA	
**6HB_B6_010**	CGGATAATACTCAGTTAGTAAATGAATTGAT	
**6HB_B6_011**	GAAACCCTCAGAGCCACCACGGGGTTGAAAGT	
**6HB_B6_012**	GAGGAGCCGCCACCAGAAAGTGTACACTCCTC	
**6HB_B6_013**	AAGCAGAGCCGCCGCCAGACATGGCGATTAGC	
**6HB_B6_014**	GCTAGGAGGTTGAGGCAGTTACCGTTACCAGG	
**6HB_B6_015**	AAAGATTGGCCTTGATATATGGAAACGAGAGG	
**6HB_B6_016**	AACACAGTGCCTTGAGTAAAACCGCCTCGATGA	
**6HB_B6_017**	TGAGTGGTAATAAGTTTTAACCCTCAAGTCTGG	
**6HB_B6_018**	TTAGTTTTGATGATACAGGCCACCACGCTATCA	
**6HB_B6_019**	TCAGTCCAGTAAGCGTCATCATTGACATTTTTG	
**6HB_B6_020**	CCGTGCGCAGTCTCTGAATGTCAGACTTAATGC	
**6HB_B7_001**	AAGATTCACAACTATGCAGGGAGTTAAGCT	
**6HB_B7_002**	TGCCTGATAATAATTATATTCGGTCGCTTC	
**6HB_B7_003**	CCTCATAAAATCTCCATCGCCCACGCAAAC	
**6HB_B7_004**	AAGGATACCAAAAGGCGCCGACAATGACAC	
**6HB_B7_005**	GAGAAGCTATCGGTACAGCTTGATACCAAA	
**6HB_B7_006**	AGACTTTTGAGGCTAAGGAATTGCGAAGTAA	
**6HB_B7_007**	TCCATTATAACCGATTTTTCACGTTGATATT	
**6HB_B7_008**	GTAATGCCAACAACCAAAAAAAAGGCTAAAA	
**6HB_B7_009**	CAACCTAGATAGTTGAGCCTTTAATTGCTTT	
**6HB_B7_010**	TACAGAGAGGCCGCAGAATAGAAAGGAAAAA	
**6HB_B7_011**	TGTGACCTTCATCAAGAGTCCGCGAGGAAGTT	
**6HB_B7_012**	TTAGACAAGAACCGGATACGCCTGAAAAATAC	
**6HB_B7_013**	TTTCCCAAATCAACGTAAGTACAACAAGGCAC	
**6HB_B7_014**	ATTGCTCATTCAGTGAATATTATACAGAGGCA	
**6HB_B7_015**	GGGAGACCAGGCGCATAGTTAGCCGGGACTAA	
**6HB_B7_016**	ATGACCTGCTCCATGTTACGCTGGCTGTAGGTA	
**6HB_B7_017**	GGGTTAAATTGTGTCGAAATAATCTTAATGCAA	
**6HB_B7_018**	TACGGGAGATTTGTATCATTTCATTATTAGAAC	
**6HB_B7_019**	CGAACAAGCGCGAAACAAACAAAGCTTCAACGC	
**6HB_B7_020**	TTGAGAACGAGGCGCAGACGGTGTACTGAGAAA	
**6HB_B8_001**	CCTCAGAAGTACCTCCCGAAAGACTTCTTT	
**6HB_B8_002**	TAGCAAATTGATAATCAAAAAGATTAATAA	
**6HB_B8_003**	CATACAGCGGATGGCAGAAGCAAAGCGCGT	

**6HB_B8_004**	TAGCATTTGCTGAATTTACCCTGACTACAT	
**6HB_B8_005**	CATCAATCTCAACACATAAATCAAAAACCT	
**6HB_B8_006**	AAAGAAGAAATATCGTCAGGATTAGAGGCAT	
**6HB_B8_007**	GTAATAGGAGGAAGTTAATTGCTCCTTATTA	
**6HB_B8_008**	TGGATAGGATTGCAGAGGTCATTTTTGGCAA	
**6HB_B8_009**	GAATCGTTTATAGTCTTAGAGCTTAATAACA	
**6HB_B8_010**	GAATCCCTCAGGTCTATAATGCTGTAGTCTA	
**6HB_B8_011**	AAAAGATGGTTTAATTTCGAGCAACAGAGGGG	
**6HB_B8_012**	AGCATCATTGTGAATTACAAGGAATTTTAGAC	
**6HB_B8_013**	GGCGATTTTAAGAACTGGTAATGCATACTGCG	
**6HB_B8_014**	TCCTACCAGTCAGGACGTATTTAGGATTCATT	
**6HB_B8_015**	CTAAAAAATCTACGTTAAGTAGAAATGCTTTA	
**6HB_B8_016**	TGCCACTATCATAACCCTCGGGCTTGGCTAAAT	
**6HB_B8_017**	AATGTACGAGGCATAGTAAAACTTTAAATAAAG	
**6HB_B8_018**	CCAAGATACATAACGCCAACTTATGCAAAGAAT	
**6HB_B8_019**	AAATAATACCACATTCAACCTCATTAAATAAAT	
**6HB_B8_020**	CAAAGATTCATCAGTTGAGTGGGAAGATAGTAG	

Supplementary Table 2. DNA sequence for the 6HB closed ring structure with 1-nt gap.

**6HB closed ring (1 gap)**		
**Name**	Sequence (5'→3')	**Color**
**6HB_connector_001**	ACTCACATTAATAGCTATCATTTG	
**6HB_connector_002**	AATAACCTGTTTTGCGTTGCGCTC	
**6HB_connector_003**	CCTGTTGACCATTAGATACATTTC	
**6HB_connector_004**	CGAACGAGTAGATTTAGTTCGTGC	
**6HB_connector_005**	CGGTTTGCGTTTCATTCCATATAAC	
**6HB_connector_006**	GTACGGTGTCTGGAAGTATTGGGCG	
**6HB_LE_1gap_001**	AAGCCTGCCAGTGATCTATCAGGGCCAACGCGCGGGGTTTTT	
**6HB_LE_1gap_002**	CTGCCCGCTTTGCTTTCCTCGTTAGAACGAGCACGTATAACGTCCAGT	
**6HB_LE_1gap_003**	GGGAAACAGCTGCATTAATGAATCGGCGATGGCC	
**6HB_LE_1gap_004**	TTTTCAGGGTGCCTAATGAGTGAGCTACCAGGGTGGAGAG	
**6HB_RE_1gap_001**	GGCGCGAACTAAAAGTTGATTCAACGA	
**6HB_RE_1gap_002**	GCAAAAACAACATTATTACAGTAAAACGAAGGTGG	
**6HB_RE_1gap_003**	AACAGTTCAGAACCAATTCT	
**6HB_RE_1gap_004**	AATGACTGTTTTAAATATGCAGCTGAAAACTAACGGTGGTC	
**6HB_SC_1gap_001**	TGCCAAGTAGATAAAAATATCTTTAGGATTAATGCAATATTT	
**6HB_SC_1gap_002**	AATTAACCGTTGTAGCCTGCAGGTCGACTCTAGAGTCACGC	
**6HB_SC_1gap_003**	TGAATGGCAAAGGAAGGGAAGAAAGCGCTGTCCAGATCCCC	
**6HB_SC_1gap_004**	AGCCGGCTTATAAAGTGGTTCCGAAATCGGAATAGATTAGA	
**6HB_SC_1gap_005**	GCGAGTATTAGTCTGCACTAACAACTCAAAATCCCGAACGT	
**6HB_SC_1gap_006**	CCGTCAACTTGCATGCAATACTTCTTTGCACAGACGCGAACT	

**6HB_SC_1gap_007**	ATTCAGGAGCGGAAAATACCAAGTTACATACCTGAAGGTTGG	
**6HB_SC_1gap_008**	AATGCTACTGTTGGGAAGGGAACAAAGGCAGAGGCGGCAAA	
**6HB_SC_1gap_009**	TTATATAACTAACGCTCAATCGTCTGAAAATACCTGCGGGC	
**6HB_SC_1gap_010**	TGAGAGCCAGCAAATTATTCATTTCAATAAATCGCAAACCA	
**6HB_SC_1gap_011**	GAAAAATTATCATTTTGCGGCGATCGGTACATTTTGTATGT	
**6HB_SC_1gap_012**	CAGAAGGCTGCGCAGATGCAAATCCAATCCGGCTTGCAAAAG	
**6HB_SC_1gap_013**	GGTCACGACGAGCGATTTTGCACCCAGCTGTAGAAAATATCC	
**6HB_SC_1gap_014**	CCGGAATCATAATTAGATGGGCGCATCGTAACCGTATAAAC	
**6HB_SC_1gap_015**	ATCCTAATAATCGTCGCTATTAATTAAAATAAGAGCATCTG	
**6HB_SC_1gap_016**	GAATAACAGAAATATATCAAAATTATTTGCCTGAATCTTAC	
**6HB_SC_1gap_017**	CTGTATTACGAGCATACAATTTTATCACGTAAAACCTTGCT	
**6HB_SC_1gap_018**	AACGCTATTGGTGTACTAGAAAAAGCCTGAAAAATACCAATC	
**6HB_SC_1gap_019**	GCTCATTGGAAGCGAACTGGCATGATTATAGAAAAACCAATG	
**6HB_SC_1gap_020**	CCGTAACAATAGGAACGCCAGAGAGAATTATTACGCAGCAA	
**6HB_SC_1gap_021**	AACCATCGATACAGTAATAAGAGAATAAGAGGCATAATTCG	
**6HB_SC_1gap_022**	ACCGCGCCCAATAGTATGTTAGCAAACGAGACTCCTAACAT	
**6HB_SC_1gap_023**	CAAATCTAGCAGCCTTTACATCAAAAATTTTCGAGCGCAGC	
**6HB_SC_1gap_024**	AAAACAGTTTTAACTCAGTAGCGACAGAAAACGTCTACATAC	
**6HB_SC_1gap_025**	ACGGTAACCAGTACGCAAGCCCAATAGGCTATTTCCGTATAA	
**6HB_SC_1gap_026**	CCGCCTCCCTCAGAACTAGCATGTCAATCATATGTACCGGA	
**6HB_SC_1gap_027**	CAGTTAATGAGGGAGGGAAGGTAAATAAGCCACCACCCCGG	
**6HB_SC_1gap_028**	GGGCGACGCAATAGAATAAGAGCAAGAAACGTAACACTGAG	
**6HB_SC_1gap_029**	CGATTGCCCCCTGCAACCCATGTACCAATGAAATAATTCAA	
**6HB_SC_1gap_030**	TTCGTCATCGTAAAGCCGCCACCCTCAGCAGTGCCGGAACCT	
**6HB_SC_1gap_031**	GGCCGGACGGAGTGTTTTGCGGGATCGTAGGGTAGCATAAGG	
**6HB_SC_1gap_032**	TTGAAAAAATCACCATCAATAACAACTAGCAGCGAAATAGC	
**6HB_SC_1gap_033**	AACCGAACTGACCTCATTAAAGCCAGATCACAAATCAACCG	
**6HB_SC_1gap_034**	GTTGATATAAGTAGACAGCATCGGAACGCACCCTCTTCAAC	
**6HB_SC_1gap_035**	CGGAATATGGGATTTTGCTAATGATATCAAATAAATCCAAC	
**6HB_SC_1gap_036**	GTTTCAGGACAGTCGAGGACAGATGAACGGTCAATCAACGGC	
**6HB_SC_1gap_037**	CGGTTGTCCAACAGGCGTTTTAATTCGAGAGAGGCAGACGAC	
**6HB_SC_1gap_038**	CGAGAAACACCAGAACATTATGACCCTGTAATACTGCCCTG	
**6HB_SC_1gap_039**	ATAAAAACTCATCTTTGACCCCCAGCGAAGGCTTTTTGCGG	
**6HB_SC_1gap_040**	AAAGAATTTCTTAATTATCAGCTTGCTTTCACCAGACCGGA	
**6HB_SC_1gap_041**	AACACCAAAATAGCGCTTCAAAGCGAGAGGTGAATACACTA	
**6HB_SC_1gap_042**	GCAAACTACCAAAAACGAGTAGTAAATTGTTTACCTTTTGCA	
**6HB_B1_1gap_001**	ATAGGGAGCTAAACAGGAGCGCGTATCACCC	
**6HB_B1_1gap_002**	AGCCGGTGATTGCCGTCAAAGGGCGATGA	
**6HB_B1_1gap_003**	CACTAAAGGGATTTTAGAGCGCTTAGGGTCG	
**6HB_B1_1gap_004**	CACAATGCCCTGATTAAAGAACGTGGTTT	
**6HB_B1_1gap_005**	TGTGGTACGCCAGAATCCGCGCGTACTAAAT	
**6HB_B1_1gap_006**	TGTGTGAGCGGTCTTTGGAACAAGAGTAA	
**6HB_B1_1gap_007**	TAGTGTTTTTATAATCAGTGGCAAGAGCCCC	
**6HB_B1_1gap_008**	ATCATGCCCAGCAAGGGTTGAGTGTTTAA	

**6HB_B1_1gap_009**	TCGACCGAGTAAAAGAGTAAAGGAGCGGGGA	
**6HB_B1_1gap_010**	GGTACCTTTGATGTCAAAAGAATAGCAGC	
**6HB_B1_1gap_011**	ACTACGAAAACCGGACGGGCAACAGCAAGC	
**6HB_B1_1gap_012**	ACCACTATGGTTGCTTTGATCAGAGCAAGTGT	
**6HB_B1_1gap_013**	AATCAAGACTCCAACCTTCACCGCCTGTCCA	
**6HB_B1_1gap_014**	GGTGCCGTCCACTAGAGAGTTGCAGCAAAAT	
**6HB_B1_1gap_015**	GGAACCCGTTCCAGCACGCTGGTTTGCGTCA	
**6HB_B1_1gap_016**	GATTTAGCCGAGATGGCGAAAATCCTGGAGC	
**6HB_B1_1gap_017**	TTTGATGCGCCGCTACAGGGGCCGATAACATAC	
**6HB_B1_1gap_018**	AGCAACCACCACACCCGCCCAGGAACTATCCGC	
**6HB_B1_1gap_019**	AGGGTGTAGCGGTCACGCTTGAGAAGCTGTTTC	
**6HB_B1_1gap_020**	TTGACGGGCGCTAGGGCGCTGAGGCCAATTCGT	
**6HB_B2_1gap_001**	CGAATAACATCACTTGCCCTGAAAGATCGCC	
**6HB_B2_1gap_002**	CGTTGTTTAGAAGGAATTGAGGAAGGCTA	
**6HB_B2_1gap_003**	CCCAAGAACTCAAACTATACAGAGACGAACC	
**6HB_B2_1gap_004**	CCAGGGAAACAATGGCAAATCAACAGTAC	
**6HB_B2_1gap_005**	GTTGCTGGTAATATCCAGAATAAAAAACAGA	
**6HB_B2_1gap_006**	AGGCGATTAAATCCAATCAATATCTGAAG	
**6HB_B2_1gap_007**	GATTTACCGCCAGCCATTATTCACCATTAAC	
**6HB_B2_1gap_008**	GCGAAATTATTAAAACCTCAAATATCGGT	
**6HB_B2_1gap_009**	TACGAAAAACGCTCATGGAATGGATGCCACG	
**6HB_B2_1gap_010**	TCTTCGAGTAACATCTAAAGCATCACCAA	
**6HB_B2_1gap_011**	ATAGCCTTATCTATACATTTGAGGATAAAA	
**6HB_B2_1gap_012**	AAACCGTAAGAATACGTGGATTAGTACGGCCA	
**6HB_B2_1gap_013**	TTAAAAATTGAAAGTATTAGACTTTACTTTT	
**6HB_B2_1gap_014**	CCAGCAGGTCAGTTTCGACAACTCGTATTAA	
**6HB_B2_1gap_015**	GTGAGGCAAACCCTCTTTGCCCGAACGGGGG	
**6HB_B2_1gap_016**	CCGCCTGCTTGCTGTTTTAAAAGTTTGCTAT	
**6HB_B2_1gap_017**	CGAATAGAACCCTTCTGACTGAGTAGAGTCACG	
**6HB_B2_1gap_018**	ATAAGGGACATTCTGGCCACGGCCTTGGGTAAC	
**6HB_B2_1gap_019**	CAGTAGTCACACGACCAGTAACAATAGTGCTGC	
**6HB_B2_1gap_020**	CAGTTATTTACATTGGCAGGCAACAGGCCAGCT	
**6HB_B3_1gap_001**	GCCCAAAGAACGCGAGAATGAGAGAAACATC	
**6HB_B3_1gap_002**	CAGGCACCTGATTGGATTCGCCTGATTGA	
**6HB_B3_1gap_003**	GTGTCAAATATATTTTAGGAATTTAATTACA	
**6HB_B3_1gap_004**	ACCGCTAATTCATACATCGGGAGAAAAAA	
**6HB_B3_1gap_005**	AGCCATCTTCTGACCTAAGACGCTGTGAATT	
**6HB_B3_1gap_006**	CACTCCATTGTTTATACAGTAACAGTTTT	
**6HB_B3_1gap_007**	CAGGGTTTGAAATACCGAACATAGCAACAGT	
**6HB_B3_1gap_008**	GTATCGGAATAATAGGTTTAACGTCATAA	
**6HB_B3_1gap_009**	GGAATAAATAAGGCGTTATTTTCCCTGTGAG	
**6HB_B3_1gap_010**	CAGTTTCCTACCAAAGAAATTGCGTACAA	
**6HB_B3_1gap_011**	AGATGATGCTTTGTTATCATCATATTAAGC	
**6HB_B3_1gap_012**	AACACTACCTTTTTAACCTCGCAAGAATTCGC	

6HB_B3_1gap_013	AGAAAACCAATAACATCAGATGATGGCTCTG	
6HB_B3_1gap_014	TTAACAAACCTTTTCAATATAATCCTGAGCC	
6HB_B3_1gap_015	CCTTTTTGATGAATGGATTATACTTCTGCCT	
6HB_B3_1gap_016	CATAAATGATTTTCGGAAGGGTTAGAAGAGG	
6HB_B3_1gap_017	ATTATCAAAATCATAGGTCAACTTTTCCGGAAA	
6HB_B3_1gap_018	CATTAGAAGAGTCAATAGTTTAATTTTTTCCGG	
6HB_B3_1gap_019	TGGAGATAGCTTAGATTAAATTTAATGAAGATC	
6HB_B3_1gap_020	TATATTAGAATCCTTGAAACCGTGTGCGACGAC	
6HB_B4_1gap_001	TAAATCATATGCGTTATAACAATAGTTTCCT	
6HB_B4_1gap_002	GGATTGTAATTTGTTAAATCAAGATTGGC	
6HB_B4_1gap_003	GAATTACCAGTATAAAGCTGCAGAAGGGTAT	
6HB_B4_1gap_004	TTCTCCAAACAGCTTGCGGGAGGTTTCAA	
6HB_B4_1gap_005	AACCAACAGTAGGGCTTAAATAAACACTCAT	
6HB_B4_1gap_006	AGCGAGCCAATCCGTTTTAGCGAACCGTA	
6HB_B4_1gap_007	CATATCGCCATATTTAACGTAATTCCGTTTT	
6HB_B4_1gap_008	CTTTCATTTTTTGGTATTCTAAGAACAGC	
6HB_B4_1gap_009	CCTACATGTAATTTAGGCTAAAGTAAATCAT	
6HB_B4_1gap_010	GTCTGGATGAAAAAGATATAGAAGGCTCG	
6HB_B4_1gap_011	ATAATCAGTTGCTTCTTTCCAGAGCCACCG	
6HB_B4_1gap_012	TGTCATAAGTCCTGAACAAGTTTAGTTGGGAT	
6HB_B4_1gap_013	ATCATTCTGAAGCCCCAGTTACAAAATGTGG	
6HB_B4_1gap_014	AAACCAATCCCGACCATATTATTTATCTAAC	
6HB_B4_1gap_015	GAGAACAGCGAGGCAAATAAGAAACGATCAA	
6HB_B4_1gap_016	ATTTTCATTATCCGTTTAACGTCAAAACCTT	
6HB_B4_1gap_017	GAACCGCGCCTGTTTATCACAAATTCCAAACGG	
6HB_B4_1gap_018	CCGCAACATGTTCAGCTAACAACGCTCCGTCGG	
6HB_B4_1gap_019	AAGCTGTCCAGACGACGACATTGAGATAAATGT	
6HB_B4_1gap_020	TAGGCCGACAAAAGGTAAAAACGCCAGTAGCCA	
6HB_B5_1gap_001	TGTTTGCCTTTAGCGTCACCATTACATATAA	
6HB_B5_1gap_002	ATTAAAAATTAACAATAACGGAATACTGG	
6HB_B5_1gap_003	TTAGCGCGTTTTCATCGGGCCAGCAACCACG	
6HB_B5_1gap_004	TAATATCAAAGTCGGAAACCGAGGAACAA	
6HB_B5_1gap_005	ATTGGTCATAGCCCCCTTCTTGAGCGTCACA	
6HB_B5_1gap_006	AAATATGCGCTAAGCCGAACAAAGTTTTA	
6HB_B5_1gap_007	ATTTTTGCCATCTTTTCAGGTGAATATATGG	
6HB_B5_1gap_008	AAACAGACCCACATTAAGAAAAGTAAAAA	
6HB_B5_1gap_009	AAAAATCACCGGAACCAGTTGACGGGACAAA	
6HB_B5_1gap_010	TGATAAGCCCAATCTATCTTACCGAACGC	
6HB_B5_1gap_011	TAAAGGCCAAAAGCATTAGACGGGAGTTTT	
6HB_B5_1gap_012	CAACCATTAGCAAGGCCGGATCAAGTTAAATC	
6HB_B5_1gap_013	AGAAACGACGCAATTGAACACCCTGAATTTG	
6HB_B5_1gap_014	AATAAGTACCAGAAAGAGGGTAATTGATTAA	
6HB_B5_1gap_015	TCAATAGGCAGATATATCAGAGAGATAGAAG	
6HB_B5_1gap_016	TTACCAGGCCCTTTAGAATTGAGTTAATCAG	

**6HB_B5_1gap_017**	AGACAAATCACCAGTAGCAGACTGTAAAATTCG	
**6HB_B5_1gap_018**	TTTTCATTTGGGAATTAGACATTTTCGTAAACG	
**6HB_B5_1gap_019**	ATTCTATCACCGTCACCGAATTAGCGGTATAAG	
**6HB_B5_1gap_020**	CAAAAAATTATTCATTAAATAATCAAAGCCCCA	
**6HB_B6_1gap_001**	GAAACCCTCAGAGCCACCACGGGGTTGAAAG	
**6HB_B6_1gap_002**	GCAAACTGTAGCACACCCTCATTTTCTGA	
**6HB_B6_1gap_003**	GAGGAGCCGCCACCAGAAAGTGTACACTCCT	
**6HB_B6_1gap_004**	GTCATTCCTCATAACCGCCACCCTCAGGC	
**6HB_B6_1gap_005**	AAGCAGAGCCGCCGCCAGACATGGCGATTAG	
**6HB_B6_1gap_006**	GAGATCTCTAAAGTCAGAACCGCCACGGA	
**6HB_B6_1gap_007**	GCTAGGAGGTTGAGGCAGTTACCGTTACCAG	
**6HB_B6_1gap_008**	GGAGAGCCAGACGGAGGTTTAGTACCTGC	
**6HB_B6_1gap_009**	AAAGATTGGCCTTGATATATGGAAACGAGAG	
**6HB_B6_1gap_010**	TCTAGCTTTCTGTAGGTGTATCACCGGTG	
**6HB_B6_1gap_011**	TTATTCAGGGATAAAACTACAACGCCAAGA	
**6HB_B6_1gap_012**	AACACAGTGCCTTGAGTAAAACCGCCTCGATG	
**6HB_B6_1gap_013**	ATTAAGAGAGCCACTTCCACAGACAGCGCCT	
**6HB_B6_1gap_014**	AAGAGAACCTCAGAGTTAGCGTAACGATACA	
**6HB_B6_1gap_015**	GGGGTTTGCCACCCTTTTGTCGTCTTTGGTA	
**6HB_B6_1gap_016**	CGGATAATACTCAGTTAGTAAATGAATTGAT	
**6HB_B6_1gap_017**	TGAGTGGTAATAAGTTTTAACCCTCAAGTCTGG	
**6HB_B6_1gap_018**	TTAGTTTTGATGATACAGGCCACCACGCTATCA	
**6HB_B6_1gap_019**	TCAGTCCAGTAAGCGTCATCATTGACATTTTTG	
**6HB_B6_1gap_020**	CCGTGCGCAGTCTCTGAATGTCAGACTTAATGC	
**6HB_B7_1gap_001**	TGTGACCTTCATCAAGAGTCCGCGAGGAAGT	
**6HB_B7_1gap_002**	GCCTGATAATAATTATATTCGGTCGCTTC	
**6HB_B7_1gap_003**	TTAGACAAGAACCGGATACGCCTGAAAAATA	
**6HB_B7_1gap_004**	CTCATAAAATCTCCATCGCCCACGCAAAC	
**6HB_B7_1gap_005**	TTTCCCAAATCAACGTAAGTACAACAAGGCA	
**6HB_B7_1gap_006**	AGGATACCAAAAGGCGCCGACAATGACAC	
**6HB_B7_1gap_007**	ATTGCTCATTCAGTGAATATTATACAGAGGC	
**6HB_B7_1gap_008**	AGAAGCTATCGGTACAGCTTGATACCAAA	
**6HB_B7_1gap_009**	GGGAGACCAGGCGCATAGTTAGCCGGGACTA	
**6HB_B7_1gap_010**	AGATTCACAACTATGCAGGGAGTTAAGCT	
**6HB_B7_1gap_011**	ACAGAGAGGCCGCAGAATAGAAAGGAAAAA	
**6HB_B7_1gap_012**	TTGAGAACGAGGCGCAGACGGTGTACTGAGAA	
**6HB_B7_1gap_013**	AGACTTTTGAGGCTAAGGAATTGCGAAGTAA	
**6HB_B7_1gap_014**	TCCATTATAACCGATTTTTCACGTTGATATT	
**6HB_B7_1gap_015**	GTAATGCCAACAACCAAAAAAAAGGCTAAAA	
**6HB_B7_1gap_016**	CAACCTAGATAGTTGAGCCTTTAATTGCTTT	
**6HB_B7_1gap_017**	ATGACCTGCTCCATGTTACGCTGGCTGTAGGTA	
**6HB_B7_1gap_018**	GGGTTAAATTGTGTCGAAATAATCTTAATGCAA	
**6HB_B7_1gap_019**	TACGGGAGATTTGTATCATTTCATTATTAGAAC	
**6HB_B7_1gap_020**	CGAACAAGCGCGAAACAAACAAAGCTTCAACGC	

6HB_B8_1gap_001	AAAAGATGGTTTAATTTCGAGCAACAGAGGG	
6HB_B8_1gap_002	CTCAGAAGTACCTCCCGAAAGACTTCTTT	
6HB_B8_1gap_003	AGCATCATTGTGAATTACAAGGAATTTTAGA	
6HB_B8_1gap_004	AGCAAATTGATAATCAAAAAGATTAATAA	
6HB_B8_1gap_005	GGCGATTTTAAGAACTGGTAATGCATACTGC	
6HB_B8_1gap_006	ATACAGCGGATGGCAGAAGCAAAGCGCGT	
6HB_B8_1gap_007	TCCTACCAGTCAGGACGTATTTAGGATTCAT	
6HB_B8_1gap_008	AGCATTTGCTGAATTTACCCTGACTACAT	
6HB_B8_1gap_009	CTAAAAAATCTACGTTAAGTAGAAATGCTTT	
6HB_B8_1gap_010	ATCAATCTCAACACATAAATCAAAAACCT	
6HB_B8_1gap_011	AAGAAGAAATATCGTCAGGATTAGAGGCAT	
6HB_B8_1gap_012	TGCCACTATCATAACCCTCGGGCTTGGCTAAA	
6HB_B8_1gap_013	GTAATAGGAGGAAGTTAATTGCTCCTTATTA	
6HB_B8_1gap_014	TGGATAGGATTGCAGAGGTCATTTTTGGCAA	
6HB_B8_1gap_015	GAATCGTTTATAGTCTTAGAGCTTAATAACA	
6HB_B8_1gap_016	GAATCCCTCAGGTCTATAATGCTGTAGTCTA	
6HB_B8_1gap_017	AATGTACGAGGCATAGTAAAACTTTAAATAAAG	
6HB_B8_1gap_018	CCAAGATACATAACGCCAACTTATGCAAAGAAT	
6HB_B8_1gap_019	AAATAATACCACATTCAACCTCATTAAATAAAT	
6HB_B8_1gap_020	CAAAGATTCATCAGTTGAGTGGGAAGATAGTAG	

Supplementary Table 3. DNA sequence for the 6HB closed ring structure with 3-nt gap.

6HB closed ring (3 gap)		
**Name**	**Sequence (5'→3')**	**Color**
6HB_connector_001	ACTCACATTAATAGCTATCATTTG	
6HB_connector_002	AATAACCTGTTTTGCGTTGCGCTC	
6HB_connector_003	CCTGTTGACCATTAGATACATTTC	
6HB_connector_004	CGAACGAGTAGATTTAGTTCGTGC	
6HB_connector_005	CGGTTTGCGTTTCATTCCATATAAC	
6HB_connector_006	GTACGGTGTCTGGAAGTATTGGGCG	
6HB_LE_3gap_001	AAGCCTGCCAGTGATCTATCAGGGCCAACGCGCGGGGTTTTT	
6HB_LE_3gap_002	GCCCGCTTTGCTTTCCTCGTTAGAACGAGCACGTATAACGTCCAGT	
6HB_LE_3gap_003	GAAACAGCTGCATTAATGAATCGGCGATGGCC	
6HB_LE_3gap_004	TTCAGGGTGCCTAATGAGTGAGCTACCAGGGTGGAG	
6HB_RE_3gap_001	CGCGAACTAAAAGTTGATTCAACGA	
6HB_RE_3gap_002	GCAAAAACAACATTATTACAGTAAAACGAAGGTGG	
6HB_RE_3gap_003	AACAGTTCAGAACCAATT	
6HB_RE_3gap_004	TGACTGTTTTAAATATGCAGCTGAAAACTAACGGTGGTC	
6HB_SC_3gap_001	TGCCAAGTAGATAAAAATATCTTTAGGATTAATGCAATATTT	
6HB_SC_3gap_002	AATTAACCGTTGTAGCCTGCAGGTCGACTCTAGAGTCAC	
6HB_SC_3gap_003	AATGGCAAAGGAAGGGAAGAAAGCGCTGTCCAGATCCCC	
6HB_SC_3gap_004	AGCCGGCTTATAAAGTGGTTCCGAAATCGGAATAGATTA	

**6HB_SC_3gap_005**	GCGAGTATTAGTCTGCACTAACAACTCAAAATCCCGAAC	
**6HB_SC_3gap_006**	CCGTCAACTTGCATGCAATACTTCTTTGCACAGACGCGAACT	
**6HB_SC_3gap_007**	ATTCAGGAGCGGAAAATACCAAGTTACATACCTGAAGGTTGG	
**6HB_SC_3gap_008**	TGCTACTGTTGGGAAGGGAACAAAGGCAGAGGCGGCAAA	
**6HB_SC_3gap_009**	ATATAACTAACGCTCAATCGTCTGAAAATACCTGCGGGC	
**6HB_SC_3gap_010**	TGAGAGCCAGCAAATTATTCATTTCAATAAATCGCAAAC	
**6HB_SC_3gap_011**	AAAATTATCATTTTGCGGCGATCGGTACATTTTGTATGT	
**6HB_SC_3gap_012**	CAGAAGGCTGCGCAGATGCAAATCCAATCCGGCTTGCAAAAG	
**6HB_SC_3gap_013**	GGTCACGACGAGCGATTTTGCACCCAGCTGTAGAAAATATCC	
**6HB_SC_3gap_014**	CCGGAATCATAATTAGATGGGCGCATCGTAACCGTATAA	
**6HB_SC_3gap_015**	CCTAATAATCGTCGCTATTAATTAAAATAAGAGCATCTG	
**6HB_SC_3gap_016**	GAATAACAGAAATATATCAAAATTATTTGCCTGAATCTT	
**6HB_SC_3gap_017**	CTGTATTACGAGCATACAATTTTATCACGTAAAACCTTG	
**6HB_SC_3gap_018**	AACGCTATTGGTGTACTAGAAAAAGCCTGAAAAATACCAATC	
**6HB_SC_3gap_019**	GCTCATTGGAAGCGAACTGGCATGATTATAGAAAAACCAATG	
**6HB_SC_3gap_020**	GTAACAATAGGAACGCCAGAGAGAATTATTACGCAGCAA	
**6HB_SC_3gap_021**	CCATCGATACAGTAATAAGAGAATAAGAGGCATAATTCG	
**6HB_SC_3gap_022**	ACCGCGCCCAATAGTATGTTAGCAAACGAGACTCCTAAC	
**6HB_SC_3gap_023**	AATCTAGCAGCCTTTACATCAAAAATTTTCGAGCGCAGC	
**6HB_SC_3gap_024**	AAAACAGTTTTAACTCAGTAGCGACAGAAAACGTCTACATAC	
**6HB_SC_3gap_025**	ACGGTAACCAGTACGCAAGCCCAATAGGCTATTTCCGTATAA	
**6HB_SC_3gap_026**	CCGCCTCCCTCAGAACTAGCATGTCAATCATATGTACCG	
**6HB_SC_3gap_027**	GTTAATGAGGGAGGGAAGGTAAATAAGCCACCACCCCGG	
**6HB_SC_3gap_028**	GGGCGACGCAATAGAATAAGAGCAAGAAACGTAACACTG	
**6HB_SC_3gap_029**	CGATTGCCCCCTGCAACCCATGTACCAATGAAATAATTC	
**6HB_SC_3gap_030**	TTCGTCATCGTAAAGCCGCCACCCTCAGCAGTGCCGGAACCT	
**6HB_SC_3gap_031**	GGCCGGACGGAGTGTTTTGCGGGATCGTAGGGTAGCATAAGG	
**6HB_SC_3gap_032**	GAAAAAATCACCATCAATAACAACTAGCAGCGAAATAGC	
**6HB_SC_3gap_033**	CCGAACTGACCTCATTAAAGCCAGATCACAAATCAACCG	
**6HB_SC_3gap_034**	GTTGATATAAGTAGACAGCATCGGAACGCACCCTCTTCA	
**6HB_SC_3gap_035**	GAATATGGGATTTTGCTAATGATATCAAATAAATCCAAC	
**6HB_SC_3gap_036**	GTTTCAGGACAGTCGAGGACAGATGAACGGTCAATCAACGGC	
**6HB_SC_3gap_037**	CGGTTGTCCAACAGGCGTTTTAATTCGAGAGAGGCAGACGAC	
**6HB_SC_3gap_038**	CGAGAAACACCAGAACATTATGACCCTGTAATACTGCCC	
**6HB_SC_3gap_039**	AAAAACTCATCTTTGACCCCCAGCGAAGGCTTTTTGCGG	
**6HB_SC_3gap_040**	AAAGAATTTCTTAATTATCAGCTTGCTTTCACCAGACCG	
**6HB_SC_3gap_041**	AACACCAAAATAGCGCTTCAAAGCGAGAGGTGAATACAC	
**6HB_SC_3gap_042**	GCAAACTACCAAAAACGAGTAGTAAATTGTTTACCTTTTGCA	
**6HB_B1_3gap_001**	CCGGTGATTGCCGTCAAAGGGCGATGA	
**6HB_B1_3gap_002**	CAATGCCCTGATTAAAGAACGTGGTTT	
**6HB_B1_3gap_003**	TGTGAGCGGTCTTTGGAACAAGAGTAA	
**6HB_B1_3gap_004**	CATGCCCAGCAAGGGTTGAGTGTTTAA	
**6HB_B1_3gap_005**	TACCTTTGATGTCAAAAGAATAGCAGC	
**6HB_B1_3gap_006**	ACCACTATGGTTGCTTTGATCAGAGCAAGT	

**6HB_B1_3gap_007**	AATCAAGACTCCAACCTTCACCGCCTGTCCA	
**6HB_B1_3gap_008**	GGTGCCGTCCACTAGAGAGTTGCAGCAAAAT	
**6HB_B1_3gap_009**	GGAACCCGTTCCAGCACGCTGGTTTGCGTCA	
**6HB_B1_3gap_010**	GATTTAGCCGAGATGGCGAAAATCCTGGAGC	
**6HB_B1_3gap_011**	ATAGGGAGCTAAACAGGAGCGCGTATCAC	
**6HB_B1_3gap_012**	CACTAAAGGGATTTTAGAGCGCTTAGGGT	
**6HB_B1_3gap_013**	TGTGGTACGCCAGAATCCGCGCGTACTAA	
**6HB_B1_3gap_014**	TAGTGTTTTTATAATCAGTGGCAAGAGCC	
**6HB_B1_3gap_015**	TCGACCGAGTAAAAGAGTAAAGGAGCGGG	
**6HB_B1_3gap_016**	TACGAAAACCGGACGGGCAACAGCAAGC	
**6HB_B1_3gap_017**	TTTGATGCGCCGCTACAGGGGCCGATAACATAC	
**6HB_B1_3gap_018**	AGCAACCACCACACCCGCCCAGGAACTATCCGC	
**6HB_B1_3gap_019**	AGGGTGTAGCGGTCACGCTTGAGAAGCTGTTTC	
**6HB_B1_3gap_020**	TTGACGGGCGCTAGGGCGCTGAGGCCAATTCGT	
**6HB_B2_3gap_001**	AGCCTTATCTATACATTTGAGGATAAAA	
**6HB_B2_3gap_002**	TTGTTTAGAAGGAATTGAGGAAGGCTA	
**6HB_B2_3gap_003**	AGGGAAACAATGGCAAATCAACAGTAC	
**6HB_B2_3gap_004**	GCGATTAAATCCAATCAATATCTGAAG	
**6HB_B2_3gap_005**	GAAATTATTAAAACCTCAAATATCGGT	
**6HB_B2_3gap_006**	TTCGAGTAACATCTAAAGCATCACCAA	
**6HB_B2_3gap_007**	TTAAAAATTGAAAGTATTAGACTTTACTTTT	
**6HB_B2_3gap_008**	CCAGCAGGTCAGTTTCGACAACTCGTATTAA	
**6HB_B2_3gap_009**	GTGAGGCAAACCCTCTTTGCCCGAACGGGGG	
**6HB_B2_3gap_010**	CCGCCTGCTTGCTGTTTTAAAAGTTTGCTAT	
**6HB_B2_3gap_011**	CGAATAACATCACTTGCCCTGAAAGATCG	
**6HB_B2_3gap_012**	CCCAAGAACTCAAACTATACAGAGACGAA	
**6HB_B2_3gap_013**	GTTGCTGGTAATATCCAGAATAAAAAACA	
**6HB_B2_3gap_014**	GATTTACCGCCAGCCATTATTCACCATTA	
**6HB_B2_3gap_015**	TACGAAAAACGCTCATGGAATGGATGCCA	
**6HB_B2_3gap_016**	AAACCGTAAGAATACGTGGATTAGTACGGC	
**6HB_B2_3gap_017**	CGAATAGAACCCTTCTGACTGAGTAGAGTCACG	
**6HB_B2_3gap_018**	ATAAGGGACATTCTGGCCACGGCCTTGGGTAAC	
**6HB_B2_3gap_019**	CAGTAGTCACACGACCAGTAACAATAGTGCTGC	
**6HB_B2_3gap_020**	CAGTTATTTACATTGGCAGGCAACAGGCCAGCT	
**6HB_B3_3gap_001**	AGAAAACCAATAACATCAGATGATGGCTCTG	
**6HB_B3_3gap_002**	TTAACAAACCTTTTCAATATAATCCTGAGCC	
**6HB_B3_3gap_003**	CCTTTTTGATGAATGGATTATACTTCTGCCT	
**6HB_B3_3gap_004**	CATAAATGATTTTCGGAAGGGTTAGAAGAGG	
**6HB_B3_3gap_005**	GGCACCTGATTGGATTCGCCTGATTGA	
**6HB_B3_3gap_006**	CGCTAATTCATACATCGGGAGAAAAAA	
**6HB_B3_3gap_007**	CTCCATTGTTTATACAGTAACAGTTTT	
**6HB_B3_3gap_008**	ATCGGAATAATAGGTTTAACGTCATAA	
**6HB_B3_3gap_009**	GTTTCCTACCAAAGAAATTGCGTACAA	
**6HB_B3_3gap_010**	ATGATGCTTTGTTATCATCATATTAAGC	

**6HB_B3_3gap_011**	ATTATCAAAATCATAGGTCAACTTTTCCGGAAA	
**6HB_B3_3gap_012**	CATTAGAAGAGTCAATAGTTTAATTTTTTCCGG	
**6HB_B3_3gap_013**	TGGAGATAGCTTAGATTAAATTTAATGAAGATC	
**6HB_B3_3gap_014**	TATATTAGAATCCTTGAAACCGTGTGCGACGAC	
**6HB_B3_3gap_015**	GCCCAAAGAACGCGAGAATGAGAGAAACA	
**6HB_B3_3gap_016**	GTGTCAAATATATTTTAGGAATTTAATTA	
**6HB_B3_3gap_017**	AGCCATCTTCTGACCTAAGACGCTGTGAA	
**6HB_B3_3gap_018**	CAGGGTTTGAAATACCGAACATAGCAACA	
**6HB_B3_3gap_019**	GGAATAAATAAGGCGTTATTTTCCCTGTG	
**6HB_B3_3gap_020**	AACACTACCTTTTTAACCTCGCAAGAATTC	
**6HB_B4_3gap_001**	AATCAGTTGCTTCTTTCCAGAGCCACCG	
**6HB_B4_3gap_002**	ATCATTCTGAAGCCCCAGTTACAAAATGTGG	
**6HB_B4_3gap_003**	AAACCAATCCCGACCATATTATTTATCTAAC	
**6HB_B4_3gap_004**	GAGAACAGCGAGGCAAATAAGAAACGATCAA	
**6HB_B4_3gap_005**	ATTTTCATTATCCGTTTAACGTCAAAACCTT	
**6HB_B4_3gap_006**	ATTGTAATTTGTTAAATCAAGATTGGC	
**6HB_B4_3gap_007**	CTCCAAACAGCTTGCGGGAGGTTTCAA	
**6HB_B4_3gap_008**	CGAGCCAATCCGTTTTAGCGAACCGTA	
**6HB_B4_3gap_009**	TTCATTTTTTGGTATTCTAAGAACAGC	
**6HB_B4_3gap_010**	CTGGATGAAAAAGATATAGAAGGCTCG	
**6HB_B4_3gap_011**	GAACCGCGCCTGTTTATCACAAATTCCAAACGG	
**6HB_B4_3gap_012**	CCGCAACATGTTCAGCTAACAACGCTCCGTCGG	
**6HB_B4_3gap_013**	AAGCTGTCCAGACGACGACATTGAGATAAATGT	
**6HB_B4_3gap_014**	TAGGCCGACAAAAGGTAAAAACGCCAGTAGCCA	
**6HB_B4_3gap_015**	TAAATCATATGCGTTATAACAATAGTTTC	
**6HB_B4_3gap_016**	GAATTACCAGTATAAAGCTGCAGAAGGGT	
**6HB_B4_3gap_017**	AACCAACAGTAGGGCTTAAATAAACACTC	
**6HB_B4_3gap_018**	CATATCGCCATATTTAACGTAATTCCGTT	
**6HB_B4_3gap_019**	CCTACATGTAATTTAGGCTAAAGTAAATC	
**6HB_B4_3gap_020**	TGTCATAAGTCCTGAACAAGTTTAGTTGGG	
**6HB_B5_3gap_001**	TAAAAATTAACAATAACGGAATACTGG	
**6HB_B5_3gap_002**	ATATCAAAGTCGGAAACCGAGGAACAA	
**6HB_B5_3gap_003**	ATATGCGCTAAGCCGAACAAAGTTTTA	
**6HB_B5_3gap_004**	ACAGACCCACATTAAGAAAAGTAAAAA	
**6HB_B5_3gap_005**	ATAAGCCCAATCTATCTTACCGAACGC	
**6HB_B5_3gap_006**	AAGGCCAAAAGCATTAGACGGGAGTTTT	
**6HB_B5_3gap_007**	AGAAACGACGCAATTGAACACCCTGAATTTG	
**6HB_B5_3gap_008**	AATAAGTACCAGAAAGAGGGTAATTGATTAA	
**6HB_B5_3gap_009**	TCAATAGGCAGATATATCAGAGAGATAGAAG	
**6HB_B5_3gap_010**	TTACCAGGCCCTTTAGAATTGAGTTAATCAG	
**6HB_B5_3gap_011**	TGTTTGCCTTTAGCGTCACCATTACATAT	
**6HB_B5_3gap_012**	TTAGCGCGTTTTCATCGGGCCAGCAACCA	
**6HB_B5_3gap_013**	ATTGGTCATAGCCCCCTTCTTGAGCGTCA	
**6HB_B5_3gap_014**	ATTTTTGCCATCTTTTCAGGTGAATATAT	

**6HB_B5_3gap_015**	AAAAATCACCGGAACCAGTTGACGGGACA	
**6HB_B5_3gap_016**	CAACCATTAGCAAGGCCGGATCAAGTTAAA	
**6HB_B5_3gap_017**	AGACAAATCACCAGTAGCAGACTGTAAAATTCG	
**6HB_B5_3gap_018**	TTTTCATTTGGGAATTAGACATTTTCGTAAACG	
**6HB_B5_3gap_019**	ATTCTATCACCGTCACCGAATTAGCGGTATAAG	
**6HB_B5_3gap_020**	CAAAAAATTATTCATTAAATAATCAAAGCCCCA	
**6HB_B6_3gap_001**	AAACTGTAGCACACCCTCATTTTCTGA	
**6HB_B6_3gap_002**	CATTCCTCATAACCGCCACCCTCAGGC	
**6HB_B6_3gap_003**	GATCTCTAAAGTCAGAACCGCCACGGA	
**6HB_B6_3gap_004**	AGAGCCAGACGGAGGTTTAGTACCTGC	
**6HB_B6_3gap_005**	TAGCTTTCTGTAGGTGTATCACCGGTG	
**6HB_B6_3gap_006**	ATTCAGGGATAAAACTACAACGCCAAGA	
**6HB_B6_3gap_007**	ATTAAGAGAGCCACTTCCACAGACAGCGCCT	
**6HB_B6_3gap_008**	AAGAGAACCTCAGAGTTAGCGTAACGATACA	
**6HB_B6_3gap_009**	GGGGTTTGCCACCCTTTTGTCGTCTTTGGTA	
**6HB_B6_3gap_010**	CGGATAATACTCAGTTAGTAAATGAATTGAT	
**6HB_B6_3gap_011**	GAAACCCTCAGAGCCACCACGGGGTTGAA	
**6HB_B6_3gap_012**	GAGGAGCCGCCACCAGAAAGTGTACACTC	
**6HB_B6_3gap_013**	AAGCAGAGCCGCCGCCAGACATGGCGATT	
**6HB_B6_3gap_014**	GCTAGGAGGTTGAGGCAGTTACCGTTACC	
**6HB_B6_3gap_015**	AAAGATTGGCCTTGATATATGGAAACGAG	
**6HB_B6_3gap_016**	AACACAGTGCCTTGAGTAAAACCGCCTCGA	
**6HB_B6_3gap_017**	TGAGTGGTAATAAGTTTTAACCCTCAAGTCTGG	
**6HB_B6_3gap_018**	TTAGTTTTGATGATACAGGCCACCACGCTATCA	
**6HB_B6_3gap_019**	TCAGTCCAGTAAGCGTCATCATTGACATTTTTG	
**6HB_B6_3gap_020**	CCGTGCGCAGTCTCTGAATGTCAGACTTAATGC	
**6HB_B7_3gap_001**	TGTGACCTTCATCAAGAGTCCGCGAGGAA	
**6HB_B7_3gap_002**	TTAGACAAGAACCGGATACGCCTGAAAAA	
**6HB_B7_3gap_003**	TTTCCCAAATCAACGTAAGTACAACAAGG	
**6HB_B7_3gap_004**	ATTGCTCATTCAGTGAATATTATACAGAG	
**6HB_B7_3gap_005**	GGGAGACCAGGCGCATAGTTAGCCGGGAC	
**6HB_B7_3gap_006**	AGACTTTTGAGGCTAAGGAATTGCGAAGTAA	
**6HB_B7_3gap_007**	TCCATTATAACCGATTTTTCACGTTGATATT	
**6HB_B7_3gap_008**	GTAATGCCAACAACCAAAAAAAAGGCTAAAA	
**6HB_B7_3gap_009**	CAACCTAGATAGTTGAGCCTTTAATTGCTTT	
**6HB_B7_3gap_010**	AGAGAGGCCGCAGAATAGAAAGGAAAAA	
**6HB_B7_3gap_011**	CTGATAATAATTATATTCGGTCGCTTC	
**6HB_B7_3gap_012**	CATAAAATCTCCATCGCCCACGCAAAC	
**6HB_B7_3gap_013**	GATACCAAAAGGCGCCGACAATGACAC	
**6HB_B7_3gap_014**	AAGCTATCGGTACAGCTTGATACCAAA	
**6HB_B7_3gap_015**	ATTCACAACTATGCAGGGAGTTAAGCT	
**6HB_B7_3gap_016**	ATGACCTGCTCCATGTTACGCTGGCTGTAGGTA	
**6HB_B7_3gap_017**	GGGTTAAATTGTGTCGAAATAATCTTAATGCAA	
**6HB_B7_3gap_018**	TACGGGAGATTTGTATCATTTCATTATTAGAAC	

**6HB_B7_3gap_019**	CGAACAAGCGCGAAACAAACAAAGCTTCAACGC	
**6HB_B7_3gap_020**	TTGAGAACGAGGCGCAGACGGTGTACTGAG	
**6HB_B8_3gap_001**	GTAATAGGAGGAAGTTAATTGCTCCTTATTA	
**6HB_B8_3gap_002**	TGGATAGGATTGCAGAGGTCATTTTTGGCAA	
**6HB_B8_3gap_003**	GAATCGTTTATAGTCTTAGAGCTTAATAACA	
**6HB_B8_3gap_004**	GAATCCCTCAGGTCTATAATGCTGTAGTCTA	
**6HB_B8_3gap_005**	CAGAAGTACCTCCCGAAAGACTTCTTT	
**6HB_B8_3gap_006**	CAAATTGATAATCAAAAAGATTAATAA	
**6HB_B8_3gap_007**	ACAGCGGATGGCAGAAGCAAAGCGCGT	
**6HB_B8_3gap_008**	CATTTGCTGAATTTACCCTGACTACAT	
**6HB_B8_3gap_009**	CAATCTCAACACATAAATCAAAAACCT	
**6HB_B8_3gap_010**	GAAGAAATATCGTCAGGATTAGAGGCAT	
**6HB_B8_3gap_011**	AATGTACGAGGCATAGTAAAACTTTAAATAAAG	
**6HB_B8_3gap_012**	CCAAGATACATAACGCCAACTTATGCAAAGAAT	
**6HB_B8_3gap_013**	AAATAATACCACATTCAACCTCATTAAATAAAT	
**6HB_B8_3gap_014**	CAAAGATTCATCAGTTGAGTGGGAAGATAGTAG	
**6HB_B8_3gap_015**	AAAAGATGGTTTAATTTCGAGCAACAGAG	
**6HB_B8_3gap_016**	AGCATCATTGTGAATTACAAGGAATTTTA	
**6HB_B8_3gap_017**	GGCGATTTTAAGAACTGGTAATGCATACT	
**6HB_B8_3gap_018**	TCCTACCAGTCAGGACGTATTTAGGATTC	
**6HB_B8_3gap_019**	CTAAAAAATCTACGTTAAGTAGAAATGCT	
**6HB_B8_3gap_020**	TGCCACTATCATAACCCTCGGGCTTGGCTA	

Supplementary Table 4. DNA sequence for the 10HB closed ring structure.

**10HB closed ring**		
**Name**	**Sequence (5'→3')**	**Color**
**10HB_conn_001**	GGGCGATGGTCAAATATA	
**10HB_conn_002**	AAAACTTTTCCCACTACG	
**10HB_conn_003**	CGGCGAACGACATAGCGA	
**10HB_conn_004**	TCCTTGAAATGGCGAGAA	
**10HB_conn_005**	CAGGGCGCGTACAAGAT	
**10HB_conn_006**	CCTGAGCAAAAGTATGG	
**10HB_conn_007**	TTTTTATAATCAGATGAA	
**10HB_conn_008**	GGTTTAACGTCAGTGAGG	
**10HB_conn_009**	TAATATCCATTCATCAAT	
**10HB_conn_010**	TGATGGCAAGAACAATAT	
**10HB_Brid_001**	ATCGGAACTAGCATTCGCATTTTTCCGCAGGAA	
**10HB_Brid_002**	TCAATTAACGGAACGAGCAACAGAGGAAGCGG	
**10HB_Brid_003**	AGAACGTTTAGTACCTGAGTTCTTGCTCCTTTA	
**10HB_Brid_004**	GAAAGATATCAGAGGATAGCCGACGGAATTGAG	
**10HB_Brid_005**	AAGAAAATCATAATTACTAGACATAAGAATAAACACCTAAC	
**10HB_Brid_006**	TTTTCGACTCTAGACCGGATGTGCCAGTAATAAAAGAACAAAA	

**10HB_Brid_007**	AGCTCATTCCATATAAGGTCATTAGCCCTAAAACATACATGAT	
**10HB_Brid_008**	CCAAGGAAGTTTCCGTGCTTTTGGAAAAATCTAAAGAGGAGTA	
**10HB_Brid_009**	ATGCCCCCTTATTAGTAACCATCGCACTAACAACTATATATTA	
**10HB_Brid_010**	TTAATAAGTCCTGAACATAAAGTTTAAAAGTTTGAGGGACGAT	
**10HB_Brid_011**	TTGGGATTTTTGTTAACCCGTCGGATGTAAAGCCTGTTTGCGTA	
**10HB_Brid_012**	CCCTGTTATCATAACTCCAATACTGCGAAAAGGTGGCATTATGA	
**10HB_Brid_013**	GGACAGTCACCAGTGTATGGGATTTTCGCGAAACAATTTGAAAGA	
**10HB_Brid_014**	ACCGTACAAAGTTAACATACATAAAGCAGAGCCGCCCTGAATTT	
**10HB_Brid_015**	TCTTACCAGTATCATCGAGAACTGCGGGAGGTTTTTATCATATG	
**10HB_Brid_016**	GCATAAACTGGATCCCCGGGTACCGAGATACGAGTAATGAG	
**10HB_Brid_017**	CGCGAGCCAAACAGTTGATTCCCAATTTTTTCATCTACTAA	
**10HB_Brid_018**	TATACCACGATTAAACGGGTAAAATAGACCCCCCGGAGAT	
**10HB_Brid_019**	CACCACCAGGCGTTTGCCATCTTTTCACCACCCTCCACCAC	
**10HB_Brid_020**	TACCGCACAACAAGAAAAATAATATCCGTATTAACCGTTTT	
**10HB_Brid_021**	GATCGCAGTTCTCCGTGGGAACAAACGTCGGCCTGCACCGCTACG	
**10HB_Brid_022**	ATTGCATTGGAATCGTCATAAATATTCGAAGCAAAGCCCGAATTG	
**10HB_Brid_023**	ATTGTATAGGCTAAACAACTTTCAACAAAAGGAGTTCGAGGCTTG	
**10HB_Brid_024**	GGAGGGACTGTGGCAACATATAAAAGATCAACCGAATTATTGCCG	
**10HB_Brid_025**	TATTTAACTAAAGCCAACGCTCAACAGTAGGGTGAATGGAAACACAAAT	
**10HB_Brid_026**	ATTGCTGGCGAAAGGGAGCCAGCAACGCCAGGGTGGTTGTTCCGAA	
**10HB_Brid_027**	ATTCTTTTGATAAGAAAGATTAACAATACTTTTGCGGAATCACCA	
**10HB_Brid_028**	ACCGGAGTTAAAGGCCTTATCAGTCGATGAACGGTGTAAGAAACACC	
**10HB_Brid_029**	ATTACGTCACCAATGAAAATATTGATCCAGTAAGCGTCTGAAACAT	
**10HB_Brid_030**	ATTGTAATAAGAGAATGCCAACAAGGAAGCCTTAAATCATCCAAAT	
**10HB_B1_001**	TCCCTTATAAATCAACACACGACTGCAAGGCGATTCTGC	
**10HB_B1_002**	GTCTTGCAGCAAGCGGTCGCCTGGTGTAAAGTTTCCTGAGGAG	
**10HB_B1_003**	TGAACCATCACCCAAATCAAGTTTTA	
**10HB_B1_004**	TGCCGTAAAGCACTAGCACTATTAAAGAACGACCTACACATCA	
**10HB_B1_005**	CAGCAAGCTTCGTAATCATGGTCGCTCACTAATTGC	
**10HB_B1_006**	GAGTCCATCACGGTACGCCAGAACGTATACTTAATGCGCCGCTA	
**10HB_B1_007**	GAACCCTTTGTAGAAAGGGATTTTAGTTAGAACGTAACC	
**10HB_B1_008**	TGAATCGCTCACATAATTCCACACAACCTCGAATTGCATGCAAGT	
**10HB_B1_009**	AGGAAGGGAAGAAAG	
**10HB_B1_010**	CGAAAGGCGCCGCGACGTGCTTTCCTCGACAGGAACGCAAAGAGT	
**10HB_B1_011**	CCTGTCGCACTGCCTGAAATTGTTATCCATAGCTACGACGGTTTC	
**10HB_B1_012**	GTTGCGCTTGCCAGCTCCCTTCACCCACGCTGGTTCCAGTTTGGAACTCGT	
**10HB_B1_013**	TGAGCTAAGCCAACGCTGAGACGGGCGAAAATGCCCGAGATAGGGTTACAT	
**10HB_B1_014**	ACCACACCAGCGGGCGGCCCCCGATTTTTGGGCAACGTCAAAGGGCGGGAA	
**10HB_B1_015**	TTGCTTTGACGAGCATCCTGAGAAGTG	
**10HB_B1_016**	CGGGAGCTAAACTGCGCTTTCCACGGTCACGCTGCGTCAGAG	
**10HB_B1_017**	GCCGATTCAATACTTCTTTGTCCCTGAGCGGGAAA	
**10HB_B1_018**	AGGTCACCAGGCGGGGAGAGGCGGGGGTGCCCCGGAA	
**10HB_B1_019**	CCACCGAGTAAAATCGGCCTTGCTGG	
**10HB_B1_020**	CCAAAATGGATTATTTGAGTGTTGTTTGCCCCAGCAGGCAA	

**10HB_B1_021**	TGGCAGATTCACCAGTAAGAATACCTGTTTGATGGTGTTTC	
**10HB_B1_022**	AGACGCTCATGGAAATTGGACTCGTCGAGG	
**10HB_B1_023**	CTTGCCTTTAACCGAAAGGGACTAGGGCGCTGGCAAGTGTAGGTAGAAATCG	
**10HB_B1_024**	CTGGTCACGACGATTAGTAATAATTTTGACGCTCAAAAGA	
**10HB_B1_025**	TGGGTAACGCCAGGGTCCAGTGCCTGATTGGCATTAA	
**10HB_B1_026**	TACCGCCAGCCATTGCAACAAAAAACCGTCTATCA	
**10HB_B1_027**	AAAAGAACTCAAACTAGAGTCTGCTTGACGGGGAAAGC	
**10HB_B2_001**	AGGATGCCTGAGTAATGTCATATAATGTTTATTAGATACATCG	
**10HB_B2_002**	ATTCAACCGTTCTAGAACTGATTTTGCGGATGGCTGGAA	
**10HB_B2_003**	CCAAAAACAGGAACACTATCAGGTCATTGCAAGCGTAGGAAGG	
**10HB_B2_004**	TTAGCCGGAACGACGACAGTAGCGGATTATGTGAG	
**10HB_B2_005**	ATAAGCACCATTCGCATCTGCCAGTTTAGGTCAAGCCAGC	
**10HB_B2_006**	TAAAAGTACCGAGTAGATTTAGCAATAACCATCCAA	
**10HB_B2_007**	TTAACCAACATTAAGACCGTAATGGGATGAGGGGAACCAGGGTGC	
**10HB_B2_008**	ATTAAGCACAGGCATTTCGCAAATGGTTTGACCTAAATATTAAT	
**10HB_B2_009**	CATAAAGGCATTAACTGTTTAGCTATACTGCGAAGGTGTCTTAGA	
**10HB_B2_010**	CGAGTAACAAATCAGCGTTAAAATGTCAATCAATGAACGGTAATCGTGGAC	
**10HB_B2_011**	TTTCATCAATAGGAACGAAATTGTAGATAATCAGTCTGGAGCAAACAAGATAG	
**10HB_B2_012**	TAAATCATAATAAAGCTAGAACCCTGTAGGTAATTTTTGAGAGATCTCAGA	
**10HB_B2_013**	TAGTAGTACTAAATCGTCAACGCATGAGAAAGATTAATGCCGGAGAGCTAT	
**10HB_B2_014**	TGTAGATGGGCGCAAGGCAAAGACTGGCCTTCCTGTCGTTGG	
**10HB_B2_015**	TAACCGTGCCATTCAGGCTACTTTTAAATAGCAAA	
**10HB_B2_016**	GTTCTTTATTGTTGTACCAAAAACATCAATTTTGGGG	
**10HB_B2_017**	CCACTGGCCAACAGAGAGAATCGTATGTACCCCGGTTAACG	
**10HB_B2_018**	GGGCCTTCTGACCTGACTGAGAGAAAAGCC	
**10HB_B2_019**	GCGATCGCAAAGCGAATATTTCCATCAAAAATAATTCGCGTATTGGATTGT	
**10HB_B2_020**	GCTTATTTTTGAATGGGGTAGCTAAGATTCAAAAGGGAGGA	
**10HB_B2_021**	GCTTCTTTAATGCGCGCTGATAAGCCGGAGACAGTCAGAGA	
**10HB_B2_022**	AACCCTCTTCGCTATTTCTGGTATATTTTTCATTTT	
**10HB_B2_023**	CAAGTAGCTCAGCGCAACTGTTGAGAATACGTGGCAACAA	
**10HB_B2_024**	TAGTAATTGCTGAATAGCAACTAAAATTTTCTCAGAG	
**10HB_B3_001**	TAACATTACCCAAATCATGACAAGAACGGCTAAAACGAGACCA	
**10HB_B3_002**	GTAAATTGGGCTTGAAGCAAATCGGGATCGTCACTTTC	
**10HB_B3_003**	TAGGAATACCACATTGAACTGGCTCATTATAACAGAGACTCCA	
**10HB_B3_004**	AATCAAATAACTATTATAGTCAATTGAATTTAGACT	
**10HB_B3_005**	TAATGCAGAGCTTAAAATCAGGTCTTTTTAAACAGAGGGG	
**10HB_B3_006**	CTTGGACTACCACTACGAAGGCACACTAAATAAATT	
**10HB_B3_007**	ACGATAAAAAATGTCCCCCTCAAATGCTACCCTGTCGCGTTAGGA	
**10HB_B3_008**	AGCCGGAATCCGCGGAGGCAAAAGAATACCAACCTACAGAGATCG	
**10HB_B3_009**	TCAATCATCGCCTGAACACTCATCTTTCGTAATGAAGACTTCCTC	
**10HB_B3_010**	GGATAGCGCCTCGTTTACATAGTAAAACATTATTACGTTAATAAAACGACGCC	
**10HB_B3_011**	GTAATAGTAAACCAAAAACGCCAATCATCAGTAGGACGTTGGGAAGAGAAC	
**10HB_B3_012**	GTGTCGAAACGAGGCGAGTAATCTACGTAACAGAATTACCTTATGCGTATT	
**10HB_B3_013**	TTGTATCATAAGGGAAAGGCTGGCGTGAATAATAATTTCAACTTTAAGTGC	

**10HB_B3_014**	GAAAACGAGAATAAACCTGCTCCAAGAAGTTTTGCCAGTTCA	
**10HB_B3_015**	TAAATCACAAAGCGAACCAGCAACCGGAGTTACTT	
**10HB_B3_016**	ATGGGCGCATCCGAACTGACCAACAGTACAAAGCGAT	
**10HB_B3_017**	CTCAAAAATACCGAACAAAATCTACAGGTAGAAAGATAAGG	
**10HB_B3_018**	TTACAGCAGAAGATAAACCAGTCTGAGATT	
**10HB_B3_019**	ACAGGTCTTAATTCGATACATATAGCGAGAGGCTTTTGCAAATTATTCAAC	
**10HB_B3_020**	GAAACCGCCTGCAACATCATTGTAAGCTGCTCATTCATGAC	
**10HB_B3_021**	AGCGCTGAGAGCCAGCGATGGTTGGCTTGCCCTGACGCAGA	
**10HB_B3_022**	CACGAGAGTACCTTTAAAGACTTTACGAGGCCAGACG	
**10HB_B3_023**	AACCGAGGGTAGACCGGAAGCAAGTGAGGCGGTCAGATTT	
**10HB_B3_024**	CACAGCGAAAGACAGCGCTTTGACATCAAGCAGACGG	
**10HB_B4_001**	TAAGAGTAACAGTGCCCTAACGGGTCAGACACCACCGGAATAA	
**10HB_B4_002**	AGAGGCTGAGACTCCTTTAGGAGATAGCAGCACCGGTCA	
**10HB_B4_003**	CTCAGAGCCACCATTGTGCCGTCGAGAGGGTATCTGGCCCACG	
**10HB_B4_004**	AACCTTAAAAAAAAAGGCTCCAGTTTCAGGTAAATG	
**10HB_B4_005**	TTTTCAGTAGTTGTTCACGTTGAAAAGAAAGGATTTGTCG	
**10HB_B4_006**	TGTATCGGCAATCACCGGAACAGCCGCCCATTGAC	
**10HB_B4_007**	TGTAGCAGACGTTACGGAGTGAGAATATCTCCAACAGCTTGGATA	
**10HB_B4_008**	ATATTCAGAGGCAGCCGCCTCCCTCAGCAGAGCCTGTAGCGCAAG	
**10HB_B4_009**	TCATTAACCGCCAGACCCTCAGAACCGTAATCAAATTTTCGTAAT	
**10HB_B4_010**	AATTTTCTACAAACTACCGTAACACGCCACCCATCACCGTACTCAGGCATC	
**10HB_B4_011**	TCTTTCCATTCCACAGAAGCCCAACTCAGAACAAGTATAGCCCGGAAAATA	
**10HB_B4_012**	AGGAGGTTCAAACAAAATAAGTTTGTATAAACTTGCTCAGTACCAGGCAAC	
**10HB_B4_013**	CAGAGCCGAGCCAGAATATGATACAGCCTATTTCAAGGATTAGGATTAGGGAA	
**10HB_B4_014**	AAAGGAATTGCGAAGTCAGACGAAACGATCTAAAGTACAACT	
**10HB_B4_015**	TAATTTTCGCCGACAATGACGGTCAGTGGGCCTTG	
**10HB_B4_016**	TAGGCTTTTGGGAAAGCGCAGTCTACCAGAACAGAGC	
**10HB_B4_017**	CAGTTGCTGAACCTCATAGGTGTTCAGAACCGCCACCTAGG	
**10HB_B4_018**	TATAACCCTCAATCAATTGATATCGCCACC	
**10HB_B4_019**	CATAACCATACCGAGGATAGCACAGCCCTCATAGTTAGCGTTTCCCCCTCA	
**10HB_B4_020**	TTTTGAAAGGAATTGACGGGGTTAGTTAATGCCCCCTGGAG	
**10HB_B4_021**	CAGTATCTAAAATATCTCAAGAGGGAACCTATTATTCATAC	
**10HB_B4_022**	TCATCGGTCGCTGAGGTGAATTTCCATGTACAACGCC	
**10HB_B4_023**	AGTGCCTTTAGCAACAACCATCGTCAGTTGGCAAATCGGA	
**10HB_B4_024**	GGTTAGCGACAGAATCGTTTTCACTGGTATAAATCC	
**10HB_B5_001**	AAGGCGTCTTTCCAGAGTCTTACCTGTTCACAATCAAGAAAA	
**10HB_B5_002**	TTTTTGTTTAACGTCATTAATTACCGACAAAAGGTACAA	
**10HB_B5_003**	GAGCAAGAAACAAGACGCATTAGACGGGAGAGAAGTAAAAATC	
**10HB_B5_004**	TTTGGTGAAAAAAGGGCGACATAACGCAAGCAAACG	
**10HB_B5_005**	AGCAATAGACTTGGGTTTACCAGCGCTAAGTTTCTCCTTA	
**10HB_B5_006**	CTACGCCTGATTTACGAGCATGCCTTATCAATCATT	
**10HB_B5_007**	GGAAACGTATGTTAAGACACCACGGAACAAAGACTTATCACGCAC	
**10HB_B5_008**	AGGCTTACAATAGCATCGGCTGTCTTTTAGAAACGCTAATGACGA	
**10HB_B5_009**	CGCGAGGTCGTAGGATTCCAAGAACGGCATCCTATTTATCAAAAG	

**10HB_B5_010**	TAGAAAATCCAGAAGGAGTAAGCAAGATAACCAGGGTAATTGAGCGCATAG	
**10HB_B5_011**	TTACGCAGCAATAATATACCGAAGTAAGCCCATGAACACCCTGAACAGATA	
**10HB_B5_012**	ACCGCGCCTCCGGTATTATCCTGAACCTAATTTGGAATAACATAAAAACACAA	
**10HB_B5_013**	TATTTTCACGTTTTAGTTTTGCACAACAGCCAAAAATAGCAGCCTTTAAAT	
**10HB_B5_014**	TCACAATCAATATAAAGCAAATCGGCATGATTAAGAATTTTG	
**10HB_B5_015**	TTCATATAGCCATTTGGGACAAACGCTAATATAGA	
**10HB_B5_016**	TAGGTTGCTACGAACCTCCCGACTAAGCAAGACCAAG	
**10HB_B5_017**	GAAAGAGCCGTCAATAAAGTCAGCACAAGAATTGAGTCCCT	
**10HB_B5_018**	CATCATTTGAGGATTTAATTAACATAATAA	
**10HB_B5_019**	ACCAGTACGTCACCGCTATCTACGGAATACCCAAAAGAACTAGACTGAAAT	
**10HB_B5_020**	CAAGACAACTCGTATTACAGAGACCAGTTACAAAATACCAG	
**10HB_B5_021**	TAATTGCCCGAACGTTAAAAATGTATTATTTATCCCAAAGA	
**10HB_B5_022**	ATATACCATTAGCAAGCATTAAATAAGAAAAAAACCGA	
**10HB_B5_023**	TTCTAAACAAATTAGAGCCAGCTTAGACTTTACAAAGGG	
**10HB_B5_024**	CCTTTCTGTCCAGACGCAGAACGCAATTTTCTAAGAA	
**10HB_B6_001**	TAACTCCGGCTTAGGTTAGAGACTTATCAACGCCTGATCTTAA	
**10HB_B6_002**	TTTTAGTTAATTTCATCTTCTGCCTGATTATCAGA	
**10HB_B6_003**	ATCAAAACAGGAGAAACAATAGTTACAATTTCATT	
**10HB_B6_004**	TGATGCTGATGCTTTAATGGTTTGAAAAGCGGAACTGAA	
**10HB_B6_005**	TAGCTTAGATTAAGAGACAAAGAACGCGAG	
**10HB_B6_006**	CGTTATAGTAAAAAAGCCTGTTTTGTAATTCGCCA	
**10HB_B6_007**	ATCAATACTTTTTTCTTTGAATACCAAACGGATTAATTATTAGGG	
**10HB_B6_008**	TGAATTACTATGTGAGTAGGTCTGGGGTTATACGTGTGATAAATAAGACAA	
**10HB_B6_009**	CAAAATCGCGCAGAGGTACATCGGAAATAATATACTTTTATCA	
**10HB_B6_010**	GATGAAACAAACATCAATTTTCCCTTAGAA	
**10HB_B6_011**	TTGAGAATTAGGCAGAGGCCAACCTTTTTACATAA	
**10HB_B6_012**	TACCTTTCGAATTACAAAATTAATTACATTCTGTAAATAATAG	
**10HB_B6_013**	TATACAGTAACAG	
**10HB_B6_014**	ATTAAGAGTCCGTCGCTATTAATTAAGAAAATTCATTTCAATTA	
**10HB_B6_015**	TTAAACCACCAGAAGGTACCGACTAACTATATGTAAAATTT	
**10HB_B6_016**	TAATGGATGCACGTAAAATCATGAATAACCTTGCTTTAA	
**10HB_B6_017**	ATAATCCTGATTGTTTGGATAGAA	
**10HB_B6_018**	AGAGAACCTACATTTTCGAGCCAATCATTTTGCGGAGCGT	
**10HB_B6_019**	TCATATTACCTAAAAAATCCAATCGCAACGCTGAGGCGTAGATTTTCA	

Supplementary Table 5. DNA sequence for the 6HB closed triangle structure.

**6HB closed triangle**		
**Name**	**Sequence (5'→3')**	**Color**
**6HB_tri_conn_001**	TGATTATTACCTGATAGATTAAGACGCACACAACACAACAGC	
**6HB_tri_conn_002**	AGACGGGTACGAGCTATCCGCTCACAATTCCTGAGAAGAGTC	
**6HB_tri_conn_003**	TTCATTTCAGCCCTTCACCGCCTGGCCCCAAAGGGCGAAAAGGGTTAGA	
**6HB_tri_conn_004**	ACCTACCACTTCTGAATAATGGAAACCGTCTATCA	

**6HB_tri_B0_001**	TAAATAAGAACAGGGAAGCGCATTAGACATAGATAATTACCG	
**6HB_tri_B0_002**	ATTTCAACGGGGATGTGCTGCAAGGCGACGCTTAAGTAGCCA	
**6HB_tri_B0_003**	TCATTCAGTTTTCGCAAATGGTCAATAAGTAGCAATTAAACA	
**6HB_tri_B0_004**	CAGAGAGACCAACGCAGCTACAATTTTATCTTCAGCTAATGC	
**6HB_tri_B0_005**	TACGCCACCTCAGGTTTGAGGGGACGACGAGGTAATCGTAAA	
**6HB_tri_B0_006**	AGTTTGATTAGAGAGGAAGCAAACTCCAACCCACATTCAACT	
**6HB_tri_B0_007**	CTGCTGCCACCCTCAGCGGAGTGAGAAATGCCACTCCGCGAC	
**6HB_tri_B0_008**	GTGCCCAAAGTTACAAAGACAAAAGGAGAGCCGCCAGTAACA	
**6HB_tri_B0_009**	ACATGCTGAATCTTAATAACATAAAAATAAACACCATAAACA	
**6HB_tri_B0_010**	TGAACCAGTATCGGGCTGGCGAAAGGCAAGGATAAGAATCGA	
**6HB_tri_B0_011**	GAATAAGGTCAGGACCATTAGATACAGAATAAGGCGATTTAG	
**6HB_tri_B0_012**	CAGTTTCAGAGCCACGCCACCCTCAGAACCCCATGTTACTTA	
**6HB_tri_B0_013**	CAGCGCCCAGAAGGTAAGCAGATAGCCGAACGTATAAACAGT	
**6HB_tri_B0_014**	AAAATACGTATAGAAAGGAACAACTAAAGCCCACGTCTGGCC	
**6HB_tri_B0_015**	ACCACCACCGCGACATTCAACCGATTGAAATCAAGCAGCAGC	
**6HB_tri_B0_016**	GATATATTCGGTCGGACCAGTAATAAAAGGGACATCATAACC	
**6HB_tri_B0_017**	TTAGCGTCAGACTGCAACAGTGCCACGCTGAGAGCTTTGCCT	
**6HB_tri_B0_018**	TGAGGATTTAGAAGTTTATTTTCATCGTAGGAATCATACATT	
**6HB_tri_B0_019**	CTACAGGGCGCGTATAATTCGCGTCTGGCCTTCCTTGCGCCG	
**6HB_tri_B0_020**	TTGATTAGTAATAAATTGAATCCCCCTCAAATGCTTACTTCT	
**6HB_tri_EB1_001**	GGGTACCTCGAGGTAGTGTTGTTCCAGTAGGCGAAGAGGCGG	
**6HB_tri_EB1_002**	TGCCAAGGAGCGGGCAAACGGCGTATAAATCAAAAGAAGAAA	
**6HB_tri_EB1_003**	GCCAGGGGCGTAACTAAATGTGAGCGAGGGGCGCATTCCGGC	
**6HB_tri_EB1_004**	CTGTGTGGGCCCACGTGGACTCCAACGTTGAGAGAACCAGTG	
**6HB_tri_EB1_005**	CTGCCCGCATTAATCGAAATCGGCAAAATCCCTGATTGACCG	
**6HB_tri_EB1_006**	GCAACTGCTGGTGCACGTTGGTGTAGATTAACAACAGCGGTC	
**6HB_tri_EB1_007**	GCCTAATATTGGGCTGGTTTGCCCCAGCTTGGAACCAAGTTT	
**6HB_tri_EB1_008**	TTTGCGTGAGTGAGAATTCGTAATCATGCCCAAATAAGAGTC	
**6HB_tri_EB1_009**	CCAGCTGCTTTCCAGCCTGCAGGTCGACAGGAAGGGAATAGC	
**6HB_tri_EB1_010**	ACCGCTTTTGGGAAAGTCACGACGTTGTCAAGTGTCCGTCGG	
**6HB_tri_EB1_011**	TTTGATGAGGGTTGGCCGTAAGCGAGAATCTAGAGGCGCTCA	
**6HB_tri_EB1_012**	TAATGGGGTGGGAACGCTAGGGCGCTGGAAAACGAAGGCTGC	
**6HB_tri_EB1_013**	CGTGCATTCAACATCACCACACCCGCCGTTAAGTTTCGCTAT	
**6HB_tri_EB1_014**	CAAGCGGAAAGAACTACGTGAACCATCAGTCATAGCTGGGGT	
**6HB_tri_EB1_015**	CCGAGATGTGGTTCGAATCGGCCAACGCTTGCGTTGATCCCC	
**6HB_tri_EB1_016**	ATTCTCCATAGGTCCGGAAACCAGGCAAAGCGCCCTGTCGTG	
**6HB_tri_EB1_017**	GCTTTCACTGCCAGAAGATCGCACTCCAGGCCTCTGGGTAAC	
**6HB_tri_EB1_018**	CACTATTTCCACGCGCCAGGGTGGTTTTGTAAAGCCTGTTTC	
**6HB_tri_EB1_019**	TTTGGGGGAGCTCGCTAACTCACATTAAGCGGGGAAATCCTG	
**6HB_tri_EB1_020**	GCGAAAGCTTGCATGTCGGGAAACATTCGCCATTCCGGCCAG	
**6HB_tri_EB1_021**	ACGCTGCTTTTCCCGGGCGATCGGTGCGGCCAGCTTCGTAAC	
**6HB_tri_EB1_022**	GGGCGATAAATTGTCGGAAGCATAAAGTTCTTTTCGTTGCAG	
**6HB_tri_EB2_001**	AGAACCGGCCTTGCGACTGGATAGCGTCTACGAGGCAACATT	
**6HB_tri_EB2_002**	GGCGCATGCAACAGGCAAAAGAAGTTTTCCAGACGGTTGGGA	

**6HB_tri_EB2_003**	TGACCAATCAATCGCACCCTCAGCAGCGTACAGAGCTCATCT	
**6HB_tri_EB2_004**	GCCGGAAGTCACACCTGAGGCTTGCAGGTCCATTACACCAAC	
**6HB_tri_EB2_005**	AACACCAGTAGAAAACGCCAAAAGGAATCAATACTAACTCAA	
**6HB_tri_EB2_006**	TTCAACTTCTACGTATAACCCTCGTTTAGCCAGAGTACCGCC	
**6HB_tri_EB2_007**	TACCAAGAGAGGACAGATGAAGAAATACGCATCGGAACGAG	
**6HB_tri_EB2_008**	ATCATCGGAAAGAGTTCATGAGGAAGTTGAGTTAATGGCAGA	
**6HB_tri_EB2_009**	ATTACAGGAACGAGATTACCCAAATCAAGTAGAAGGCGGAAT	
**6HB_tri_EB2_010**	AGAAAAATTAATCACTGACCTTCATCAAACAATATGGGGTAA	
**6HB_tri_EB2_011**	TTGACCCCCAGCTGGCTCATTATACCAGATGCGATCAGACCA	
**6HB_tri_EB2_012**	CTAAAACCCTGATACAGACGGTCAATCATTTACATAGGCCGC	
**6HB_tri_EB2_013**	AATGCAGAATATTCCATCACTTGCCTGACGTAACAGACGAGA	
**6HB_tri_EB2_014**	AGAGCAAATGTTTATGGTAATATCCAGAGAGTAATGTTTAAT	
**6HB_tri_EB2_015**	AAACCAAAATAGCGAGGGTAGCAACGGCAAAGACACTACATT	
**6HB_tri_EB2_016**	GGACTAAGGATCGTTCTGAAATGGATTATAAGGGAGATTTGT	
**6HB_tri_EB2_017**	CGTCATAATACATAGATTCATCAGTTGATTGCCCTAAGCTGC	
**6HB_tri_EB2_018**	TAGTAAACACTATCTAATAAAACGAACTTGAGATGCTTGACA	
**6HB_tri_EB2_019**	AGGCTTTTGAAAAACGCTCATGCGGTGTATTTAAGAACGATTA	
**6HB_tri_EB2_020**	TTTTGCGAGACTTTGCAAAAGAATACACCAACGGAACCGAAC	
**6HB_tri_EB2_021**	ACTATCGGATATTCTAGTAAATTGGGCTAACGGAACATAGTA	
**6HB_tri_EB2_022**	AGCCATTAGGCTGGTTGTGAATTACCTTTCAGGACACGATAA	
**6HB_tri_EB2_023**	TTGACGCCTTTGAACGCGAAACAAAGTATAAAACAGCTTTGA	
**6HB_tri_EB2_024**	TTCACCACGAGGCGAATTGTGTCGAAATACGAAGGAACGGGT	
**6HB_tri_EB3_001**	AACACCCACTAATACAAATCAGATATAGAAATCAACCTAATT	
**6HB_tri_EB3_002**	CTTTTTAAAATCTAATCAGTAGCGACAGGGGAGGGGGTTTAC	
**6HB_tri_EB3_003**	AGCAAGATCAATCAGCCGGAAACGTCACGAATTATCGGAATA	
**6HB_tri_EB3_004**	AGAGAGAAATTGAGAGGCGTTTTAAAATCACCAGTCAACAGT	
**6HB_tri_EB3_005**	TACCCAATTTGTCATATTCATTAAAGGTCAATGAACAAATAT	
**6HB_tri_EB3_006**	TATGTTAAACATATTTTGGGAATTAGAGCCAGCAGCGAACCT	
**6HB_tri_EB3_007**	CGATTTTTACAAAAGGTTTTGAAGCCTTAAGGCTTTAGGAGC	
**6HB_tri_EB3_008**	AGTTTATAAGAACTAAATAGCAATAGCTTGAACCTACCATCG	
**6HB_tri_EB3_009**	AGGTGGCGCAAACGCAAGAATTGAGTTAGGCAAATAGCACCA	
**6HB_tri_EB3_010**	TGCCAGTTTGTTTAAGTCAGAGGGTAATATATCTTATCCGGT	
**6HB_tri_EB3_011**	TGCTATTTAGCAAGGATTAGAGCCGTCAGGGAGAAGCCTTTA	
**6HB_tri_EB3_012**	ATATTGACACCGTAAAGCATCACCTTGCATCTTACAACGGAA	
**6HB_tri_EB3_013**	ACCGACTTAGCAAGATATCTGGTCAGTTAGCCCAATACGCAG	
**6HB_tri_EB3_014**	CCCGACTGAACGCGGAAGGTTATCTAAATGAGCGCTAAGAAA	
**6HB_tri_EB3_015**	CGCCCAATTGCACCCTAACGAGCGTCTTAATAGCATTAACTG	
**6HB_tri_EB3_016**	ATAGCAGCGGAAATCAATCAATAGAAAACAATAATCGAAGCC	
**6HB_tri_EB3_017**	TTACCATTGAGCCAAAAAGAAACGCAAATCCTTATTAATAAG	
**6HB_tri_EB3_018**	ATTCTAATGCGGGATAAACAGCCATATTATTTACATACATAA	
**6HB_tri_EB3_019**	AAATGAAAGAAAAGAAACCGAGGAAACGTTCATATAAGGTAA	
**6HB_tri_EB3_020**	CAAACCCAACAATGGGCATGATTAAGACGACACCACACCGTC	
**6HB_tri_EB3_021**	TGAAAGGTAACCCATAGAAAATATCCCAATCCAAATAATATC	
**6HB_tri_EB3_022**	ACTAACATGAACAAACGTCAAAAATGAATCCAGAGGATTAGT	

**6HB_tri_PB1_001**	TACCAAAATCCTCGTAGCTCATTTTTCGGTTGATCAGGTC	
**6HB_tri_PB1_002**	TAGCAAAAAAAGGGAATTTTGTTAAATTGTATATGCCGGA	
**6HB_tri_PB1_003**	TAGTAGTAGTGTTTTTTATAGTCAGATAAGAGGGAATATA	
**6HB_tri_PB1_004**	TTTTCATCCATCACAATGACCATAAATCTAATTCGGATAAGA	
**6HB_tri_PB1_005**	TCATATATGAGAGTCCATATGTACCCTAACCAACGTATAA	
**6HB_tri_PB1_006**	AAAGGGTGGCTATTTAAACAGGAAGAATTCGCAACAGGAG	
**6HB_tri_PB1_007**	TTTTAAATATCCAATAAATCATACGGTACGCCGGATTGTAAA	
**6HB_tri_PB1_008**	TTCCATATTTTGCGGATATCGCGTTTAAAAATCGTAAAAG	
**6HB_tri_PB1_009**	ATTGCCTTTTAAATGACCCTGTAATACTTTTACGAGCATAGG	
**6HB_tri_PB1_010**	GAGGGTAAGAAAGGCTAAAGCCTCAGAGCATGAGCTAATTAA	
**6HB_tri_PB1_011**	ATGCTGTAGCTCAACCGTTCTAGCTGATACCATCAACAAAGAAT	
**6HB_tri_PB1_012**	GGTCATTAACAGTTGGCGAGCTGAAAAGGTGCCACCGAAGGT	
**6HB_tri_PB1_013**	ACTAGCATCAAAAACTATGGTTGCTTTGGCGGGAGATAGAACCC	
**6HB_tri_PB1_014**	CAGAAAATTAAATCTAGAATCAGAGCGGAAAGCTAAAAGATTCA	
**6HB_tri_PB1_015**	AATATTTAAATTGCATCAAAAAGATAGCAAAGCAGAATC	
**6HB_tri_PB1_016**	CCGAAAGCTGACTATATAATCAGTGAGGGCATCAATAAGTTTCA	
**6HB_tri_PB1_017**	AAGCGAAAAACGAGGCAAATTAACCGTTCCTGTTTTAGATTT	
**6HB_tri_PB1_018**	AACGCCATGTCAATTGGAGCAAACAAGAAAATTTTAGCCTTT	
**6HB_tri_PB1_019**	ATTTTTGGCCCCAATTGAGAGATCTACATGTAGGTAATCGGTTG	
**6HB_tri_PB1_020**	CGTTAATTTTTAGACAGGAACAGGCAAGGTATGATATTCAACATG	
**6HB_tri_PB1_021**	CTTTACCACTTCAAATGGCTTAGAGCTTGTGTCTGGTCTACTAA	
**6HB_tri_PB1_022**	GTTCAGACCAGACCGTACCTTTAATTGCGAACGAGAGCTATA	
**6HB_tri_PB1_023**	CGTGCTTACATTATGCAATGCCTGAGTAATGAAGGCTATAAT	
**6HB_tri_PB1_024**	GCCGATTTTAAGCAACGGAGACAGTCAAATCAAATTAAAGCA	
**6HB_tri_PB1_025**	CTGAGAAGCATTAACATGCAACTAAAGTACGAATTGCTAAGC	
**6HB_tri_PB1_026**	AGTCTGTTTGGGGCATTCCCAATTCTGCTCCTTTTAGCTTCA	
**6HB_tri_PB2_001**	CCGCCACCATAGAACCAACAACCATCGGAATTGCTTTCAA	
**6HB_tri_PB2_002**	TAAGTATAGACAATATAAACAGCTTGTCCAAAAACGTTAG	
**6HB_tri_PB2_003**	GGGTTTTGGATAGCCACCAGATTTATCAGCTTGTTAATGC	
**6HB_tri_PB2_004**	AAAGTATTCCAGCAGAGCGTTTGCCACCACCCTTCACAAA	
**6HB_tri_PB2_005**	TAATGCCCCGCCTGTAGCGCGTTTTCATGAGCCGCTTGACAG	
**6HB_tri_PB2_006**	AGGAACCCATTTTCTCGTTGAAAATCATACCGAGAATACG	
**6HB_tri_PB2_007**	AACGCCTGACGATCTCCTTTAATTGTATCGGGCCACC	
**6HB_tri_PB2_008**	CCAGTAAGATCCTCACCTCAGAGCCGTCTTTTCTACCGAA	
**6HB_tri_PB2_009**	AATAAGTAGGCAGGAGCCACCACCCTCACGGCATTGGTCAGT	
**6HB_tri_PB2_010**	TAAATGAATGTACCGTAGGTGTATCACCGTAAGCGTAATAGT	
**6HB_tri_PB2_011**	TAGCGTATAGCATTCCAGGCGGATAAGTGCCTTAGTCTCTTT	
**6HB_tri_PB2_012**	CAAATAACGTCATACGAGACTCCTCAAGAGATTAAAAAATAA	
**6HB_tri_PB2_013**	GAGGTTGTTTAACGTATTTCGGAACCTAGTGAGGCTTCGGTC	
**6HB_tri_PB2_014**	TAATAATACAATGACCTTCTGACCTGAACTCAGGAGAGCCCAAT	
**6HB_tri_PB2_015**	GGCTCCAAATTTCTTTTTTGAATGGCTAGTCGAGAGCAAACTAC	
**6HB_tri_PB2_016**	ACCGGAACACCGGACTAAAACATCGCCAAGGATTAGTTACCGTT	
**6HB_tri_PB2_017**	ACCGCCACCTTATTAAGATAAAACAGAGTTATTCTTACTGGT	
**6HB_tri_PB2_018**	TGCGCCGTTTTTCAGTATGGGATTTTGCGGATAGCAGTTTAGTA	

**6HB_tri_PB2_019**	CGAGGTGAAAGGAGAAAGTTTTGTCGTCCACCAGTAGGTTGATA	
**6HB_tri_PB2_020**	TCAAAATCCGCCTCTTAAAGCCAGAATGGAAAGCCTCATAGT	
**6HB_tri_PB2_021**	ATAGCCCCCCTCAGTCAGACGATTGGCCCAGGAGTGGAAACATG	
**6HB_tri_PB2_022**	AACAGAGCTCAGAACCCACCCTCATTTTCAGTAAACAACGAA	
**6HB_tri_PB2_023**	TGGCACAGCCCGGAATAACACTGAGTTTCGTTTTCCAGAAAA	
**6HB_tri_PB2_024**	GCGAACTCTCAGTACCACAGACAGCCGCAGTCTCTGAATGATTAGCG	
**6HB_tri_PB2_025**	CGAACCAAAGAGGCTATGGCTTTTGATGATATTGATATCAGA	
**6HB_tri_PB2_026**	ATTAACACCCTGCCGGGTCAGTGCCTTGGCCAGCACACCAGA	
**6HB_tri_PB3_001**	CCTAAATTAAATCCTCCAAGTACCGCGATAAGTACAAAAG	
**6HB_tri_PB3_002**	ACGCGAGAACATTATCTGTCTTTCCTAATTTACAGAGGCA	
**6HB_tri_PB3_003**	ATATAACTGAATTATACGTCAGATGACGGGAGACCTTTTT	
**6HB_tri_PB3_004**	ATAGGTCTCAATATAAAACAGAAATAAAAATACCAAGAAAAC	
**6HB_tri_PB3_005**	AATAGTGGGATTATATATCAA	
**6HB_tri_PB3_006**	AGAAAAAGAATTCTGTATCAACAATAACTCATCCGACAAC	
**6HB_tri_PB3_007**	ATAAAGCCGCCAGTATATCCCATCCTTATCATTTAAAAGT	
**6HB_tri_PB3_008**	AATCAATAATGCTGATGCAAATCCAAGAAACGTAACAATCAA	
**6HB_tri_PB3_009**	GTCGCTATATTACATTGATTGCTTTGGAAATTGATGATGG	
**6HB_tri_PB3_010**	GATAGCTGCAAAAGCGAATTA	
**6HB_tri_PB3_011**	GTAAAGTCCTGTTTATGAAATACCGACCGTGAACAATTGAGA	
**6HB_tri_PB3_012**	TTTTCGAAACGCTCATCAAATATATTTTAGTTTAATTTCCAA	
**6HB_tri_PB3_013**	TAATGGAAACAGCAACGCCAACATGTAAGAGAATCGGACAAAGA	
**6HB_tri_PB3_014**	AAAATTATAATTAATCTACCTTTTTAACCTCTTATCAGCGTA	
**6HB_tri_PB3_015**	AGAACGCAGCCGTTTATTAGACTTTACATGATAAATTAATTACT	
**6HB_tri_PB3_016**	AACAAGATATTAAATTGCCCGAACGTTATAATTTCATTACCAGT	
**6HB_tri_PB3_017**	ATGTAGAAACCAGTACCTTTTACATATATACACACCAGA	
**6HB_tri_PB3_018**	ATAACGGAGGTTTACATCATATTCCTGACGGCTTAGTGTAAATC	
**6HB_tri_PB3_019**	AAATCGCTGCACGTAATCCTGATTGTTTAATTTATACATAGC	
**6HB_tri_PB3_020**	ACAAGCAGCCTGTTTCCAGACGACGACAGGAATCAAAGGCGT	
**6HB_tri_PB3_021**	GAACGGGAAAATAAATAAGAGAATATAAACAAATTCTCTTCTGA	
**6HB_tri_PB3_022**	TAATCGGCATTTTGCGGAACAAATCGCAACCATATTTAATACATA	
**6HB_tri_PB3_023**	GATTTTCATTCGCCTTAACAATTTCATTCTTGCTTCGTTGGGTT	
**6HB_tri_PB3_024**	AATTATTGCAGAGGAAGATGATGAAACACTTGAAACAAAATC	
**6HB_tri_PB3_025**	TCGTATTTAATGGTTGTATCATATGCGTTATAGTACCGCCTG	
**6HB_tri_PB3_026**	TTGAGTAAAACTTTTACAGTAGGGCTTAATTTTTAGGCGAGC	
**6HB_tri_PB3_027**	AGGAGCGATATGTAATATGTGAGTGAATAACTGAATTAAACA	
**6HB_tri_PB3_028**	CAATTCATGAGAGATTTCCCTTAGAATCAACATCAAGTTACA	

Supplementary References

(1) Lee, J. Y.; Lee, J. G.; Yun, G.; Lee, C.; Kim, Y.-J.; Kim, K. S.; Kim, T. H.; Kim, D.-N., Rapid Computational Analysis of DNA Origami Assemblies at near-Atomic Resolution. *ACS Nano* **2021,** *15*, 1002-1015.

(2) Douglas, S. M.; Marblestone, A. H.; Teerapittayanon, S.; Vazquez, A.; Church, G. M.; Shih, W. M., Rapid Prototyping of 3d DNA-Origami Shapes with Cadnano. *Nucleic Acids Res.* **2009,** *37*, 5001-5006.
